# Long-read sequencing and *de novo* assembly of a Chinese genome

**DOI:** 10.1038/ncomms12065

**Published:** 2016-06-30

**Authors:** Lingling Shi, Yunfei Guo, Chengliang Dong, John Huddleston, Hui Yang, Xiaolu Han, Aisi Fu, Quan Li, Na Li, Siyi Gong, Katherine E. Lintner, Qiong Ding, Zou Wang, Jiang Hu, Depeng Wang, Feng Wang, Lin Wang, Gholson J. Lyon, Yongtao Guan, Yufeng Shen, Oleg V. Evgrafov, James A. Knowles, Francoise Thibaud-Nissen, Valerie Schneider, Chack-Yung Yu, Libing Zhou, Evan E. Eichler, Kwok-Fai So, Kai Wang

**Affiliations:** 1 Guangdong-Hongkong-Macau Institute of CNS Regeneration, Jinan University, Guangzhou 510632, China; 2 Ministry of Education Joint International Research Laboratory of CNS Regeneration, Jinan University, Guangzhou 510632, China; 3 Co-innovation Center of Neuroregeneration, Nantong University, Nantong 226001, China; 4 Zilkha Neurogenetic Institute, University of Southern California, Los Angeles, California 90089, USA; 5 Department of Genome Sciences, Howard Hughes Medical Institute, University of Washington, Seattle, Washington 98195, USA; 6 Genetic, Molecular, and Cellular Biology Program, Keck School of Medicine, University of Southern California, Los Angeles, California 90089, USA; 7 Wuhan Institute of Biotechnology, Wuhan 430000, China; 8 Department of Pediatrics, The Ohio State University, and The Research Institute at Nationwide Children's Hospital, Columbus, Ohio 43205, USA; 9 Nextomics Biosciences, Wuhan 430000, China; 10 School of Chemical Engineering and Pharmacy, Wuhan Institute of Technology, Wuhan 430000, China; 11 Center for Tissue Engineering and Regenerative Medicine, Union Hospital, Huazhong University of Science and Technology, Wuhan 430022, China; 12 Stanley Institute for Cognitive Genomics, Cold Spring Harbor Laboratory, New York, New York 11797, USA; 13 USDA/ARS Children's Nutrition Research Center, Department of Pediatrics, Department of Molecular and Human Genetics, Baylor College of Medicine, Houston, Texas 77030, USA; 14 Departments of Systems Biology and Biomedical Informatics, Columbia University, New York, New York 10032, USA; 15 Department of Psychiatry & Behavioral Sciences, Keck School of Medicine, University of Southern California, Los Angeles, California 90033, USA; 16 National Center for Biotechnology Information, U.S. National Library of Medicine, Bethesda, Maryland 20894, USA; 17 Department of Ophthalmology, The University of Hong Kong, Hong Kong, China; 18 State Key Laboratory of Brain and Cognitive Sciences, The University of Hong Kong, Hong Kong, China

## Abstract

Short-read sequencing has enabled the *de novo* assembly of several individual human genomes, but with inherent limitations in characterizing repeat elements. Here we sequence a Chinese individual HX1 by single-molecule real-time (SMRT) long-read sequencing, construct a physical map by NanoChannel arrays and generate a *de novo* assembly of 2.93 Gb (contig N50: 8.3 Mb, scaffold N50: 22.0 Mb, including 39.3 Mb N-bases), together with 206 Mb of alternative haplotypes. The assembly fully or partially fills 274 (28.4%) N-gaps in the reference genome GRCh38. Comparison to GRCh38 reveals 12.8 Mb of HX1-specific sequences, including 4.1 Mb that are not present in previously reported Asian genomes. Furthermore, long-read sequencing of the transcriptome reveals novel spliced genes that are not annotated in GENCODE and are missed by short-read RNA-Seq. Our results imply that improved characterization of genome functional variation may require the use of a range of genomic technologies on diverse human populations.

## Abstract (web summary)

Short-read sequencing has inherent limitations in the characterisation of long repeat elements. Shi and Guo *et al.* combine single-molecule real-time sequencing and IrysChip to construct a Chinese reference genome that fills many gaps in the reference genome, and identify novel spliced genes.

## Graphical abstract

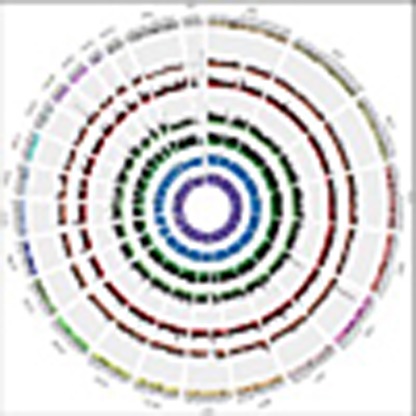

The advent of next-generation short-read sequencing paved the way to characterize the genomes of thousands of species, and had enabled *de novo* assembly of a few individual human genomes^1^,^2^. However, these assemblies may have inherent technical limitations in characterizing repeat elements that span longer than the read length^3^,^4^, yet repeats and segmental duplications are known to cover approximately half of the human genome. For example, a formal analysis of the *de novo* sequence assembly generated from the genome of a Han Chinese individual and a Yoruban individual showed that 420.2 Mb of common repeats and 99.1% of validated duplicated sequences were not present, which resulted in missing thousands of coding exons in the genome assembly^3^. Therefore, the use of additional genomic techniques, such as fosmid pooling^5^ and long-read sequencing^6^, may be necessary to better characterize complicated genomic regions in human genomes.

Previous studies reported that pervasive genetic differences exist across different ethnicity groups, especially on structural variants^7^,^8^,^9^. For example, through reconstruction of the ancestral human genome, it was reported that megabases of DNA were lost in different human lineages and that large duplications were introgressed from one lineage to another^8^. In addition, genomic elements that are absent from reference genomes may be present in personal genomes^10^,^11^. For example, a study estimated that a complete human pan-genome would contain ∼19–40 Mb of novel sequence not present in the extant reference genome^12^. These novel sequences that are not present in the reference genome may harbour functional genomic elements that are ethnicity-specific, and may affect gene regulations or transcriptional diversity.

To address the limitations on previously published *de novo* genome assembly, and to improve our understanding of transcriptome variations, here we sequence both DNA and RNA of a Chinese individual HX1 by single-molecule real-time (SMRT) long-read sequencing^13^. We also generate a physical map of the HX1 genome using IrysChip^14^, which is a nanopore array that detects a characteristic seven-nucleotide sequence along very long genomic segments, typically hundreds of kilobases. We perform *de novo* genome assembly to build a Chinese reference genome, using a hybrid approach that combines long-read sequencing data and IrysChip data^6^. We demonstrate a few unique applications of the HX1 assembly, including the ability to fill gaps in the human reference genome assembly GRCh38, as well as the ability to identify fine-scale structural variants. In parallel, leveraging long-read RNA sequencing, we also identify novel transcriptional elements, especially those with multiple spliced isoforms. Through the combined use of a few genomic techniques, we perform detailed characterization of the HX1 genome and demonstrate that long-read sequencing can detect functional elements in human genomes that are missed by short-read sequencing.

## Results

### *De novo* assembly of a Chinese genome

We leveraged SMRT DNA sequencing technology^13^ and sequenced genomic DNA from an anonymous Chinese individual (HX1) with normal karyotype (Supplementary Fig. 1) at 103 × genome-wide coverage (Supplementary Table 1). In total, we obtained 44.2 million ‘subreads' (a portion of the sequencing read that is informative for downstream analysis) after removing adapters and performing quality control measures (Supplementary Methods). These subreads have a mean length of 7.0 kb and a N50 length of 12.1 kb (Supplementary Table 2 and Supplementary Fig. 2), where N50 refers to the length for which the collection of all sequences of that length or longer contains at least half of the sum of the lengths of all sequences.

We modified and improved the FALCON software^15^ and performed *de novo* genome assembly on the long reads (Supplementary Methods), resulting in 5,843 contigs (N50=8.3 Mb) and a total size of 2.9 Gb. In addition, 206 Mb of ‘associated contigs', that is, alternative haplotypes, were constructed along with the primary contigs. Finally, we also performed short-read sequencing on the Illumina HiSeq X platform, with 143 × coverage of the genome (Supplementary Table 1). Short reads were used to further polish HX1 contigs and correct indel and single nucleotide variant (SNV) errors. The continuity of the contigs is substantially higher than assemblies generated from competing technologies in previous studies (Table 1), demonstrating the clear advantage of long-read sequencing in genome assembly. We note that a recently published genome using the same SMRT technology reported a contig N50 of 906 kb (ref. 6), and we believe that the almost 10-fold improvement in our study can be attributed to the improved chemistry, longer read length, enhanced assembly algorithm, as well as the much deeper sequencing depth (Supplementary Table 3).

To evaluate the completeness and accuracy of the draft HX1 assembly, we performed several analyses. First, we generated a physical map of the same DNA sample by NanoChannel-based fluidic IrysChip^14^. From the IrysChip run with 101 × whole-genome coverage (based on all sequence reads >150 kb; Supplementary Table 4 and Supplementary Fig. 3), we calculated the mapping rate of these fragments on different genome assemblies. We noted that this analysis was biased against more fragmented assemblies such as HX1, since some IrysChip reads may span two different contigs. With a highly stringent threshold suitable for human genome analysis (Supplementary Methods), the IrysChip reads yielded comparable mapping rates to GRCh38 and HX1 at 80.2% and 78.9%, respectively. Second, we aligned HX1 to GRCh38 (Supplementary Methods), and found that 97.11% of the non-N regions in GRCh38 are covered by HX1 (Supplementary Fig. 4). This ratio was at a similar level as other personal genome assemblies including YH2.0 (ref. 10) (94.99%), NA12878 (ref. 6) (97.50%) and HuRef^11^ (97.04%). Third, we evaluated consensus quality of the assemblies, by comparing them with the chromosomes in the reference genome GRCh38 using MUMmer^16^. We found that the consensus accuracy for HX1 was 99.73%, similar to YH2.0 (99.81%), NA12878 (99.73%) and HuRef (99.84%). Fourth, we also aligned the RefSeq transcripts to several genome assemblies using the NCBI Assembly Evaluation pipeline, and found that 391 out of 50,909 transcripts could not be placed on HX1 versus 455 for NA12878, 306 for YH2.0 and 22 for GRCh38 (primary assembly). The percentage of mapped coding transcripts with CDS (coding sequence) coverage ≥95% is 99.96%, 90.32%, 95.30% and 97.94% for GRCh38, YH2.0, NA12878 and HX1, respectively (Supplementary Table 5), demonstrating the low number of mis-assemblies in HX1. Similarly, the numbers of transcripts split across multiple genomic locations in GRCh38, YH2.0, NA12878 and HX1 are 11, 1,213, 1,375 and 358, respectively, demonstrating the high quality of HX1. Last but not least, short-read alignments showed that 99.42% and 99.33% of the Illumina reads can be mapped to GRCh38 and HX1, respectively, suggesting the high quality and completeness of HX1.

To generate scaffolds on the draft assembly, we applied a hybrid scaffolding approach^6^ on the IrysChip data and the HX1 draft assembly: first, we performed *de novo* assembly of the IrysChip reads, resulting in 2,346 contigs with N50 of 1.80 Mb (Supplementary Fig. 5). Next, we stitched HX1 contigs together based on information from the IrysChip assembly. Together with HX1 contigs that cannot be anchored, the N50 for the hybrid assembly improved to 22.0 Mb. We next evaluated the mis-joining error rate with a similar strategy as described in a previous study^6^, by comparing HX1 scaffolds to the reference human genome GRCh38 with 100 kb window size (Supplementary Methods). The HX1 scaffolds had a mis-joining error rate of 1.26%, similar to what was observed in YH2.0 (3.88%), NA12878 (0.55%) and HuRef (1.15%). The mis-joining error rates evaluated at smaller window sizes (10 kb) are 0.83%, 7.36%, 1.25% and 1.11% for HX1, YH2.0, NA12878 and HuRef, respectively. The final genome assembly contained 2.93 Gb primary sequences (including 39.3 Mb N-bases) and 206 Mb alternative haplotypes.

### Gap filling on the reference genome by *de novo* assembly

**Figure 1:**
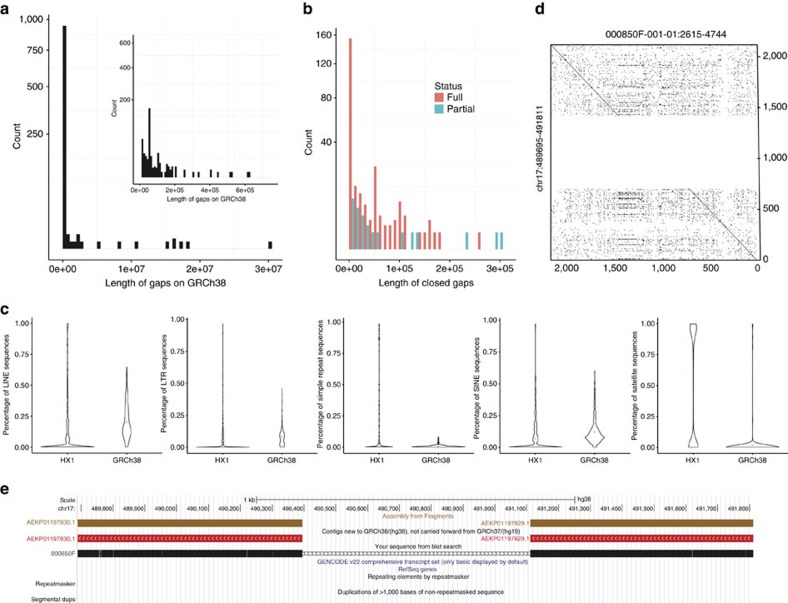
Summary of gap filling in GRCh38. (**a**) Length distribution of all gaps (stretches of ‘N' in genome sequence) in GRCh38. (**b**) Length distribution of all gaps that can be fully or partially closed. (**c**) Violin plots showing the distribution of LINE, SINE, LTR, simple repeat and satellite in closed gaps and in GRCh38. (**d**) A dotplot showing how a gap on 17p13.3 is closed by a contig in HX1. The plot shows comparison of two sequences and each dot indicates a region of close similarity between them. (**e**) Genome browser screenshot of the gap region that was closed. The gap is flanked by two contigs that are new in GRCh38 (not carried forward from GRCh37), yet an HX1 associated contig (000850F-001-01) can completely align to flanking regions, therefore filling this assembly gap and revising its length from 718 to 731 bp.

The reference human genome GRCh38 contains 966 ‘N-gaps' as stretches of Ns (see Supplementary Methods for definition), and we next assessed whether we can fill in these gaps using the *de novo* assembly. The average and median lengths of gaps in GRCh38 are 180 kb and 998 bp, respectively (Fig. 1a). One previous study by Chaisson *et al.*^17^ used SMRT sequencing to fill gaps; this study used a local assembly approach, and was able to close or extend into 31 of the 172 interstitial euchromatic gaps in GRCh38, adding 1.1-Mb sequences to the genome. Another study that used SMRT sequencing closed 28 interstitial gaps in GRCh38 with 34 kb of assembled sequences^6^. Given the availability of whole-genome *de novo* assembly, we developed a novel statistical approach called GFA (gap filling by assembly; Supplementary Methods and Supplementary Fig. 6). Interestingly, we found that 28.4% (274) of the 966 N-gaps in GRCh38 can be completely or partially filled by HX1, including 148 gaps on primary chromosomes (Supplementary Data 1). Among the 328 gaps over 10 kb, 36.0% (118) of them can be completely or partially filled. The total length of filled or shortened gaps amounts to 7.1 Mb (Fig. 1b). Compared with previous studies, gaps filled by HX1 have overlap with 48 of the 172 interstitial gaps defined by Chaisson *et al.*^17^, adding 1.8 Mb sequences to GRCh38; however, among the 48 interstitial gaps closed by us, only 10 were closed by Chaisson *et al.*, suggesting that these two gap closing methods are highly complementary. We further evaluated the repeat contents within the gaps that can be closed by us, and found that simple repeats and satellite sequences were significantly enriched within the closed gaps compared with GRCh38 (*P*<0.001; Fig. 1c). As an example, one ∼700-bp gap can be completely and confidently closed (Fig. 1d), where HX1 can be aligned to both flanking segments of the gap (Fig. 1e). In summary, together with Chaisson *et al.*, 69 out of 172 interstitial gaps in GRCh38 can be closed by long-read sequencing.

### Characterization of structural variants and novel sequences

**Figure 2:**
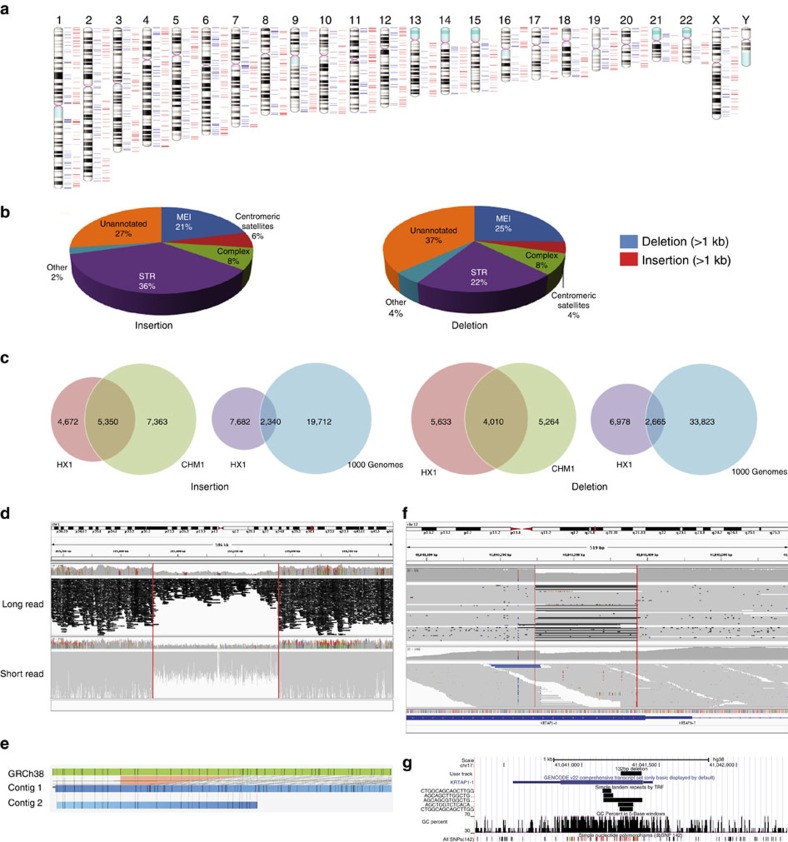
Detection of structural variants by different technologies. (**a**) Chromosome ideogram showing large-scale (>1 kb) deletions (blue) and insertions (red) identified from long-read sequencing data. (**b**) Pie chart showing the distribution of different classes of structural variants identified from long-read sequencing data. (**c**) Venn diagram showing the overlap of structural variants between HX1, CHM1 and the 1000 Genomes Project for insertions and deletions, respectively. (**d**) Integrative Genomics Viewer screenshot of the long-read (upper panel) and short-read alignment (lower panel) around an ∼200-kb deletion.(**e**) Alignment of *de novo* assembled genome map (blue) to reference genome map (green) where the ∼200-kb deletion occurs. Black vertical lines represent labels for the enzyme recognition site. Contig 2 shows identical label patterns as reference, yet contig 1 contains the deletion. (**f**) Integrative Genomics Viewer screenshot of long-read (upper panel) and short-read (lower panel) alignment around a 132-bp deletion on KRTAP1-1. This deletion is visually discernible from long-read sequencing, because the coverage is reduced and half the reads contain the deletion in alignments. However, read-depth-based method failed to detect this deletion with short read data. (**g**) Genome browser screenshot of the region surrounding the 132-bp deletion on KRTAP1-1, demonstrating the presence of simple tandem repeats and the very high GC content of the deletion

We next catalogued structural variants (SVs) in the genome of HX1, by comparing with the GRCh38 genome assembly. From long-read sequencing data, we identified 9,891 deletions and 10,284 insertions by a previously validated local assembly approach^17^ (Fig. 2a and Supplementary Methods). We classified these SVs by type and by repeat contents of the variant sequence (Table 2), and found that about half of the deletion and insertion calls are short tandem repeats or mobile element insertions (Fig. 2b). We further compared SVs in HX1 with those detected in CHM1—a haploid genome assembled by long-read sequencing and analysed by the same SV detection method^17^, as well as all SVs catalogued in the 1000 Genomes Project^9^ (Fig. 2c). Owing to the increased sensitivity of SV detection from long-read sequencing, HX1 shares substantially more SVs with the CHM1 genome, compared with all SVs catalogued in the 1000 Genomes Project. In addition, from the IrysChip physical mapping data, we identified 783 insertions and 377 deletions, using a previously validated approach^18^ (Supplementary Methods). From the short-read sequencing data, we also identified 2,403 deletions and 783 insertions (Supplementary Methods). We found that 82.8% deletions and 66.9% insertions from IrysChip overlap with SVs detected by long-read sequencing (Supplementary Fig. 7), but only 33.7% deletions and 13.8% insertions from short-read sequencing can be detected by long-read sequencing. Finally, we demonstrated an example where a 204.7-kb deletion can be visually identified by all technical platforms (Fig. 2d,e). However, a 132-bp deletion can only be detected by long-read sequencing (Fig. 2f); it is located within simple repeat regions with high (57.2%) GC contents (Fig. 2g) and shows no clear drop in coverage in Illumina data (Fig. 2f), potentially explaining its failure to be identified in Illumina data using read-depth method. However, an alternative split-read-based method^19^ with appropriate parameters was able to identify this deletion. To identify likely functional SVs specific to HX1, we filtered out calls found in segmental duplications or those shared with CHM1, and intersected the remaining calls with RefSeq exons. This left us with 35 exonic deletions and 14 exonic insertions. Among these exonic SVs, 8 deletions (23%) and 5 insertions (31%) had been previously observed in the 1000 Genomes Project, including a homozygous exonic deletion in *C1orf168* that completely removes the tenth and eleventh exons. Interestingly, this deletion was only observed as a heterozygous event in East Asian populations (allele frequency (AF)=1.1%), including 10 Han Chinese and 1 Japanese individuals, and was therefore an Asian-specific SV. In summary, long-read sequencing offers improved sensitivity in identifying SVs, especially those containing repetitive elements, and some of these SVs may contain functional genomic elements that are ethnicity-specific.

One of our primary interests lies in the identification of novel genomic elements that are absent from the reference genome assembly GRCh38. In total, we identified 12.8 Mb sequences in HX1 that were not present in GRCh38 primary scaffolds nor its alternative loci (Supplementary Methods), among which 4.1 Mb (32%) cannot be mapped to previously published Asian genome assemblies^5^,^10^, suggesting that the majority of HX1-specific sequences are likely to be found in Asian populations. To further investigate this, we re-analysed Illumina short-read sequencing data on a Chinese subject provided by the National Institute of Standards and Technology (NIST) as standard benchmarking data. Among the 907 million raw reads, we achieved a mapping rate of 99.56% to GRCh38; among unmapped reads, we found that 7.8% can be mapped to the novel sequence in HX1, confirming that Asian-specific sequence elements were present but were missed from GRCh38. We next performed variant calling on the NIST genome. In the regions shared between HX1 and GRCh38, we identified 3,157,818 SNVs on HX1 but 3,852,118 SNVs on GRCh38, suggesting that Asian-specific reference genome might reduce the number of called SNVs. However, HX1 might be less appropriate for indel calling due to higher indel error rate of SMRT long reads. Among the SNVs called on HX1, 30,713 (∼1%) resided within the novel sequences of HX1. We further examined the contents within the novel sequences in HX1 (Supplementary Table 6), and found that microsatellites are significantly enriched in the novel sequences compared with genome-wide average (75.5% versus 2.1%, *P*<10^−5^). Similarly, simple repeats are also significantly over-represented (13.0% versus 0.8%, *P*<10^−5^). Therefore, long-read sequencing is especially effective in capturing highly repetitive regions in the genome.

Several other previously published studies reported novel sequence elements from Asians, so we performed comparative analysis. For example, Li *et al.*^12^ found 7,330 novel sequences (4.9 Mb) absent from GRCh37. Re-analysis of their data showed that 3,440 (3.7 Mb) sequences can be aligned to GRCh38, yet 4,716 (4.4 Mb) can be aligned to HX1. Among these sequences, 3,154 (3.5 Mb) can be aligned to both GRCh38 and HX1. Furthermore, a recent study identified seven novel genes in an Asian genome assembly by comparing with the GRCh37 assembly and examining the transcriptome^5^. We identified all seven genes in HX1, but also found that they were present in GRCh38, indicating the improvements in coverage of GRCh38 over GRCh37 (Supplementary Fig. 8). To assess whether regulatory functional elements exist in novel sequences in HX1, we analysed raw sequencing data on five markers (CTCF, DNase I hypersensitivity, H3K4me1, H3K4me3 and H3K27ac) on the lymphoblastoid cell line GM12878 from the ENCODE project (Supplementary Table 7)^20^. Among all the reads that cannot be mapped to GRCh38, 1.1% can be mapped to HX1 (Supplementary Table 8) and we performed peak-calling on each marker. We also performed RNA-Seq on HX1 using Illumina short-read sequencing, and found that genes closest to these peaks within the 500-kb flanking region tend to have higher expression levels than all other genes in the RNA-Seq data (Supplementary Fig. 9). In summary, we demonstrated that long-read sequencing can identify potentially functional pieces in genomes that evade detection by short-read sequencing.

### Characterization of transcriptome variation

**Figure 3:**
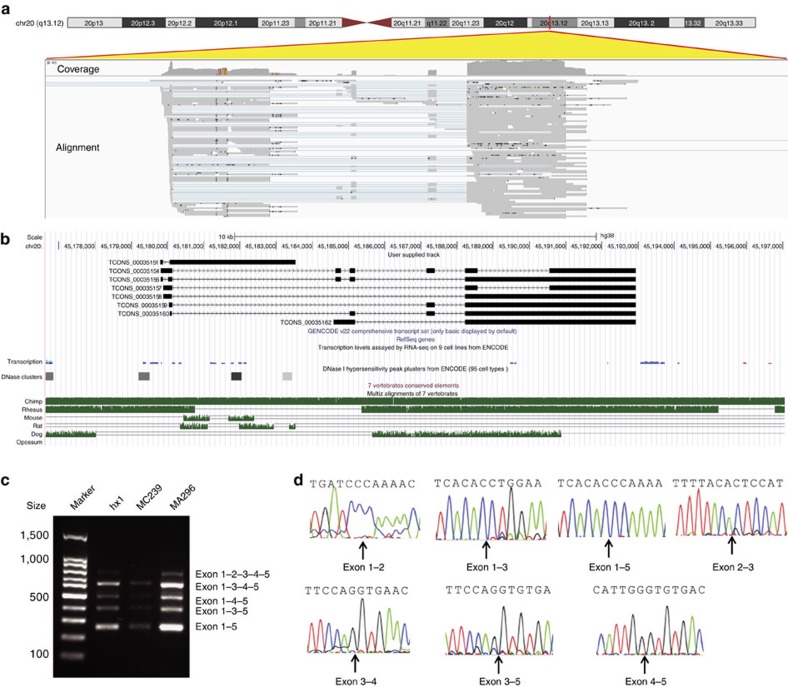
Novel gene inferred from Iso-Seq long-read RNA sequencing. (**a**) Integrative Genomics Viewer on alignment files generated from Iso-Seq. Over 100 long reads can be mapped to this locus on chr20q13.12 in the GRCh38 assembly. (**b**) UCSC Genome Browser screenshot on the predicted transcript models. The transcripts are not detected in RNA-Seq data on nine cell lines in ENCODE. This gene is conserved in primates but not in other vertebrate species, and is not in segmental duplication regions or simple repeat regions. (**c**) PCR validation of the transcript TCONS_0035154 by a primer pair that targeted exons 1 and 5. Several PCR products with different sizes can be detected, representing different isoforms. MC239 is a Caucasian sample and MA296 is an East Asian sample. (**d**) Sanger sequencing confirmed the splicing events predicted by the Iso-Seq data.

To evaluate transcriptional diversity and identify novel transcripts, we performed long-read RNA sequencing (Iso-Seq) on RNA samples extracted from whole blood. Iso-Seq uses a Circular Consensus Sequence protocol, where a given transcript is made into a circular molecule and sequenced multiple times through the circle. Given that transcripts vary greatly in size, we generated four different libraries: 1–2 kb; 2–3 kb; 3–5 kb and 5 kb+, each with 10–16 SMRT cells with a total count of 50 cells (Supplementary Table 9 and Supplementary Figs 10 and 11). For the four libraries, on average each transcript had 11.2, 8.4, 6.9 and 5.2 passes, respectively. We also used short-read RNA-Seq data to correct errors in Iso-Seq data (Supplementary Table 10 and Supplementary Figs 12 and 13). From Iso-Seq data, we predicted 58,383 high-quality consensus isoforms at 30,006 loci. Focusing on consensus isoforms that are highly expressed, we identified 57 isoforms at 42 loci that do not overlap with any GENCODE transcript (Supplementary Table 11). We experimentally validated several spliced transcripts, that is, those with more than two predicted exons (Supplementary Table 12 and Supplementary Figs 14 and 15). For example, from Iso-Seq alignments (Fig. 3a), we identified a novel transcribed element with at least five exons and six isoforms (Fig. 3b), and validated the presence of predicted splicing events by PCR (Fig. 3c) and Sanger sequencing (Fig. 3d). This transcribed element is conserved among primates, but absent from other species (Fig. 3b), and it has not been detected by short-read RNA-Seq on all nine cell lines from ENCODE (Fig. 3b). Similarly, from long-read sequencing data, we identified another two novel genes on 11q13.4 (five exons) and 14q32.2 (four exons), which evades detection by short-read RNA-Seq (Supplementary Fig. 16), and validated their presence by Sanger sequencing of cDNA (Supplementary Figs 17 and 18).

### Functional analysis of variants with clinical relevance

**Figure 4:**
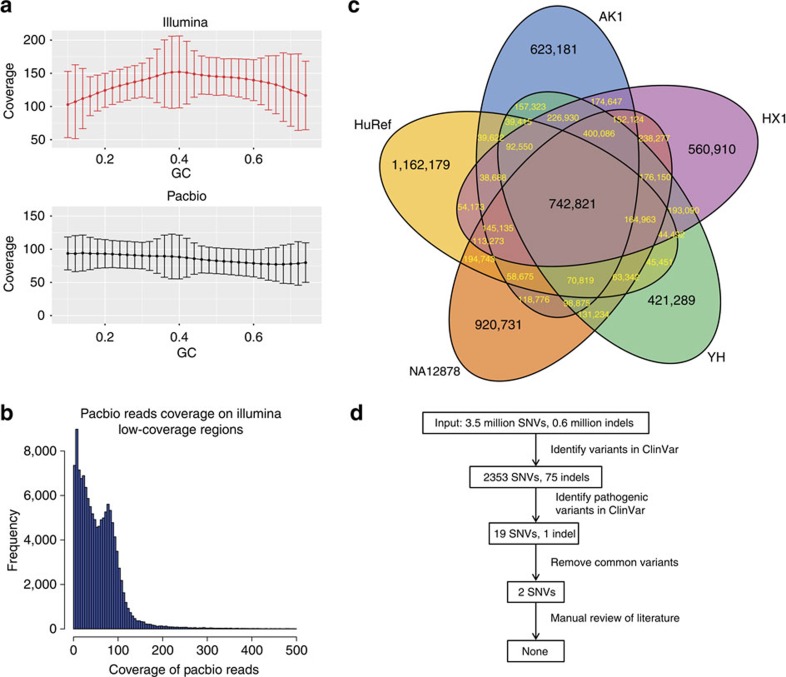
Functional annotation and analysis of the genomic variants in HX1. (**a**) Average coverage versus GC contents for 100-bp windows in Illumina data and PacBio data, respectively. The mean and s.d. values are shown. (**b**) Distribution of PacBio coverage for regions that have ≤5 × coverage in Illumina data. (**c**) Shared SNVs discovered in HX1, AK1, HuRef, NA12878 and YH. (**d**) Variant reduction pipeline to identify pathogenic variant; although 20 were annotated as ‘pathogenic' in ClinVar, careful analysis failed to support any one.

One major utility of personal genome sequencing is to identify disease-related genetic variants. We identified 3,518,309 SNVs and 625,690 indels from HX1 by comparing Illumina reads with GRCh38 (Supplementary Figs 19–21). Among them, 223,883 SNVs and 197,402 indels had minor allele frequency ≤0.01 in the 1000 Genomes Project^21^, and 74,143 SNVs and 62,260 indels were not documented in dbSNP^22^ version 142. Among these novel variants, 372 SNVs and 50 indels resided in exonic regions (Supplementary Figs 22 and 23). Despite the high coverage (143 ×), we noted that the coverage in Illumina data was far more susceptible to GC biases than PacBio data (Fig. 4a), and that PacBio reads generally had high coverage in a large proportion of regions that are poorly covered (≤5 ×) in Illumina data (Fig. 4b). We next compared these SNVs from HX1 with those from several previously published personal genomes, including AK1 (ref. 23), YH, HuRef and NA12878 (Fig. 4c). As expected, we found that HX1 shared more variants with the two Asian genomes (1,462,387), compared with the two Caucasian genomes (1,166,192). Moreover, the number of unique SNVs in HX1 (560,910) lied between two other Asian genomes, AK1 (623,181) and YH (421,289), yet smaller than two Caucasian genomes, HuRef (1,162,179) and NA12878 (920,731). Overall, 742,821 SNVs (21.1%) were shared among HX1 and four other personal genomes.

To identify genetic variants that may be of clinical significance, we annotated HX1 variants against the ClinVar database^24^. A total of 2,432 variants (2,357 SNVs and 75 indels) in HX1 were documented in ClinVar^24^, including 20 variants that were classified as ‘pathogenic'. However, a simple allele frequency filter with manual examination showed that 18 of these 20 ‘pathogenic' variants had minor allele frequency >1% in the 1000 Genomes Project, and were unlikely to be highly penetrant disease causal variants (Fig. 4d). The remaining two variants included one upstream variant at the *MSMB* locus that was annotated as pathogenic for hereditary prostate cancer, as well as one stop-gain variant within *DUOXA2* that was annotated as pathogenic for thyroid dyshormonogenesis. Manual review of the literature cited in the two ClinVar records^25^,^26^ indicated that both of them represented erroneous database records. Therefore, no known pathogenic variants truly exist in HX1. This analysis highlights the need for extreme caution in interpreting ‘pathogenic' variants documented in variant databases, and suggests that frequency filter as well as manual review are necessary to tease out false positives. With the continuous improvements of ClinVar, the addition of evidence codes for clinical interpretation, and the expansion of public allele frequency databases, this problem is expected to be alleviated in the future.

## Discussion

In the current study, we generated one of the first near-complete *de novo* genome assemblies for a Chinese individual. The contig and scaffold N50 values of the assembly were substantially higher than previous studies on *de novo* human genome assembly, implicating the unique advantage of long-read sequencing in assembling complex genomes such as the human genome. In addition, our study also identified a number of previously unreported functional genomic elements, some of which can be transcribed. Therefore, long-read RNA sequencing may complement conventional short-read RNA sequencing to capture the complete landscape of the human transcriptome.

There are several current limitations to use long-read sequencing to generate *de novo* assemblies and analyse personal genomes. First, although the read length is substantially higher than short-read sequencing, it is still at the scale of tens of kilobases due to the technological limitations of the current sequencing platform. Some highly complex genomic regions may still not be adequately assayed or assembled, especially when sequencing coverage is low. With the current development of a number of nanopore-based long-read sequencing platforms, this problem may be alleviated by technological innovations. Second, some gaps in the reference genome are long and are surrounded by segmental duplications or other highly repetitive sequences^27^; therefore, they may not be filled by our long read assembly. For example, 24 of the 966 gaps are longer than 300 kb based on current estimation, and none of them can be closed by our method. Third, compared with the Illumina platform that enabled $1000 genomes, PacBio long-read sequencing is still relatively cost-prohibitive to be applied to personal genomes at large scale. With the continued decline in sequencing cost and the improvement in sequencing throughput per flow cell, this problem may be reduced in the future. Fourth, due to the much higher error rates (especially for indels) of PacBio long-read sequencing, variant detection will not be reliable at low sequencing coverage, so analysis of genetic mutations in personal genomes still needs to rely on more accurate short-read sequencing data. Finally, in the current study, to demonstrate the use of reference-free *de novo* assembly, we used the NanoChannel arrays for scaffolding the contigs from the genome assembly, which resulted in ∼3-fold improvements in N50 values. However, we acknowledge that we could alternatively use the reference human genome GRCh38 for generating much longer scaffolds.

In summary, while short-read-based alignment and variant calling based on reference genome remain a common practice to assay personal genomes, *de novo* assembly by long-read sequencing may reveal novel and complementary biological insights. Furthermore, long-read RNA sequencing may identify novel transcripts that can be missed by short-read RNA sequencing. Improved understanding and better characterization of genome functional variation may require the use of a range of genomic technologies on diverse human populations^28^.

## Methods

### Generation of sequence data

Freshly drawn blood samples were obtained from an anonymous healthy Chinese adult male (HX1) with normal karyotype, using protocols approved by the Institutional Review Board of Jinan University. HX1 provided written consent for public release of genomic data. For long-read DNA sequencing, high-molecular-weight DNA was extracted from isolated lymphocytes using Phenol–Chloroform method and sequenced by the PacBio sequencer RS II, with the P6-C4 sequencing reagent. For long-read RNA sequencing, total RNA was extracted from blood using TRIzol extraction reagent and subjected to the IsoSeq protocol with four library sizes (1–2 kb, 2–3 kb, 3–5 kb and 5 kb+), and sequenced on the PacBio sequencer RS II. For short-read DNA sequencing, genomic DNA was subject to Illumina TruSeq Nano library preparation protocol, and 151-bp paired-end reads were generated by Illumina HiSeq X sequencer. For short-read RNA sequencing, RNA samples were subject to the TruSeq mRNA Library Kit and sequenced on Illumina HiSeq2500 sequencer. For BioNano physical mapping, DNA extracted from freshly drawn whole blood were subject to manufacturer-recommended protocols for library preparation and optical scanning, with the default nicking enzyme NT.BspQI.

### *De novo* genome assembly

Long-read *de novo* genome assembly was performed with an enhanced version of FALCON software, which improved its performance in an NFS-based computing cluster. The source code is available on GitHub (https://github.com/WGLab/EnhancedFALCON). Since we sequenced a diploid human genome, alternative haplotypes may exist in certain regions with high variability or large structural variants. As a result, associated contigs are constructed by FALCON. BioNano *de novo* genome assembly was performed by the IrysView software on a computational cluster, with manufacturer-recommended parameters and with molecular length threshold of 150 kb. Quality assessment of BioNano data used a stringent parameter of ‘-T 1e-9' suitable for human genome and a 10% subsampling strategy. Hybrid scaffolding of the PacBio-based assembly and BioNano-based assembly was performed by the hybrid scaffolding module packaged with the IrysView software, with details given in Supplementary Methods. Short-read polishing was performed with BWA-MEM^29^, FreeBayes^30^ and custom python scripts. Consensus quality evaluation was done by MUMmer^16^. Mis-joining rate was calculated by custom python scripts on BWA-MEM alignments.

### Gap filling

We developed a GFA procedure for closing gaps in the reference genome. Any region consisting of continuous runs of N in the target assembly (GRCh38) is defined as a gap in our method. After merging gaps that are ≤500 bp apart, flanking sequences upstream and downstream of the gaps were mapped to the source assembly (HX1). If two anchor sequences for the same gap can both be aligned, they will be examined to remove discordant pairs, which include those alignments with inconsistent orientation, on different contigs, or overlapping with each other. If only one anchor can be aligned, then the anchor will be extended into the gap region wherever possible. The source code for GFA has been deposited to github (https://github.com/WGLab/uniline). Detailed statistical formulation was given in Supplementary Methods. Briefly, for a gap with length *L*_0_ and a predicted gap with length *L*_g_, we showed that the probability of observing a gap with difference *d*=*L*_g_−*L*_0_ or less extreme is


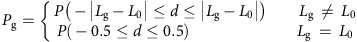


Gap closing quality score is then calculated by summing up Phred-scaled *P*_g_ and mapping quality score (*P*_a_) assuming independence. The model permits ∼10% of flexibility at a threshold of 30 and does not penalize harshly when *L*_g_ deviates two to three times from 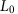.

### Transcriptome analysis and validation

We first performed isoform-level clustering using the RS_IsoSeq protocol within the SMRTPortal software. This protocol essentially performs isoform-level clustering (ICE) and polishes the results with Quiver. The output from ICE algorithm contains consensus sequences from full-length reads. The Quiver polished output is classified into either ‘low QV' or ‘high QV'. Our analysis focused on the high-QC consensus isoform clusters, where ‘Quiver high QV' is currently set with an expected consensus accuracy of 99%. Once we obtained the high-quality consensus clusters, we further aligned them to the GRCh38 reference genome using the GMAP^31^ algorithm. To improve Iso-Seq read alignment, we further performed error correction of all original Iso-Seq reads using LSC, following similar steps in its original publication^32^. LSC is an algorithm designed for improving PacBio long-read accuracy by short-read alignment from Illumina RNA-Seq. Alignment and analysis of short-read RNA sequencing data was performed by the TopHat^33^ software and Cufflinks^34^ software, respectively. The fragments per kilobase of transcript per million mapped reads (FPKM) measure was used for quantification of gene expression in the short-read sequencing data. Comparison of transcript models was performed by the CuffCompare software within the Cufflinks package. We validated several novel transcripts with more than two predicted exons, by designing pairs of PCR primers that are located in two adjacent exons, and performed PCR reactions on the cDNA samples. The gel bands were cut and DNA was recovered by Qiagen QIAquick kit (Valencia, CA, USA), and used for Sanger sequencing.

### Detection and analysis of genome variation

Detection of structural variation on short-read sequencing data, long-read sequencing data and BioNano mapping data were performed by the CNVNator^35^, local assembly^17^ and IrysView software, respectively. SNP and indel calling were performed by the SeqMule^36^ software, which integrates BWA-MEM^29^ for alignment and GATK^37^/FreeBayes^30^/SAMtools^38^ for variant calling. Comparative analysis of genome assembly was performed by the MUMmer^16^ and the LASTZ^39^, together with custom scripts. Annotation and functional analysis of genetic variants were performed with the ANNOVAR^40^ software, using a variety of built-in databases to infer gene-level functional annotations^41^, the allele frequencies in the 1000 Genomes Project^21^, the presence/absence in dbSNP^22^ (version 142) and ClinVar^24^ (version 20160302), and the allele frequency in several public databases. Statistical analysis and graph generation were performed using R (http://www.R-project.org) and custom Perl/Python scripts.

### Data availability

Data generated during the study are available from the authors. All raw short-read and long-read sequencing data have been deposited in the NCBI short read archive under study PRJNA301527. The genome assembly and related results can be accessed at http://hx1.wglab.org/.

## Additional information

**How to cite this article:** Shi, L. *et al.* Long-read sequencing and *de novo* assembly of a Chinese genome. *Nat. Commun.* 7:12065 doi: 10.1038/ncomms12065 (2016).

## Supplementary Material

Supplementary Information Supplementary Figures 1-23, Supplementary Tables 1-12, Supplementary Methods, Supplementary References

Supplementary Data 1 A list of gaps in GRCh38 that can be closed or extended by HX1.

## Figures and Tables

**Table 1:**
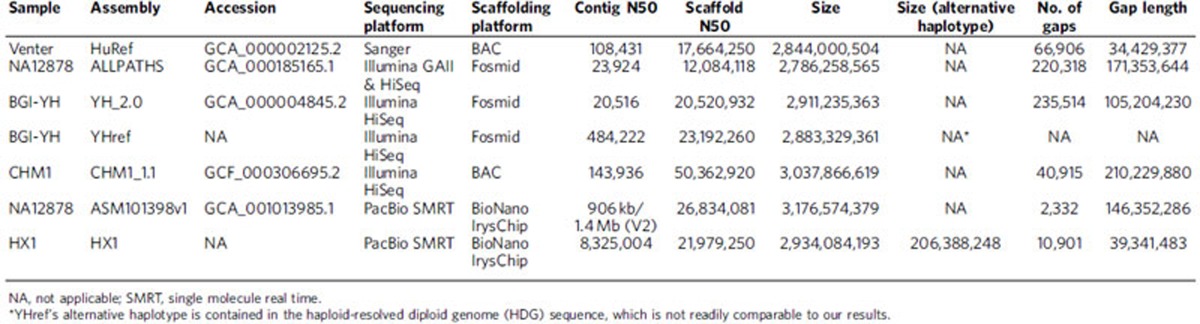
Comparison with previously published de novo assembly on personal genomes.

**Table 2:** Structural variants detected in HX1 in comparison to GRCh38.

**Repeat category**	**Insertion**			**Deletion**			**Ins/del**	
	**Number**	**Mean length**	**Total bases**	**Number**	**Mean length**	**Total bases**	**Total events**	**Total bases**
MEI	2,172	600	1,302,834	2,499	504	1,258,260	0.87	1.04
L1	175	1,121	196,227	207	552	114,177	0.85	1.72
L1HS	117	2,622	306,826	146	2,648	386,599	0.80	0.79
L1P	152	770	116,964	214	579	123,871	0.71	0.94
Mosaic Alu	187	496	92,842	141	360	50,724	1.33	1.83
AluS	110	219	24,144	127	180	22,819	0.87	1.06
Alu_STR	93	961	89,407	63	439	27,662	1.48	3.23
AluY	865	286	247,698	1,153	284	327,077	0.75	0.76
SVA	292	511	149,172	227	618	140,327	1.29	1.06
HERV	23	318	7,320	31	263	8,141	0.74	0.90
LTR	92	533	49,047	108	256	27,665	0.85	1.77
MER	66	351	23,187	82	356	29,198	0.80	0.79
Centromeric Satellites	608	561	340,995	436	501	218,243	1.39	1.56
ALR	484	612	296,077	387	551	213,177	1.25	1.39
HSAT	124	362	44,918	49	103	5,066	2.53	8.87
Complex	822	3,065	2,519,672	766	3,075	2,355,090	1.07	1.07
STR	3,655	332	1,212,704	2,130	176	374,176	1.72	3.24
Other	256	269	68,982	431	243	104,795	0.59	0.66
Unannotated	2,771	210	580,924	3,629	180	654,225	0.76	0.89
Total	10,284	586	6,026,111	9,891	502	4,964,789	1.04	1.21

Alu_STR, Alu element associated with STR; complex, multiple repeat elements are found; MEI, mobile element insertion; STR, short tandem repeat. SVA, short interspersed nuclear elements/variable number tandem repeat/Alu; HERV, human endogenous retrovirus; LTR, long tandem repeat; MER, medium reiteration frequency repeats; ALR, α-satellite repeat; HSAT, human satellite sequence.

All events are larger than 50 bp.
