# Supplementary material for: Long-read sequencing and de novo assembly of a Chinese genome
Source: Nat Commun. 2016 Jun 30;7:12065. doi: 10.1038/ncomms12065 (PMC4931320; doi:10.1038/ncomms12065)
Supplement: Supplementary Information — Supplementary Figures 1-23, Supplementary Tables 1-12, Supplementary Methods, Supplementary References [file ncomms12065-s1.pdf]

Supplementary Figures

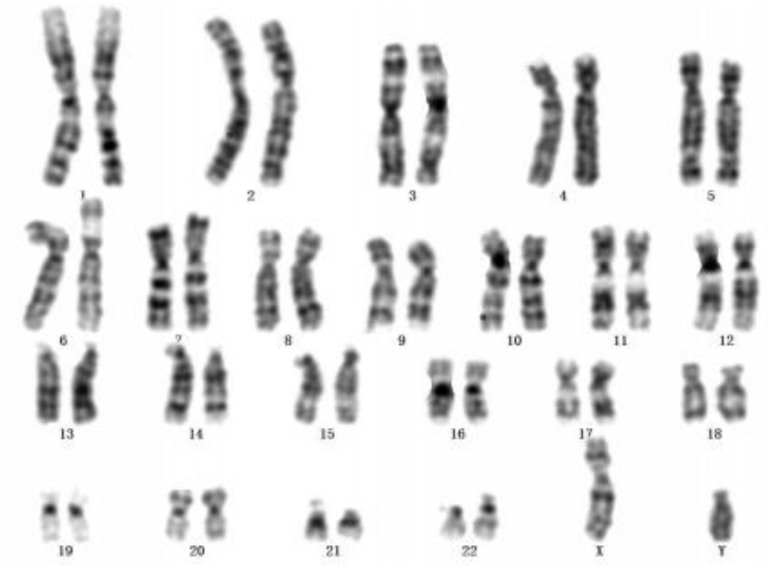

Supplementary Figure 1. Karyotype analysis did not reveal large-scale chromosomal abnormality in HX1.

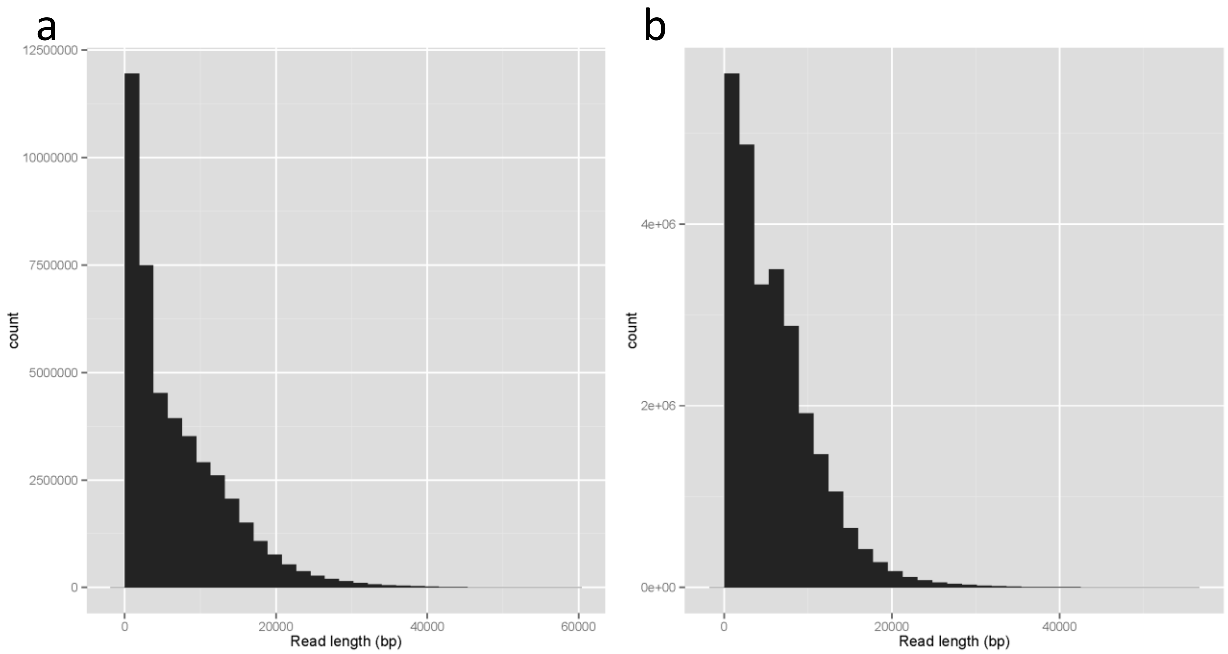

Supplementary Figure 2. Length distribution of subreads and error-corrected subreads. (A) Read length distribution of filtered subreads. (B) Read length distribution of error-corrected subreads. Filtered subreads are used in error-correction by DALIGNER in Falcon. Error-corrected subreads are used for de novo assembly.

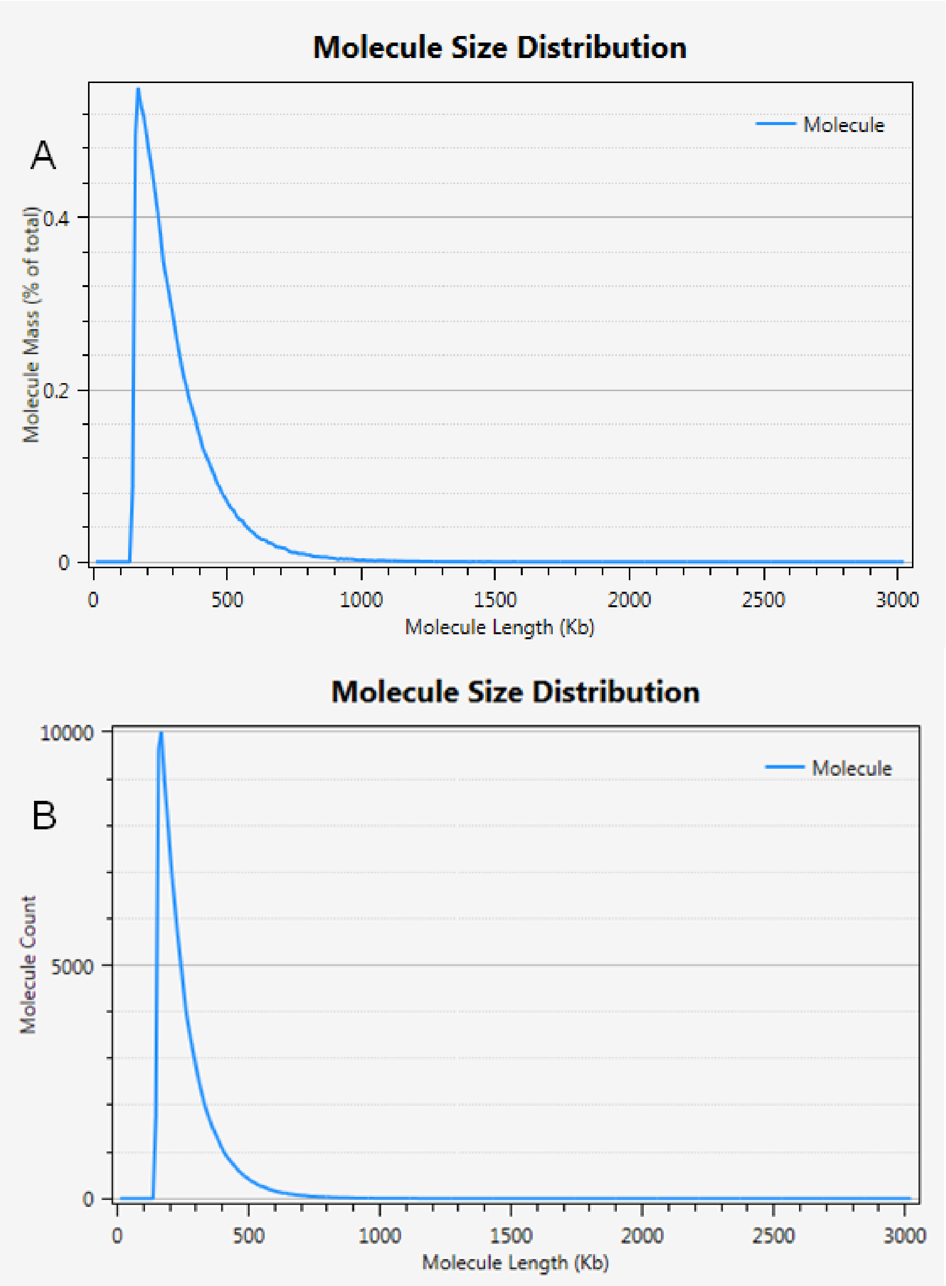

Supplementary Figure 3. Summary of the IrysChip analysis. (A) Distribution of the molecule mass versus the size of the molecules in filtered data. (B) Distribution of the molecule count versus the size of the molecules in filtered data.

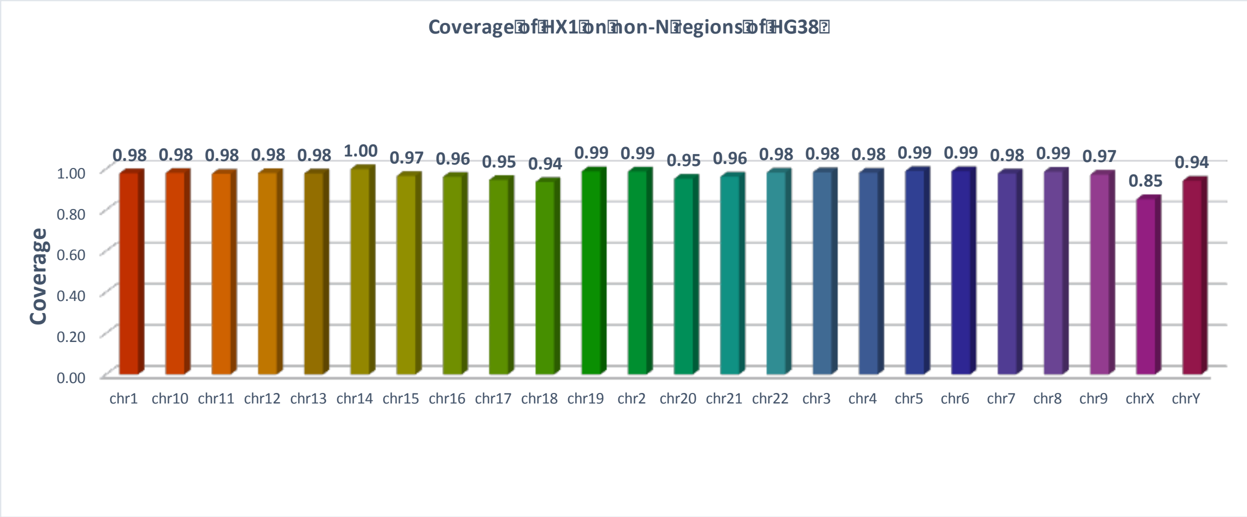

Supplementary Figure 4. Non-N coverage of GRCh38 by HX1 assembly for each chromosome.

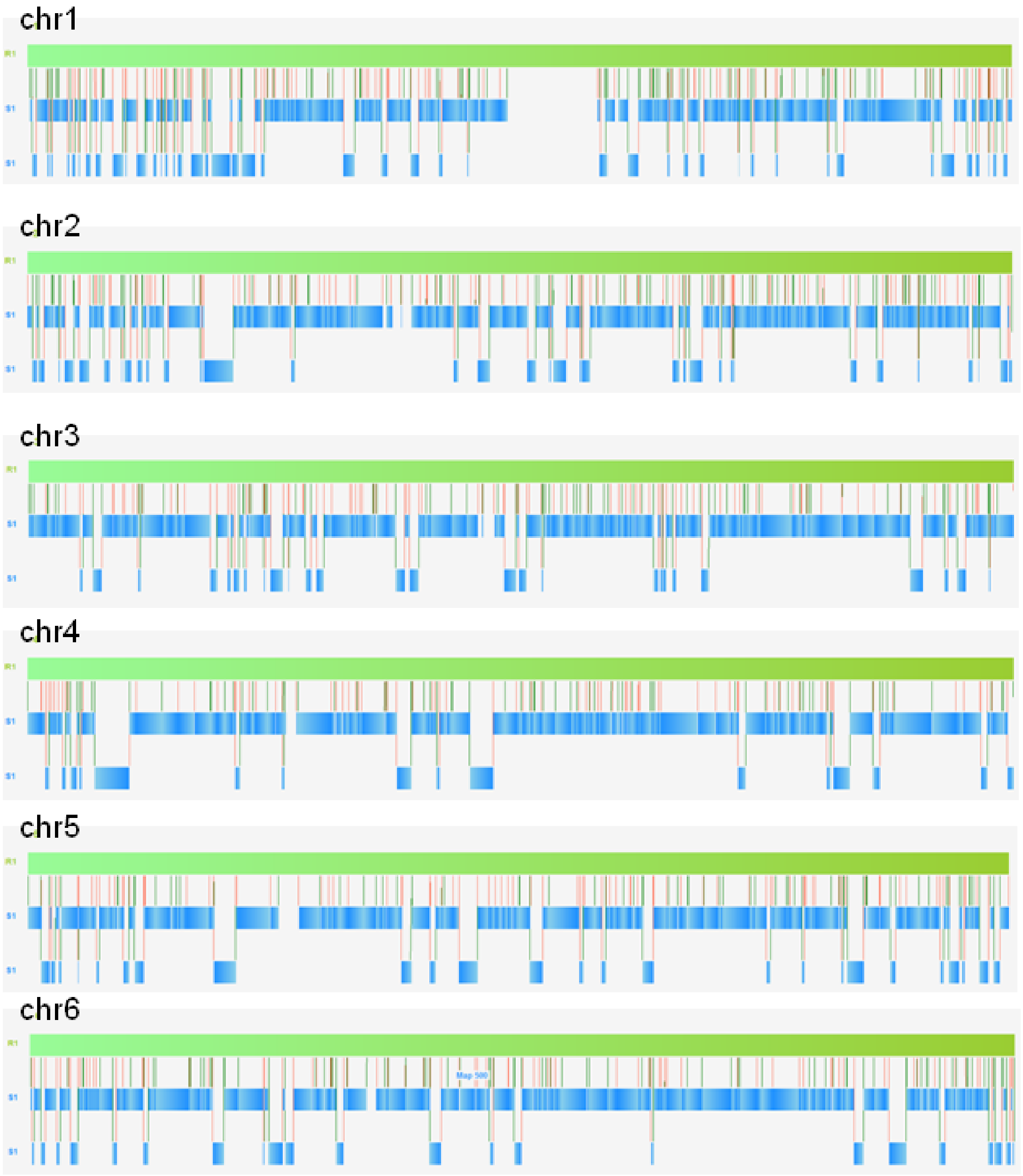
chr1
chr2
chr3
chr4
chr5
chr6

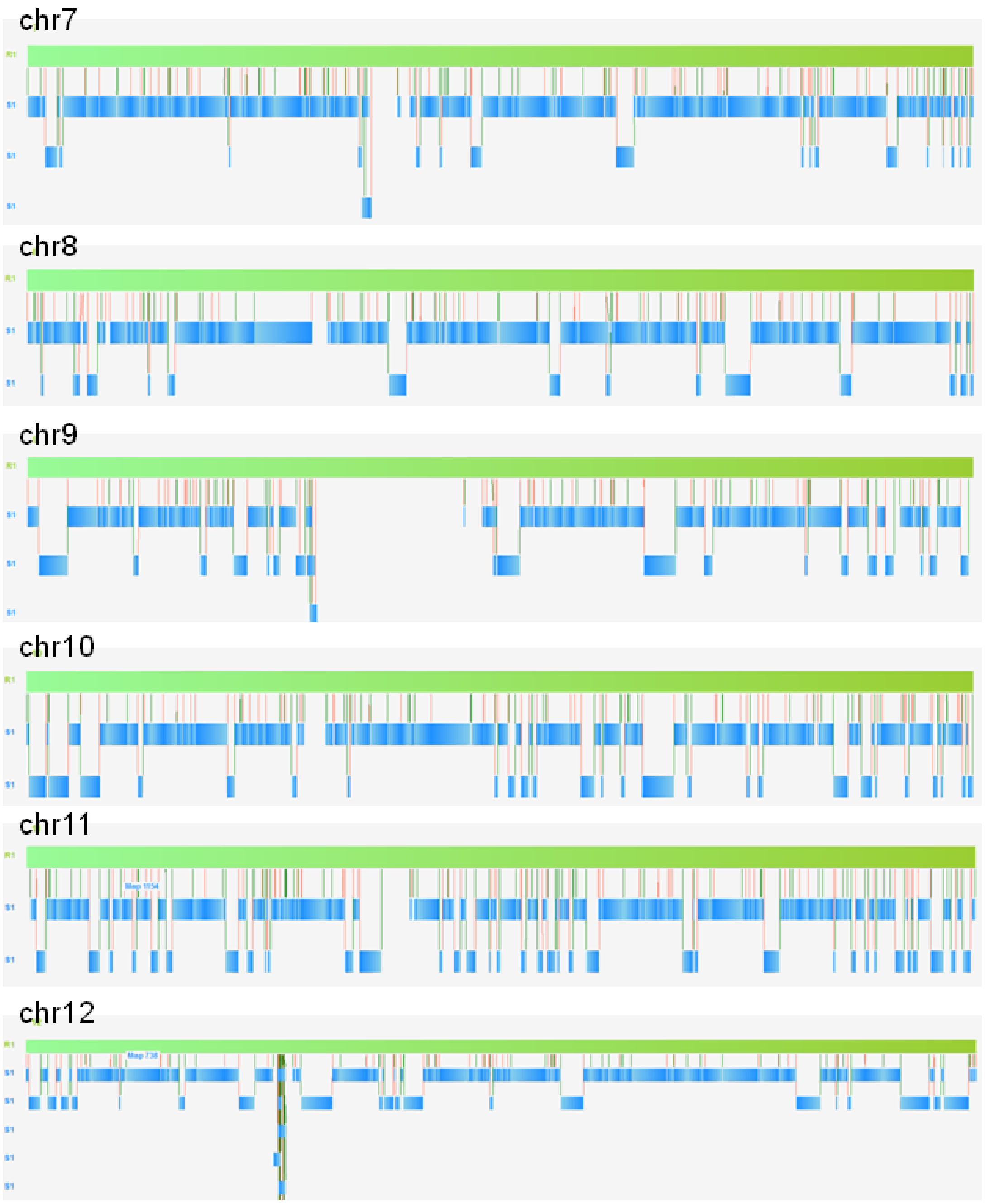
chr7
chr8
chr9
chr10
chr11
chr12

chr13

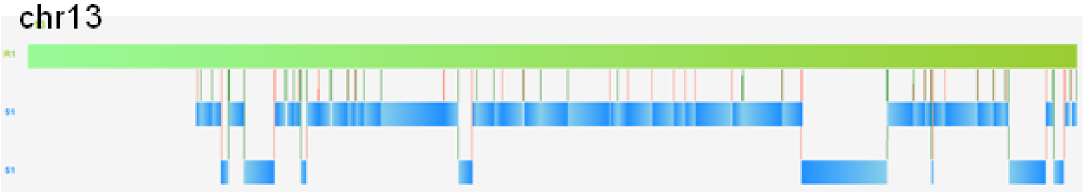

chr14

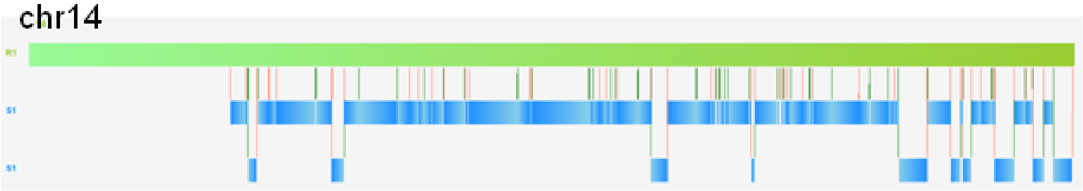

chr15

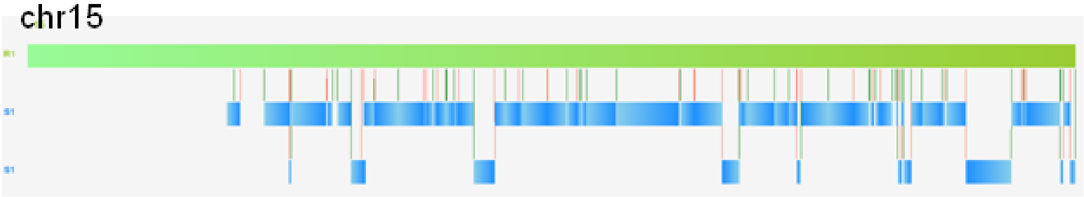

chr16

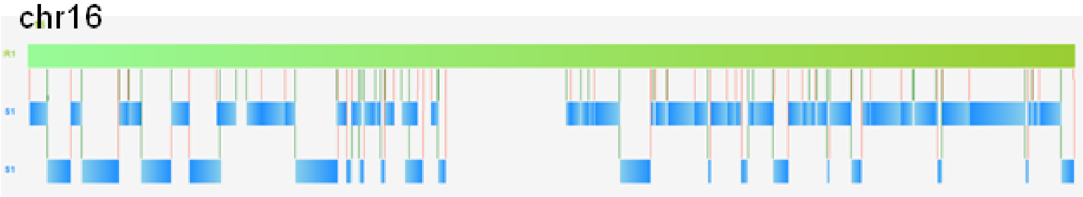

chr17

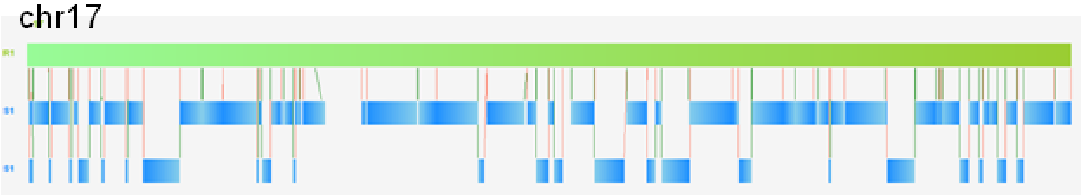

chr18

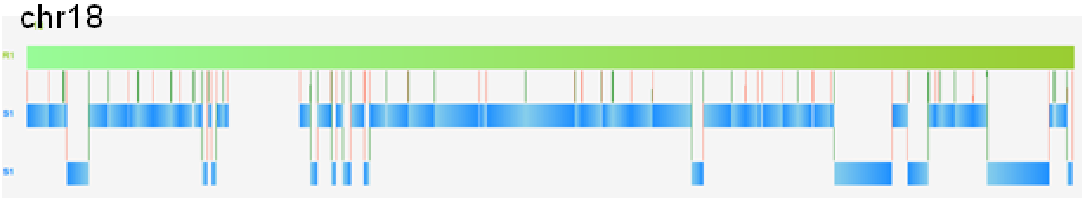

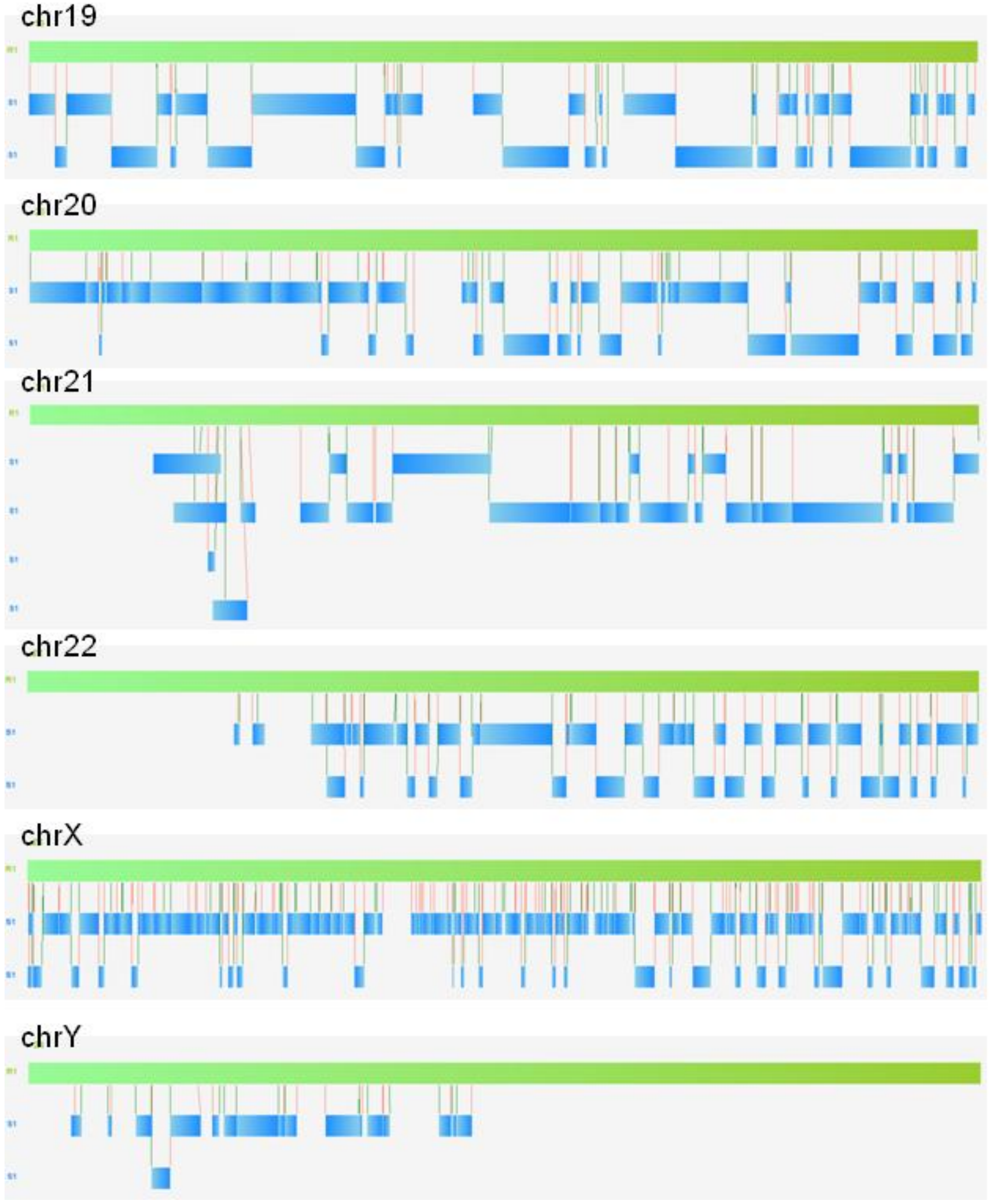

Supplementary Figure 5. De novo BioNano assembly alignment to chr1-22, ChrX and chrY in GRCh38. Chromosome number is on top left corner. Reference genome is in green, while HX1 assembly is in blue.

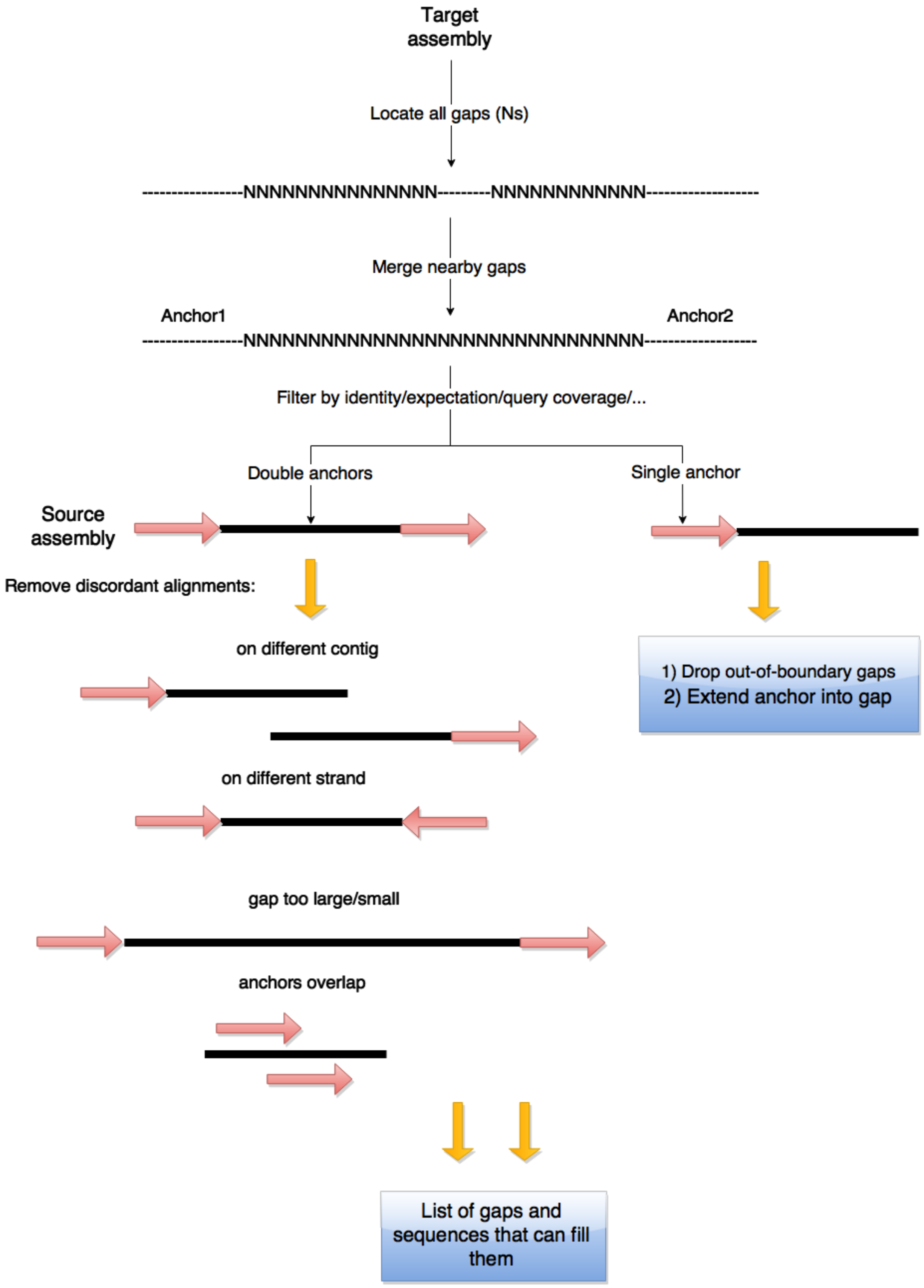

Supplementary Figure 6. Illustration of the Gap Filling by Assembly (GFA) procedure.

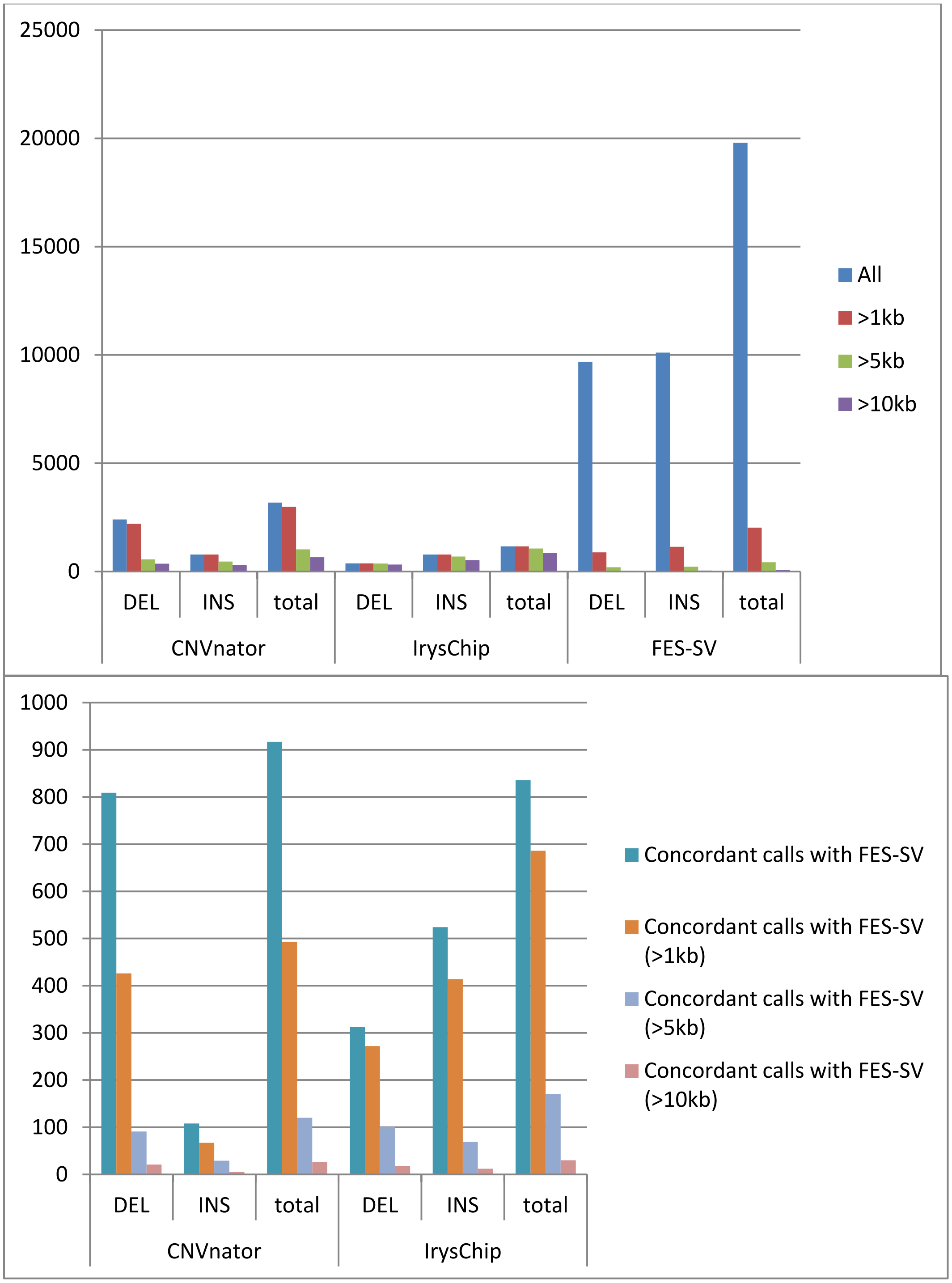

Supplementary Figure 7. Structural variation calls across different technologies. (A) The number of CNV calls generated by CNVnator (Illumain short-read sequencing), IrysChip (BioNano physical mapping), FES-SV (PacBio long-read sequencing) at different size thresholds. (B) The concordance of FES-SV calls with the other two technologies.

A

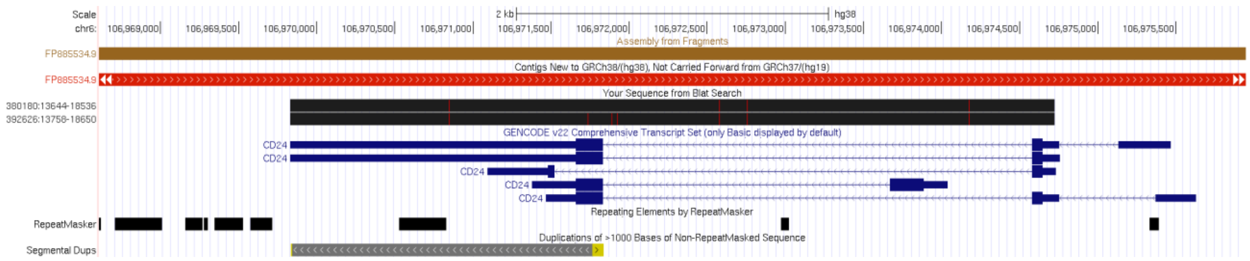
Scale
chr6:
2 kb
hg38
Assembly from Fragments
FP885534.9
Contigs New to GRCh38/(hg38), Not Carried Forward from GRCh37/(hg19)
Your Sequence from Blat Search
GENCODE v22 Comprehensive Transcript Set (only Basic displayed by default)
CD24
Repeating Elements by RepeatMasker
RepeatMasker
Duplications of >1000 Bases of Non-RepeatMasked Sequence
Segmental Dups

B

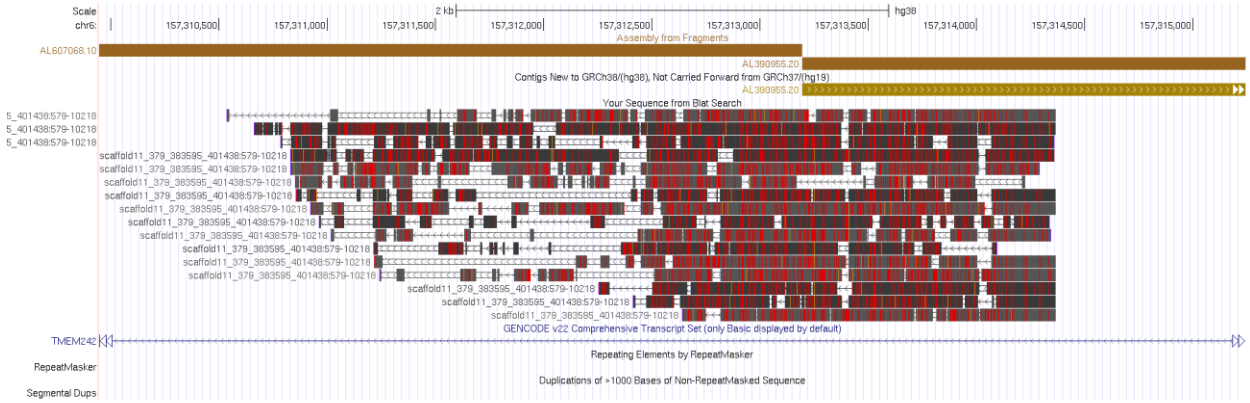
Scale
chr6:
2 kb
hg38
Assembly from Fragments
AL607068.10
AL390955.20
Contigs New to GRCh38/(hg38), Not Carried Forward from GRCh37/(hg19)
Your Sequence from Blat Search
GENCODE v22 Comprehensive Transcript Set (only Basic displayed by default)
TMEM242
Repeating Elements by RepeatMasker
RepeatMasker
Duplications of >1000 Bases of Non-RepeatMasked Sequence
Segmental Dups

C

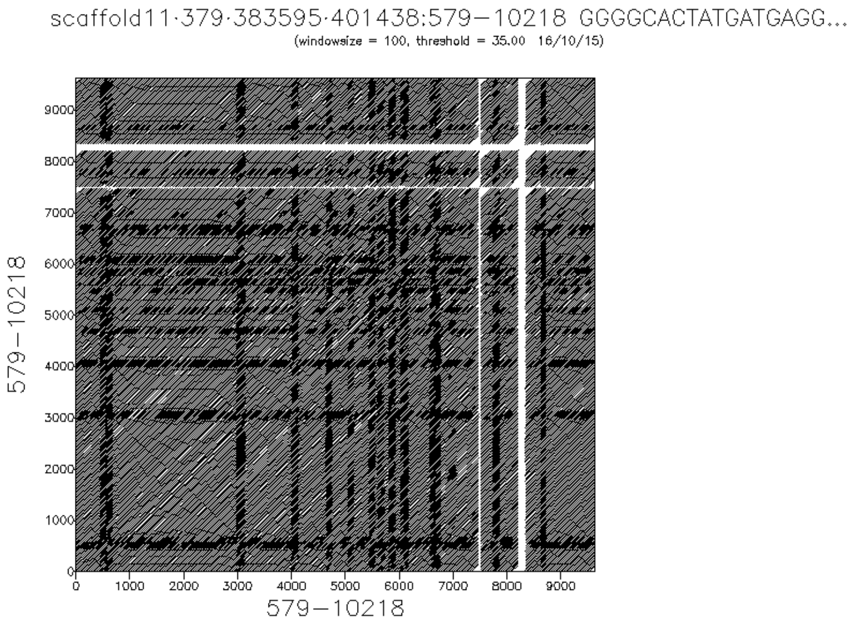
scaffold11·379·383595·401438:579−10218 GGGGCACTATGATGAGG...
(windowsize = 100, threshold = 35.00 16/10/15)
579−10218
579−10218

D

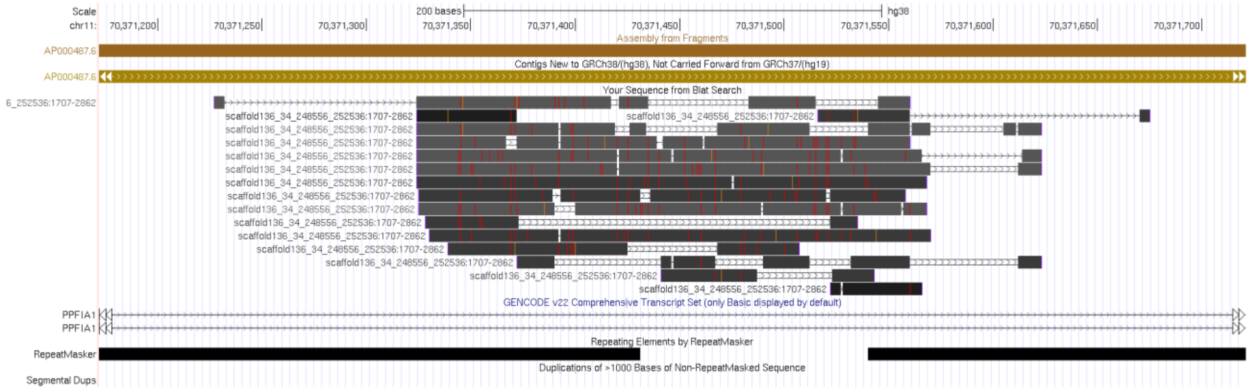
Scale
chr11:
200 bases
hg38
70,371,200
70,371,250
70,371,300
70,371,350
70,371,400
70,371,450
70,371,500
70,371,550
70,371,600
70,371,650
70,371,700
Assembly from Fragments
AP000487.6
Contigs New to GRCh38/(hg38), Not Carried Forward from GRCh37/(hg19)
AP000487.6
Your Sequence from Blat Search
6_252536:1707-2862
scaffold136_34_248556_252536:1707-2862
GENCODE v22 Comprehensive Transcript Set (only Basic displayed by default)
PPFIA1
PPFIA1
Repeating Elements by RepeatMasker
RepeatMasker
Duplications of >1000 Bases of Non-RepeatMasked Sequence
Segmental Dups

E

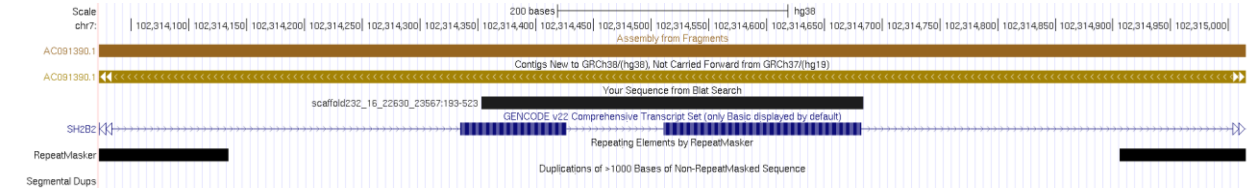
Scale
chr7:
200 bases
hg38
102,314,100
102,314,150
102,314,200
102,314,250
102,314,300
102,314,350
102,314,400
102,314,450
102,314,500
102,314,550
102,314,600
102,314,650
102,314,700
102,314,750
102,314,800
102,314,850
102,314,900
102,314,950
102,315,000
Assembly from Fragments
AC091390.1
Contigs New to GRCh38/(hg38), Not Carried Forward from GRCh37/(hg19)
AC091390.1
Your Sequence from Blat Search
scaffold232_16_22630_23567:193-523
GENCODE v22 Comprehensive Transcript Set (only Basic displayed by default)
SH2B2
Repeating Elements by RepeatMasker
RepeatMasker
Duplications of >1000 Bases of Non-RepeatMasked Sequence
Segmental Dups

F

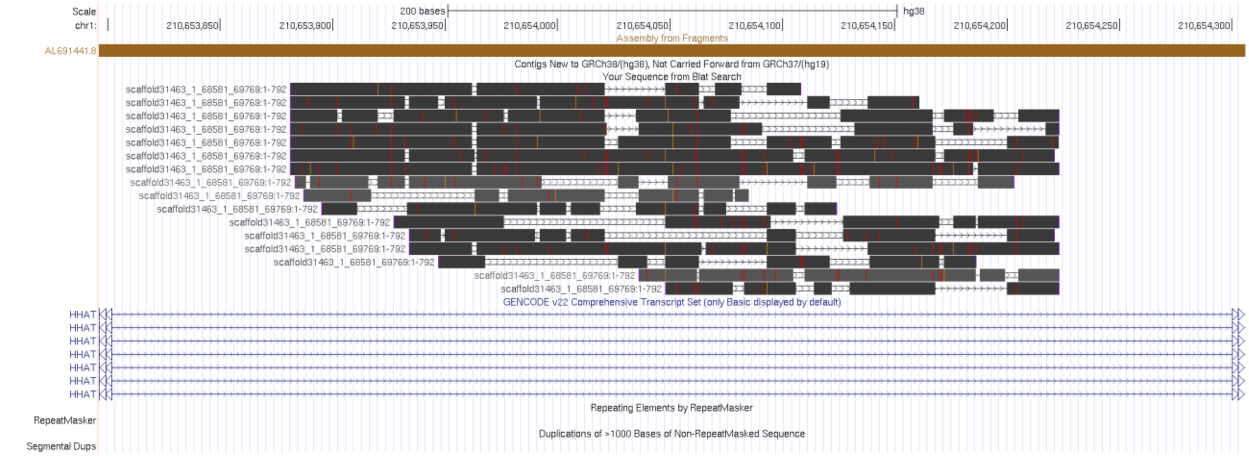
Scale
chr1:
200 bases
hg38
210,653,850
210,653,900
210,653,950
210,654,000
210,654,050
210,654,100
210,654,150
210,654,200
210,654,250
210,654,300
Assembly from Fragments
AL691441.8
Contigs New to GRCh38/(hg38), Not Carried Forward from GRCh37/(hg19)
Your Sequence from Blat Search
scaffold31463_1_68581_69769:1-792
GENCODE v22 Comprehensive Transcript Set (only Basic displayed by default)
HHAT
Repeating Elements by RepeatMasker
RepeatMasker
Duplications of >1000 Bases of Non-RepeatMasked Sequence
Segmental Dups

G

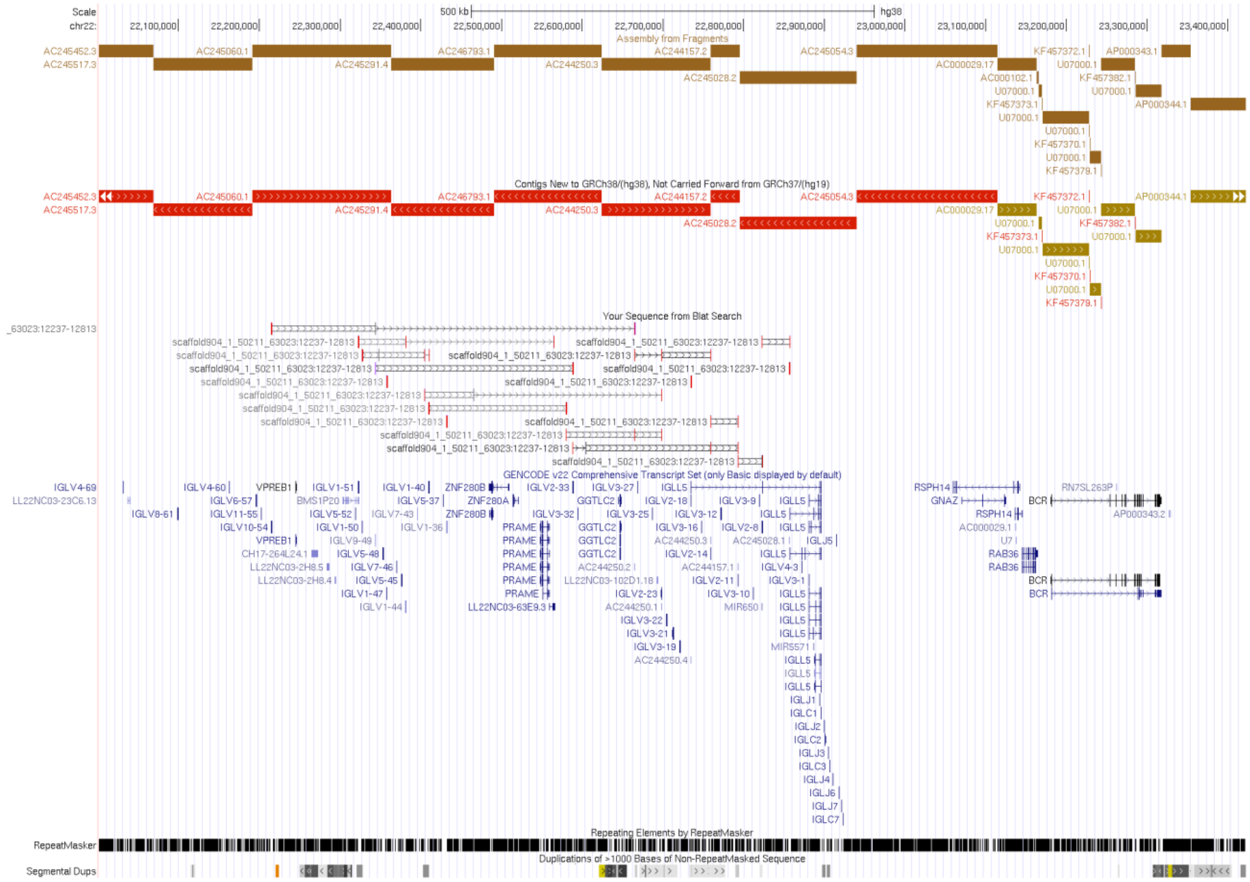

Supplementary Figure 8. Detailed analysis of a list of 7 previously published novel genes in Asian genomes. We extracted the FASTA sequences for these 7 genes based on originally reported scaffold coordinates in the original genome assembly, and then performed BLAT search against GRCh38. All the seven sequences can be mapped to GRCh38 completely. (A) >scaffold11_377_336296_392626:13758-18650 and >scaffold11_378_324399_380180:13644-18536: These two reported novel sequences are identical and both mapped completely to the CDC24 gene. (B) >scaffold11_379_383595_401438:579-10218: This reported novel sequence contains several largely identical long repeat, and all repeats map to the intron of TMEM242 gene. (C) To further illustrate this, we generated dotplot (comparison of two sequences and each dot indicates a region of close similarity between them) and demonstrated the presence of many repetitive regions. (D) >scaffold136_34_248556_252536:1707-2862: This reported novel sequence contains several largely identical long repeat, and all repeats map to the intron of PPFIA1 gene. (E) >scaffold232_16_22630_23567:193-523: This reported novel sequence maps completely to the SH2B2 gene. (F) >scaffold31463_1_68581_69769:1-792 and>scaffold904_1_50211_63023:12237-12813: This novel sequence has multiple parts that all map to the immunoglobulin region, suggesting that it merely reflects the different combinations of V(D)J recombination of immunoglobulin genes (G) This reported novel sequence contains several largely identical long repeat, and all repeats map to the intron of HHAT gene.

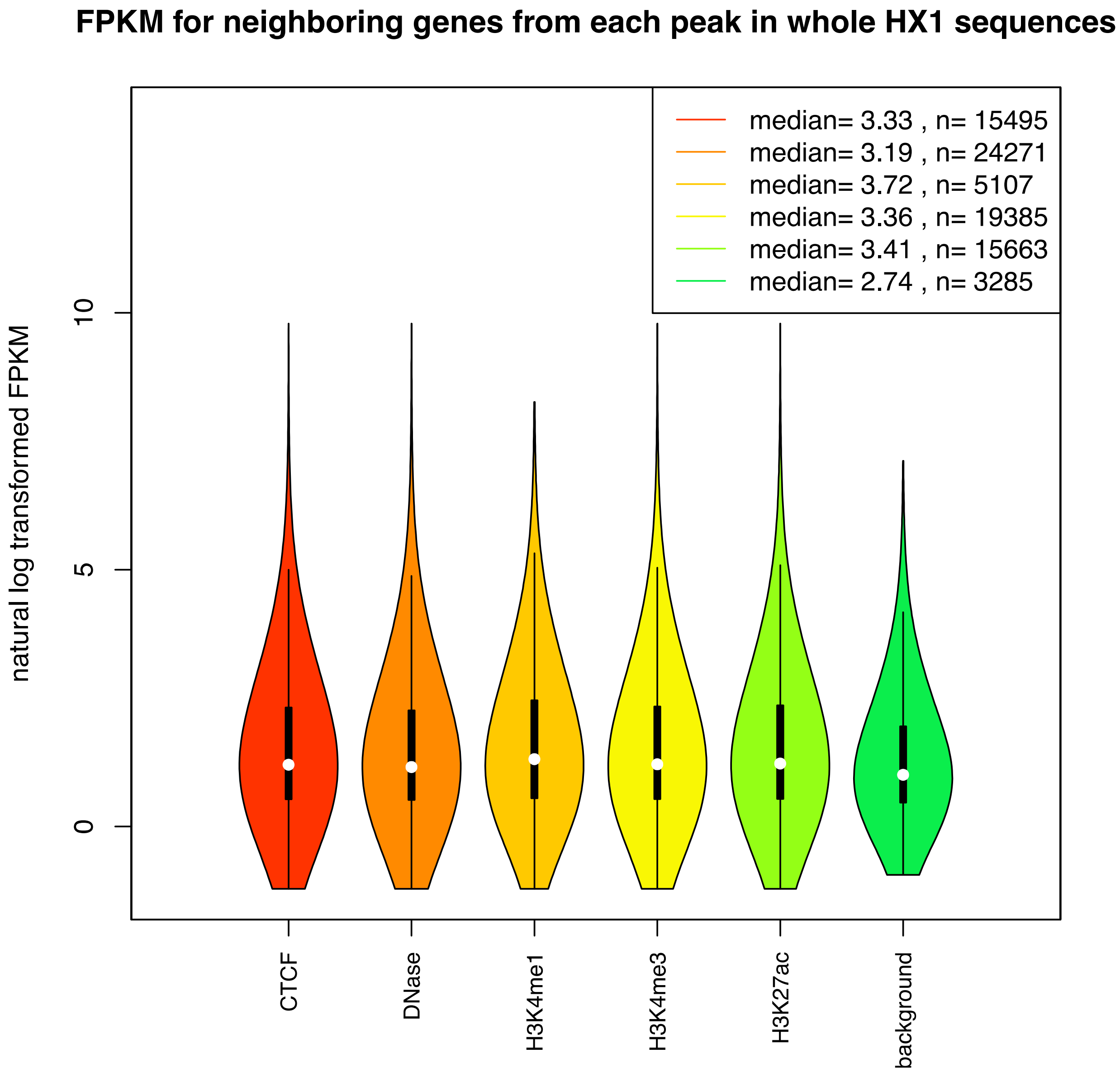

Supplementary Figure 9. Distribution of FKPM values for genes flanking each of the five markers (CTCF, DNase, H3K4me1, H3K4me3, H3K27ac), compared to background expression. For each peak, the nearest gene with 500kb flanking region is analyzed.

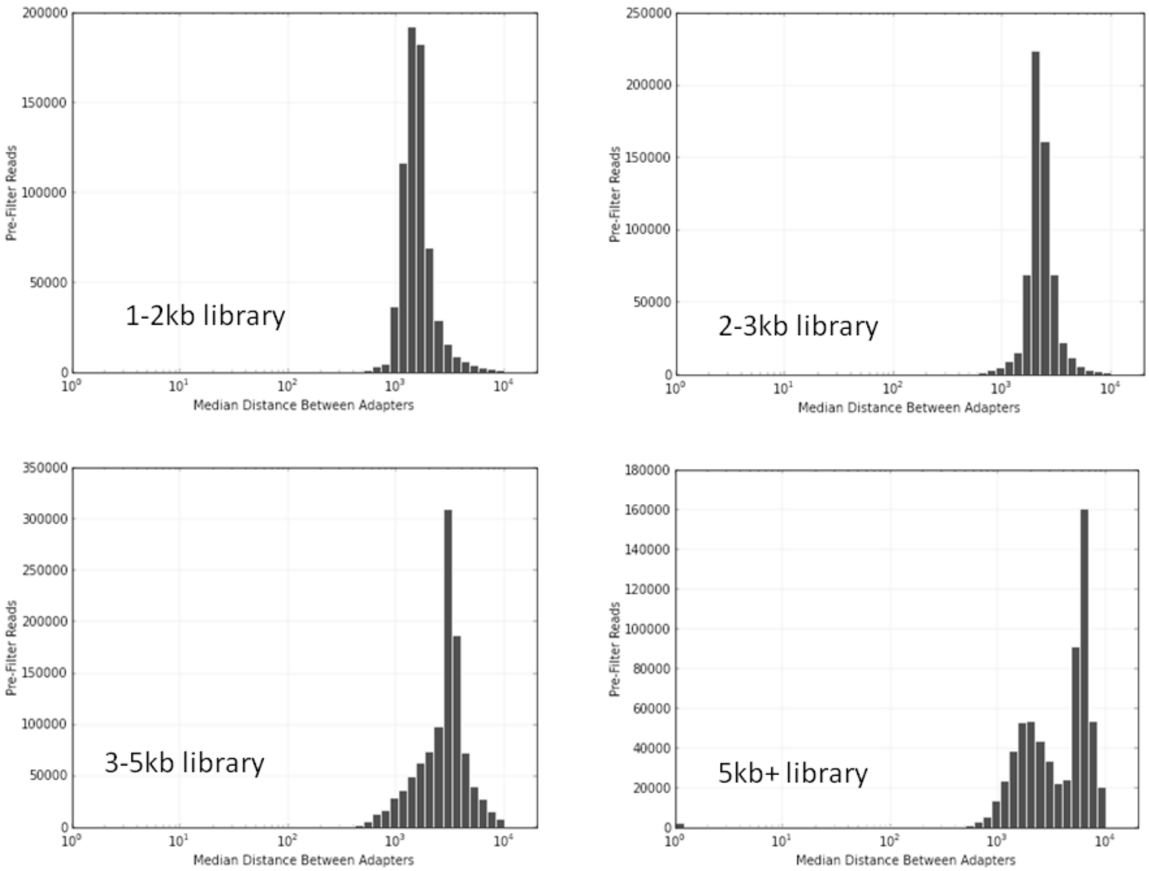

Supplementary Figure 10. The distribution of observed insert length in four Iso-Seq libraries.

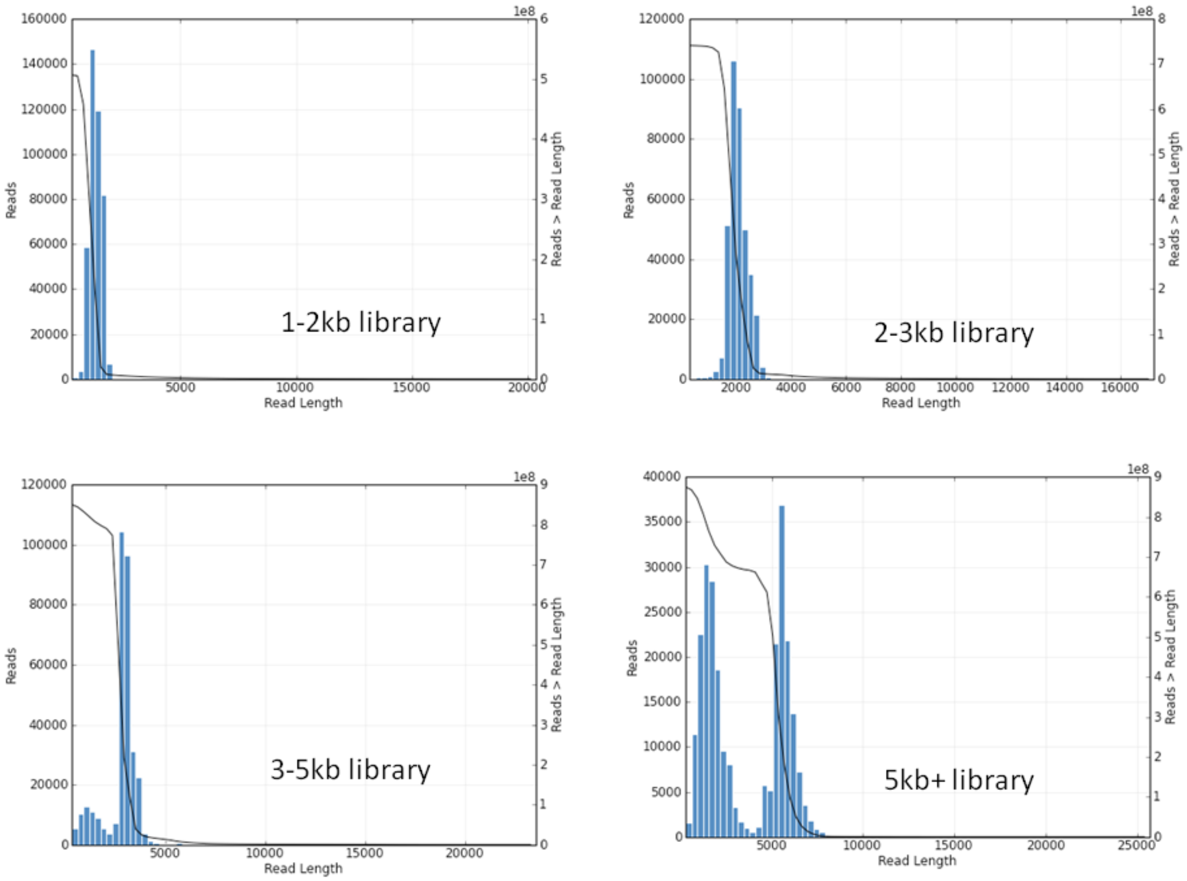

Supplementary Figure 11. The distribution of full-length non-chimeric reads in four libraries. Full-length reads are identified by examining existence of 5' primer, polyA tail and 3' primer in reads. Artificial chimeras are detected with incidence of identifiable cDNA primer in the middle of a read.

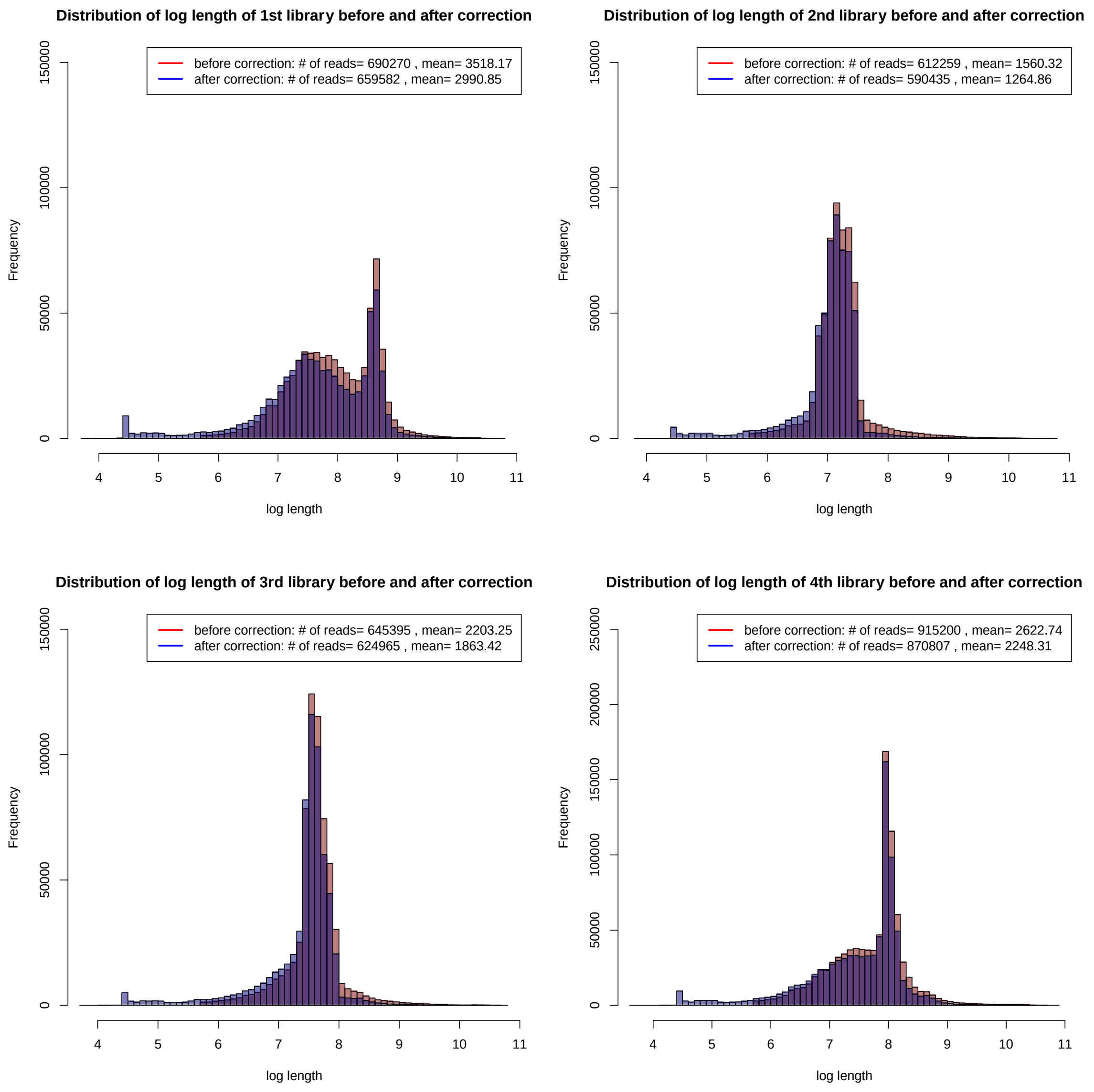

Supplementary Figure 12. Distribution of log transformed length of Iso-Seq reads from four libraries before and after error correction.

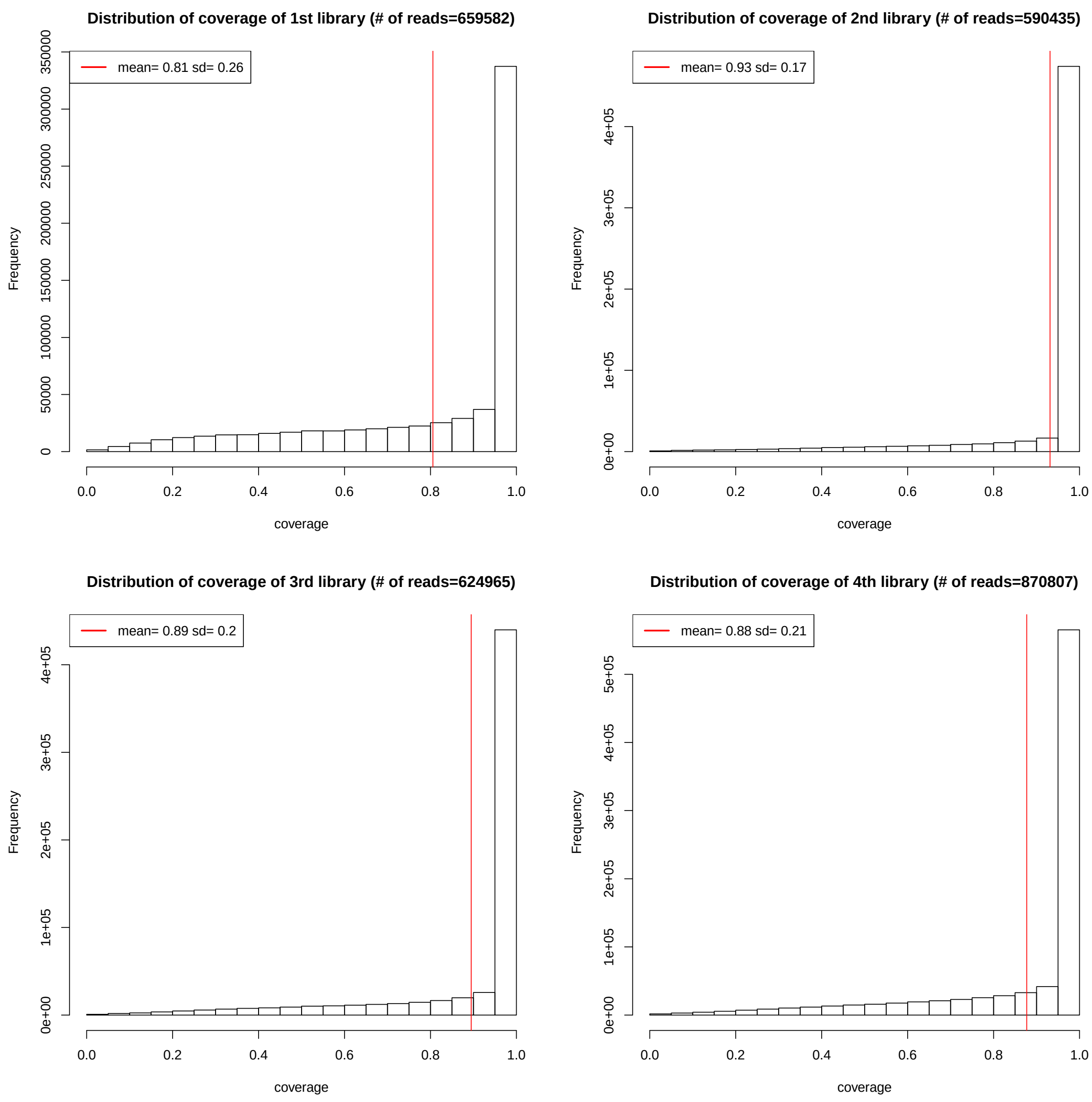

Supplementary Figure 13. Distribution of coverage of Iso-Seq reads from four libraries before and after error correction.

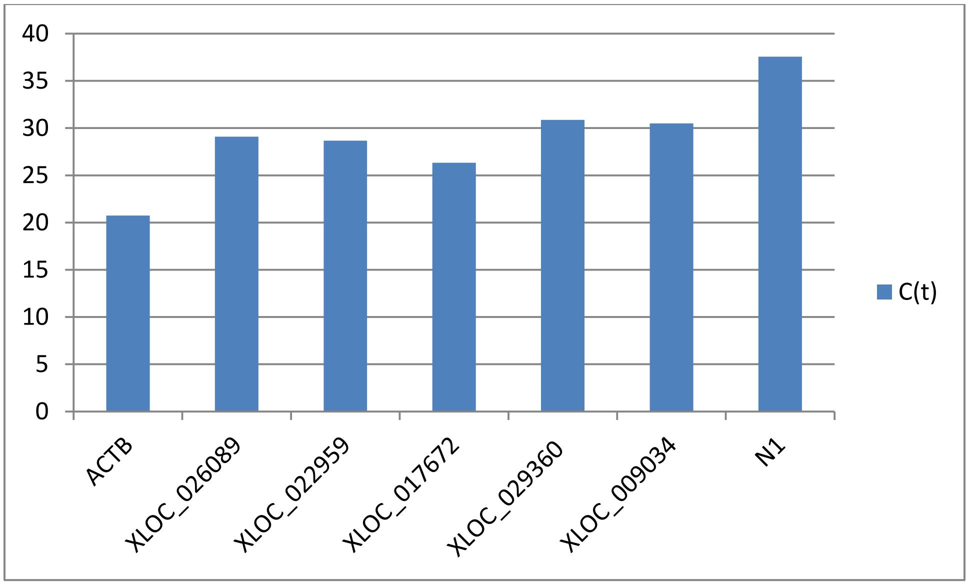

Supplementary Figure 14. Quantification of expression levels for novel transcripts against ACTB using C(t)-based quantitative PCR. N1 is the negative control that has no detectable expression levels in the Iso-Seq data.

A

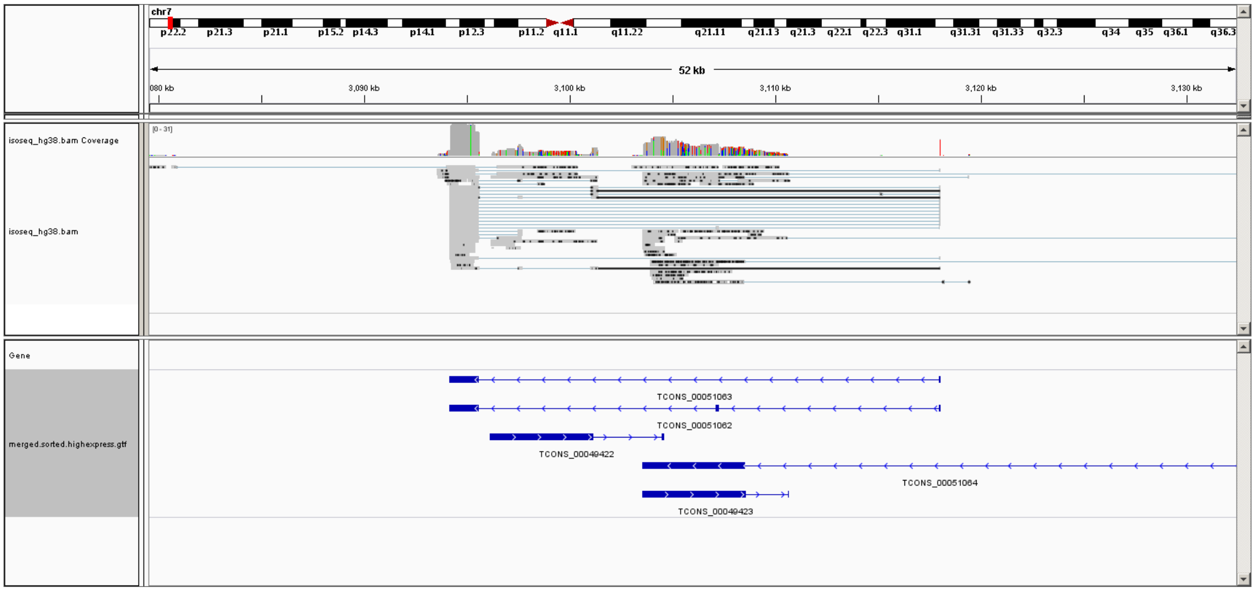
chr7
52 kb
isoseq_hg38.bam Coverage
isoseq_hg38.bam
Gene
merged.sorted.highexpress.gtf
TCONS_00051063
TCONS_00051062
TCONS_00049422
TCONS_00051064
TCONS_00049423

B

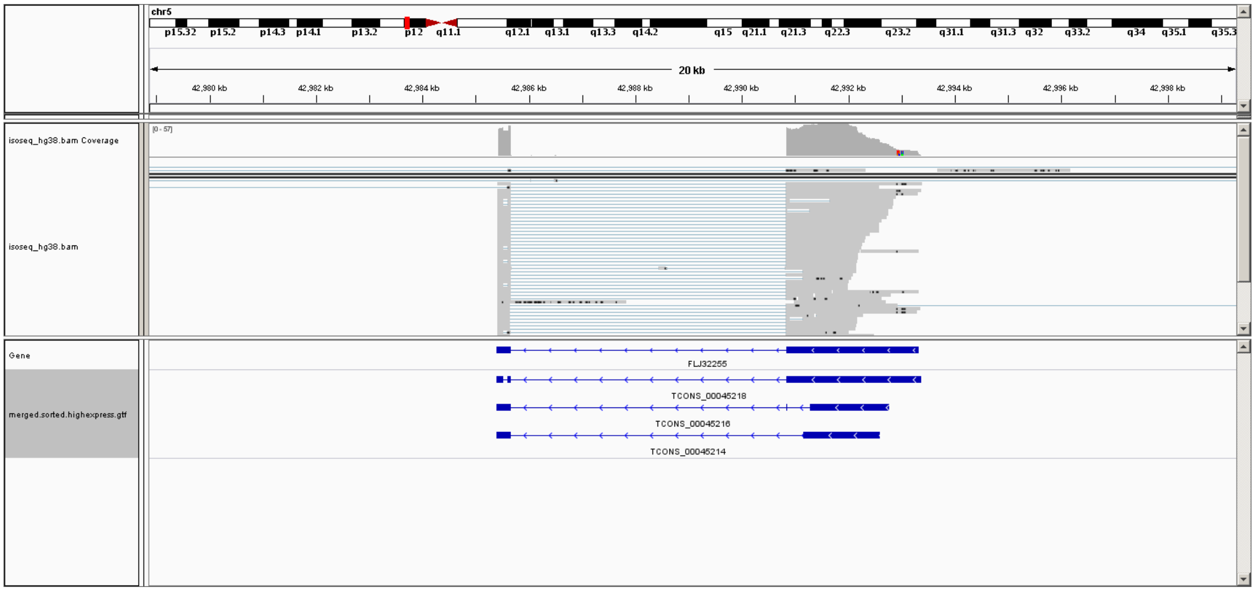
chr5
20 kb
isoseq_hg38.bam Coverage
isoseq_hg38.bam
Gene
merged.sorted.highexpress.gtf
FLJ32255
TCONS_00045218
TCONS_00045216
TCONS_00045214

C

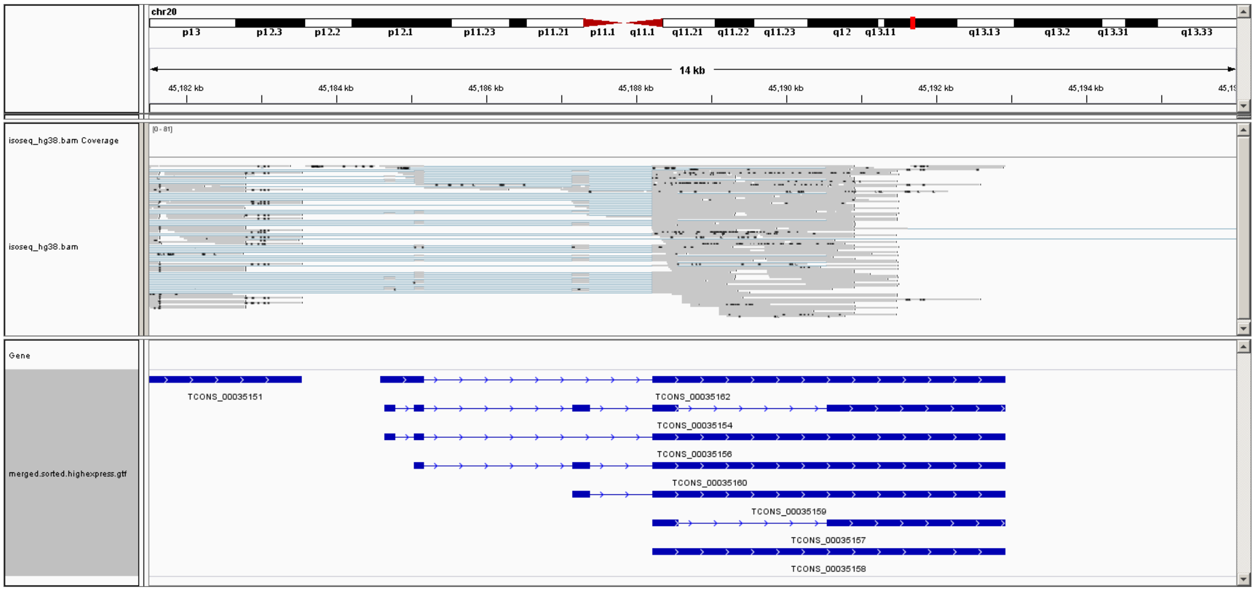
chr20
p13
p12.3
p12.2
p12.1
p11.23
p11.21
p11.1
q11.1
q11.21
q11.22
q11.23
q12
q13.11
q13.13
q13.2
q13.31
q13.33
14 kb
45,182 kb
45,184 kb
45,186 kb
45,188 kb
45,190 kb
45,192 kb
45,194 kb
isoseq_hg38.bam Coverage
[0 - 81]
isoseq_hg38.bam
Gene
merged.sorted.highexpress.gtf
TCONS_00035151
TCONS_00035162
TCONS_00035154
TCONS_00035156
TCONS_00035160
TCONS_00035159
TCONS_00035157
TCONS_00035158

D

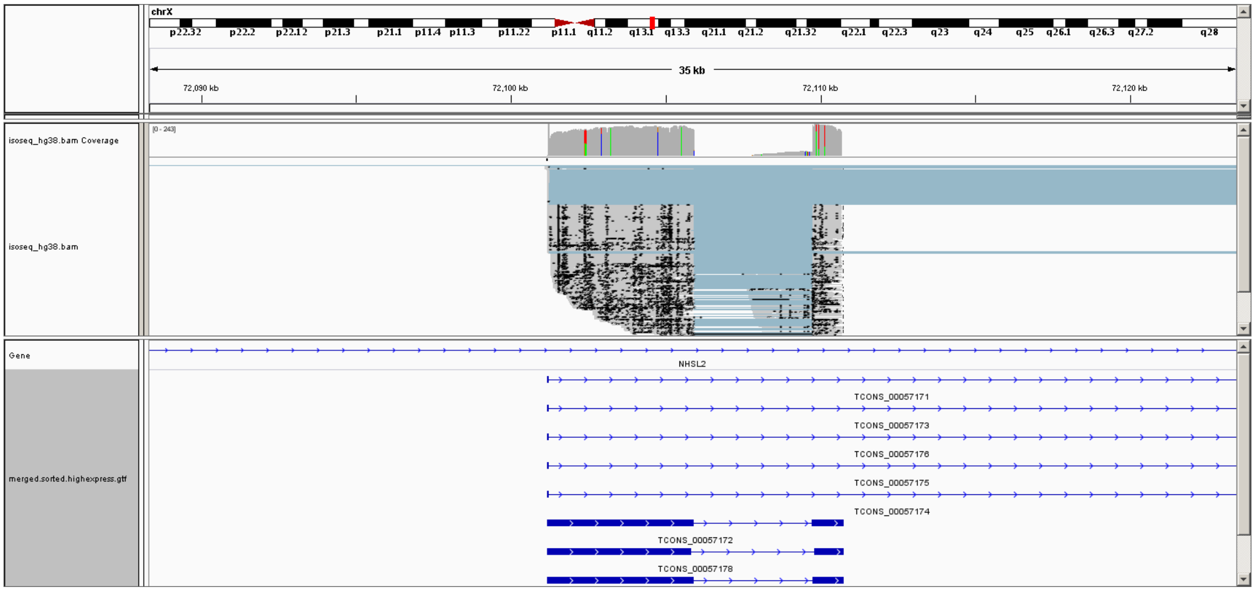
chrX
p22.32
p22.2
p22.12
p21.3
p21.1
p11.4
p11.3
p11.22
p11.1
q11.2
q13.1
q13.3
q21.1
q21.2
q21.32
q22.1
q22.3
q23
q24
q25
q26.1
q26.3
q27.2
q28
35 kb
72,090 kb
72,100 kb
72,110 kb
72,120 kb
isoseq_hg38.bam Coverage
[0 - 243]
isoseq_hg38.bam
Gene
NHSL2
merged.sorted.highexpress.gtf
TCONS_00057171
TCONS_00057173
TCONS_00057176
TCONS_00057175
TCONS_00057174
TCONS_00057172
TCONS_00057178

E

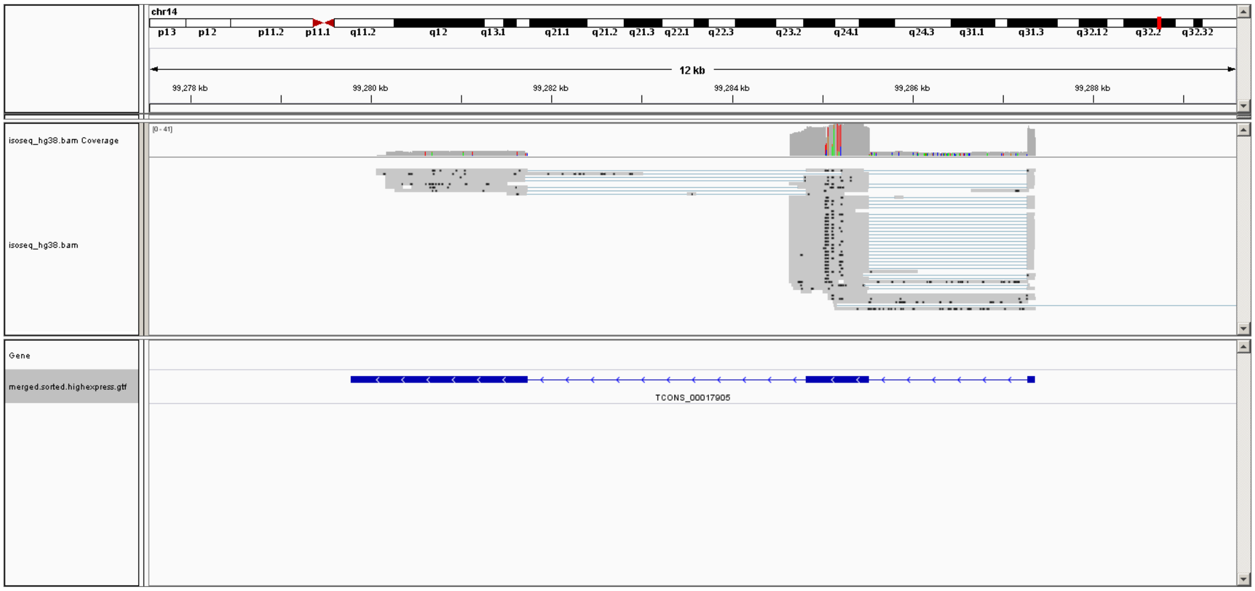

Supplementary Figure 15. Integrative Genomics Viewer screenshots of Iso-Seq alignment to GRCh38 for several novel multi-exonic transcripts. The identifier of the transcripts and its chromosomal location is listed above each figure. The upper panel lists the alignment to GRCh38, while the lower panel lists the isoforms constructed from the alignment. (A) TCONS_00051062, XLOC_026089 (chr7:3094128-3118032). (B) TCONS_00045218, XLOC_022959 (chr5:42985401-42993378). (C) TCONS_00035162, XLOC_017672 (chr20:45184587-45192935) (D) TCONS_00057178, XLOC_029360 (chrX:72109806-72110752) (E) TCONS_00017905, XLOC_009034 (chr14:99279777-99287362).

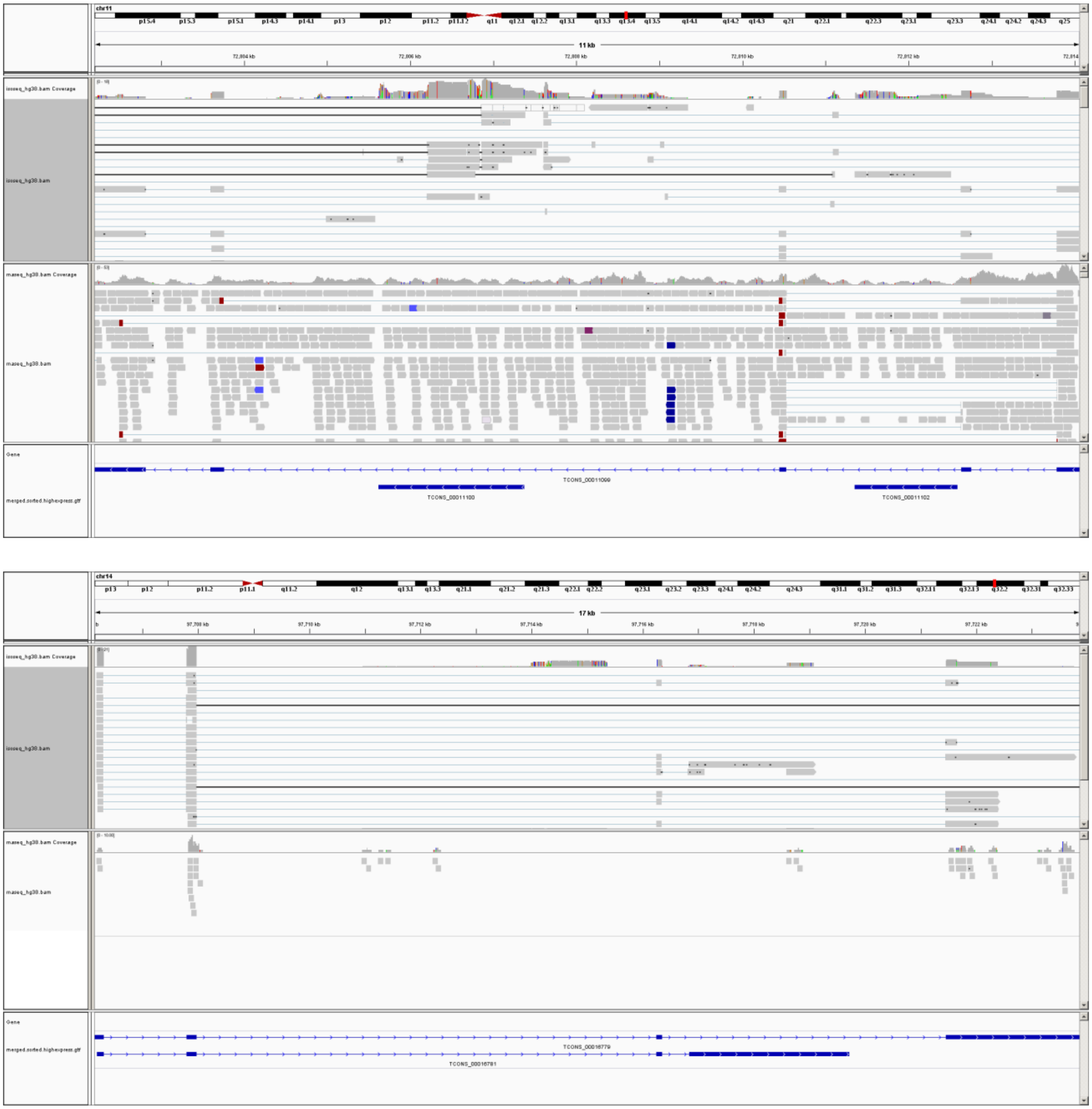

Supplementary Figure 16. The Integrative Genomics Viewer display of Iso-Seq and RNA-Seq alignments to GRCh38 at two loci (11q13.4 with one isoform, 14q32.2 with two isoforms) that are predicted to be novel genes. For both genes, the upper, middle and lower panel illustrate the Iso-Seq alignment, RNA-Seq alignment, and predicted isoforms from Iso-Seq data, respectively. RNA-Seq was not able to identify the splice patterns of the transcripts to construct isoforms; however, long-read sequencing was successful in detecting all splice events and predicting the presence of novel genes.

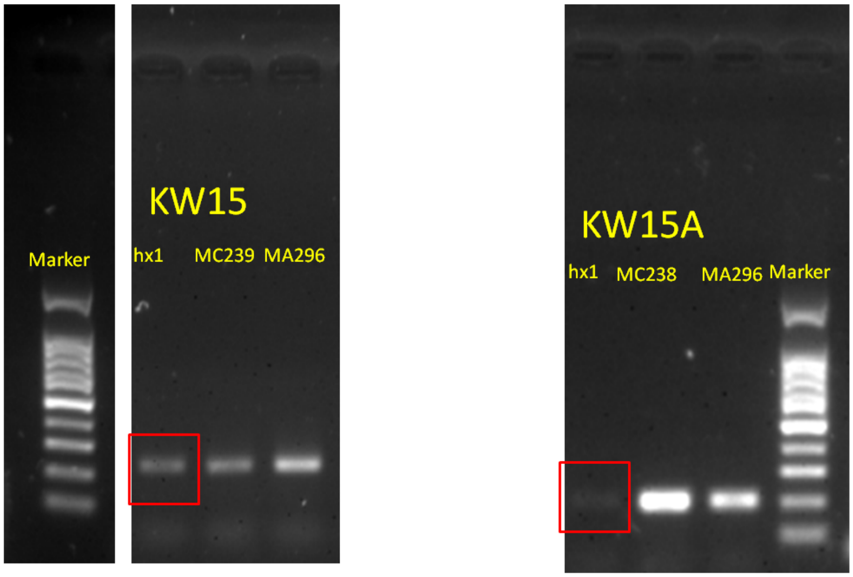

BLAT for KW15

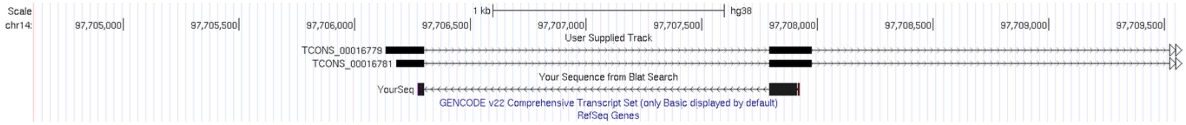

BLAT for KW15A

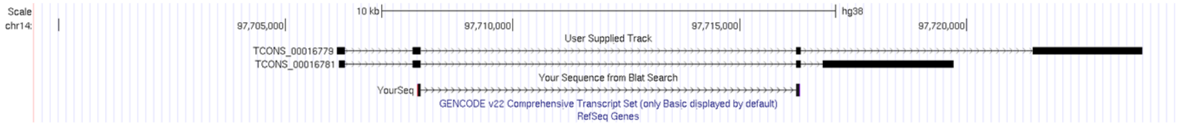

Supplementary Figure 17. Validation of splicing events in two novel genes on 14q32.2. All bands have expected sizes and are present in all four cDNA samples tested. MC239 is a Caucasian sample, while MA296 is an East Asian sample. The red circles mark DNA that were extracted from the gel for Sanger sequencing. The 100-bp ladder from G-Biosciences is used as marker for the gel. The expected product size for KW15, KW15A is 209bp and 200bp, respectively.

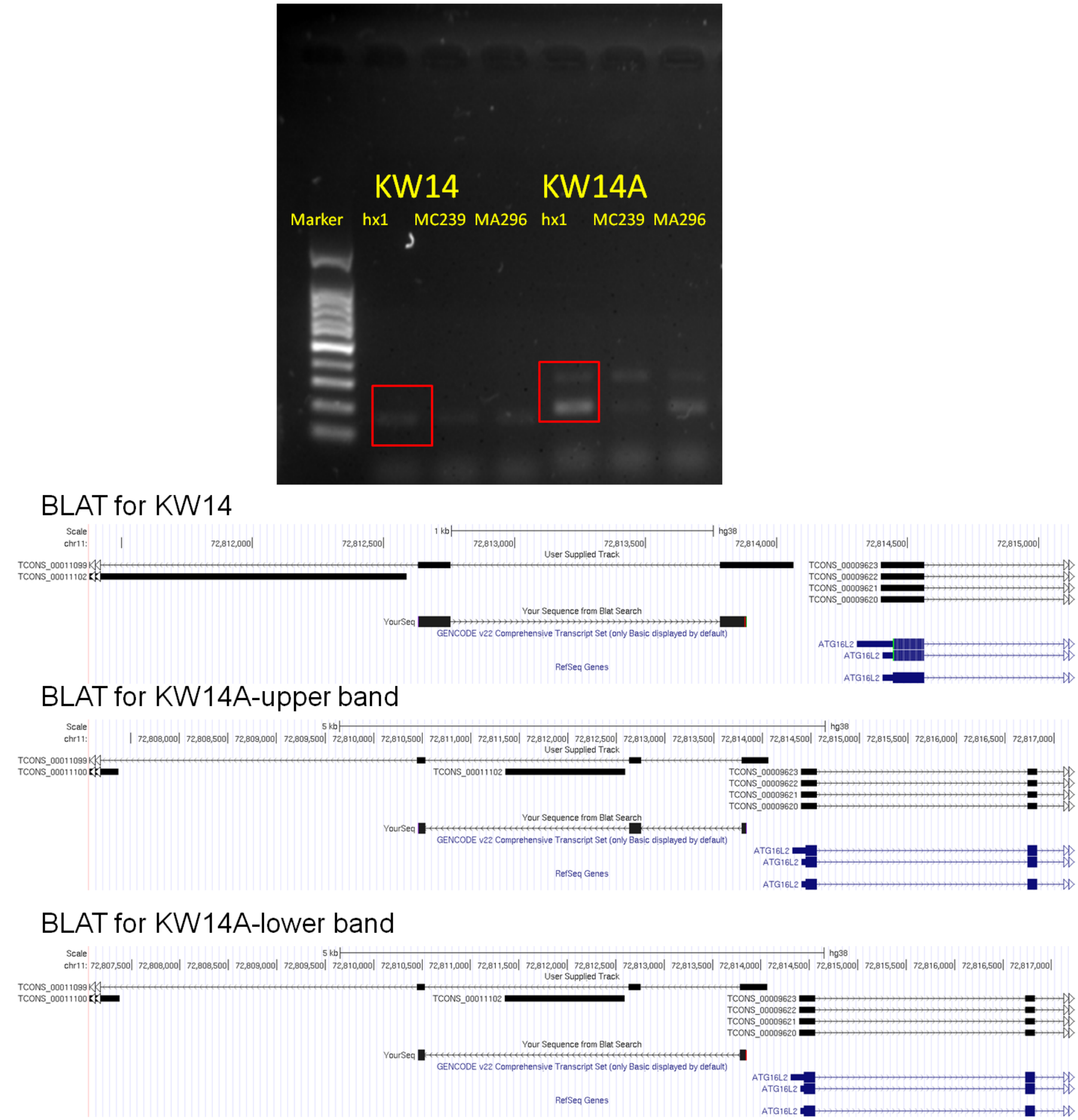

Supplementary Figure 18. Validation of splicing events in a novel gene on 11q13.4. All bands have expected sizes and are present in all cDNA samples tested. MC239 is a Caucasian sample, MA296 is an East Asian sample. The red circles mark DNA that were extracted from the gel for Sanger sequencing. The 100-bp ladder from G-Biosciences is used as marker for the gel. The expected product size for KW14, KW14A (two bands) is 150bp, 181bp and 303bp, respectively.

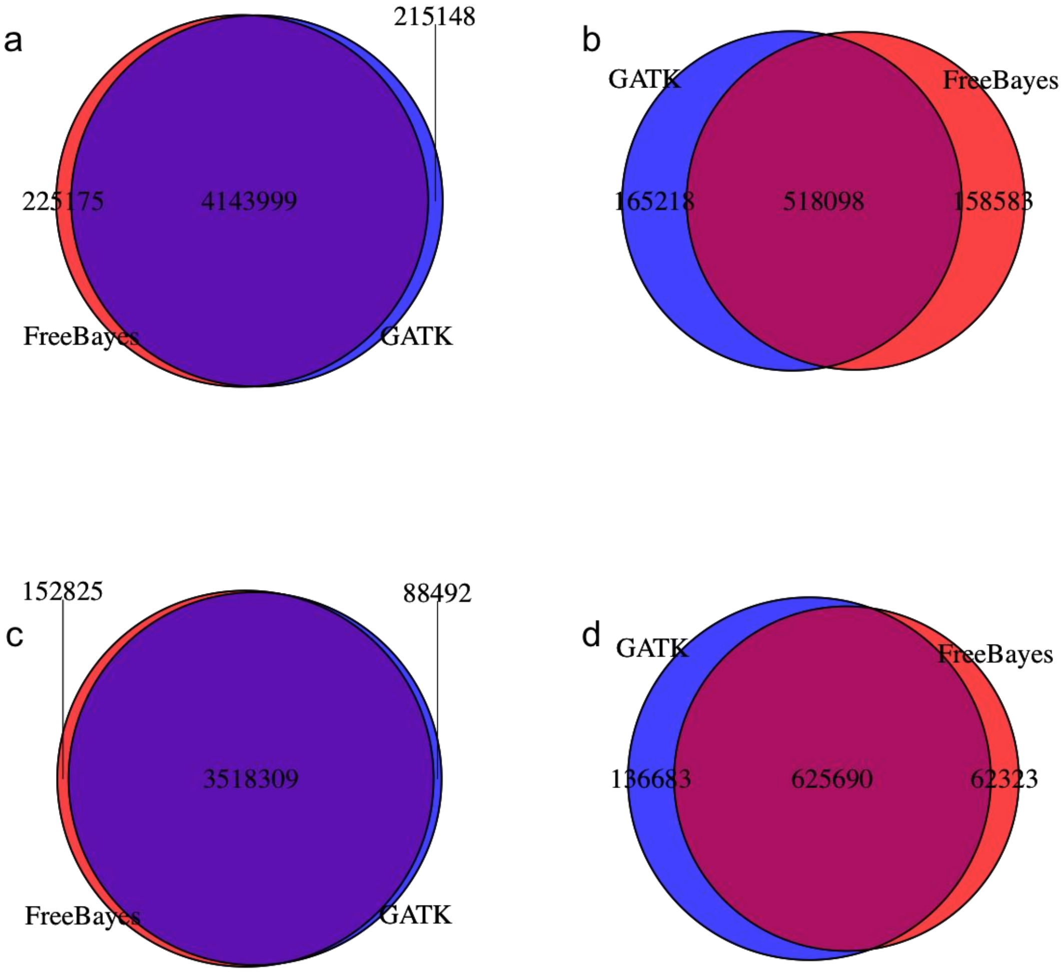

Supplementary Figure 19. Overview of consensus variants generated from GATK and Freebayes variant calling tools. These four Venn diagrams described the overlap of variants from GATK and FreeBayes variant calling tools. (A) Overlap of all variants (SNVs and Indels) generated from these two tools. (B) Overlap of rare variants (MAF<=0.01). (C) Overlap of SNVs. d. Overlap between Indels.

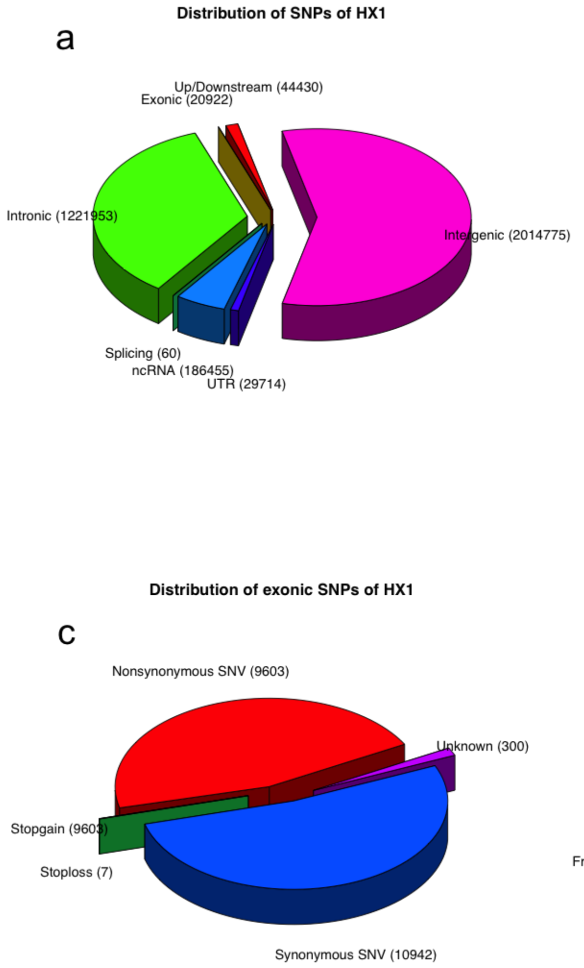

Supplementary Figure 20. Distribution of different kinds of variants among consensus SNVs and Indels generated from GATK and Freebayes. (A) Distribution of all consensus SNVs. (B) Distribution of all consensus Indels. (C) Distribution of all exonic SNVs. (D) Distribution of all exonic Indels.

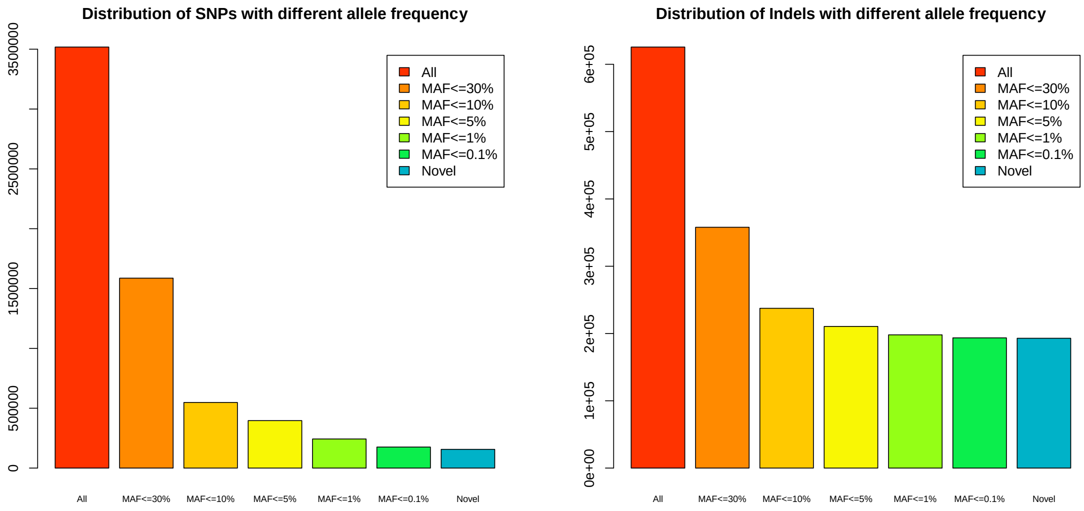

Supplementary Figure 21. Frequency of consensus variants at different MAF threshold on the Illumina whole-genome sequencing data. Left histogram describes frequency of all SNVs, SNVs with MAF<=0.3, MAF<=0.1, MAF<=0.05, MAF<=0.01, MAF<=0.001 and novel SNVs, respectively. Right histogram describes frequency of all Indels, Indels with MAF<=0.3, MAF<=0.1, MAF<=0.05, MAF<=0.01, MAF<=0.001 and novel Indels, respectively.

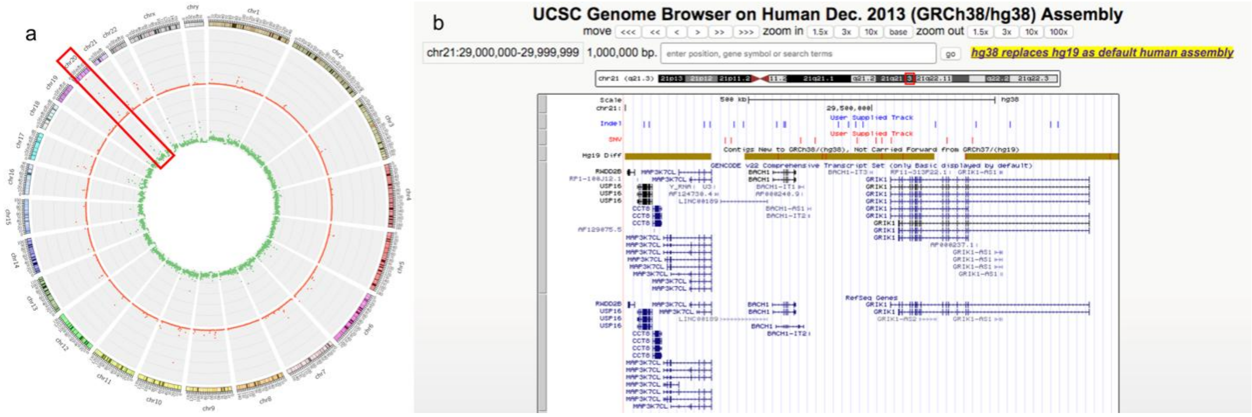

Supplementary Figure 22. Circos plot on genetic variants not in dbSNP142. (A)Circos plot on genetic variants not in dbSNP142.The tracks from outer to inner circle representthenumber of SNVs per 1Mb and the number of Indels per 1Mb, respectively. Red rectangle highlights regions with largest amount of SNVs. (B) Screenshot of UCSC Genome Browser on GRCh38. Brown bars indicate regions newly added by GRCh38. Blue bars indicate positions of the Indels in HX1 and red bars indicate positions of SNVs in HX1.

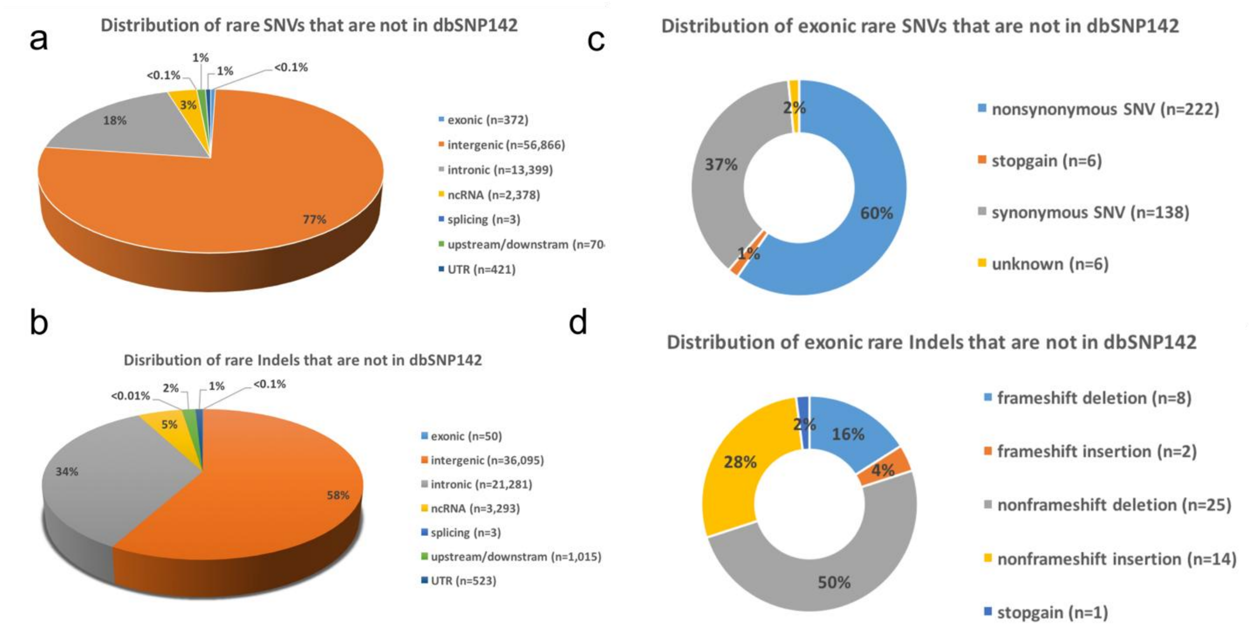

Supplementary Figure 23. Distribution of different kinds of variants among consensus rare SNVs and Indels that are not in dbSNP142. (A) Distribution of all consensus rare SNVs that are absent from dbSNP142. (B) Distribution of all consensus rare Indels that are absent from dbSNP142. (C) Distribution of all exonic rare SNVs that are absent from dbSNP142. (D) Distribution of all exonic rare Indels that are absent from dbSNP142.

Supplementary Tables

Supplementary Table 1. Statistics of the genomics data sets generated in this study.

Material	Platform	# Cells	# Reads	Bases	Coverage	Mean length	N50 length
DNA	IlluminaHiSeq X	-	2.8 billion reads	428.8 G	143X	151	151
DNA	PacBio SMRT cell	377 cells	44.2M reads	309.0G	103X	7.0Kb	12.1Kb
DNA	BioNanoIrysChip	12 cells	1.169M molecules (>150kb)	302.8G	101X	259.0Kb	224.7Kb
RNA	PacBio SMRT cell	50 cells (1-2kb, 2-3kb, 3-5kb,5kb+)	2.721M error-corrected reads	5.8G	-	2.1Kb	2.7Kb
RNA	IlluminaHiSeq 2500	NA	48.9M reads	4.4G	-	90	90

Supplementary Table 2. Statistics on subreads generated from PacBio long-read DNA sequencing on the genome DNA of HX1. Filtered subreads are used in error-correction by DALIGNER in Falcon. Error-corrected subreads are used for de novo assembly.

	**Filtered subreads**	**Error-corrected subreads**
Min	35	501
25% Quantile	1,754	2,064
Median	4,853	4,975
Mean	6,990	6,114
75% Quantile	10,531	8,621
Max	56,677	53,633
N50	12,134	9,137
Count	44,207,919	26,562,171

Supplementary Table 3. Assembly quality and sequencing depth. Assembly quality (contig N50 and assembly size) is compared with regards to sequencing depth and length cutoff for error-corrected subreads. Sequencing depth (>6kb) denotes average sequencing depth counting only reads longer than 6kb. Depth is approximately calculated as total base pairs divided by 3 Gb.

Contig N50 (Mb)	Assembly size (Gb)	Sequencing depth	Sequencing depth (>6kb)	Length cutoff for error-corrected subreads (kb)
0.61	2.68	65	48	2
2.76	2.81	81	64	2
4.65	2.87	99	80	2
6.49	2.88	99	80	9
7.16	2.87	103	83	13
8.01	2.89	103	83	11
8.28	2.88	103	83	12

Supplementary Table 4. Statistics on the filtered (>150kb) BioNano data.

Measure	Value
Total DNA	302.8Gb
Total molecules	1,169,210
Occupancy	2.58%
Average molecule SNR	16.61
Average molecule intensity	0.14
Center of mass (N50)	264.3kb
Average length	259.0kb
Median length	224.7kb
Average label density /100kb	8.92

Supplementary Table 5. RefSeq transcripts alignment results from the NCBI Assembly Evaluation pipeline

	GRCh38[1]	YH2.0[*]	NA12878[*]	HX1[#]
Number of transcripts retrieved from Entrez	50909	50909	50909	50909
Number of transcripts not aligning	22	306	455	391
Number of transcripts with multiple best alignments (split transcripts)	11	1213	1375	358
Number of transcripts with CDS coverage < 95% in aligned transcripts	16	3798	1836	808
Number of conflicting placements of transcripts from different genes	1	635	490	629
Percentage of aligned coding transcripts with CDS coverage >= 95%	99.96	90.32	95.30	97.94

[*]NCBI accession number: GCA_000004845.2 for YH2, GCA_001013985.1 for NA12878
[#] HX1 scaffolds
[1]Unless there is an assembled chrY in an assembly, our code excludes any transcript that aligns best to GRCh38 chrY from the counts for these assemblies without chrY. Therefore we excluded all transcripts best aligned to GRCh38 chrY from our analysis.

Supplementary Table 6. Repeat content within novel (non-GRCh38) sequences in HX1.

	Novel sequence in HX1			Random sequences from GRCh38		
	GC: 39.88%, 533 sequences, size: 12.8Mb			GC: 39.88%, 533 sequences, size: 12.8Mb		
	element count	length	percentage of sequence	element count	length	percentage of sequence
SINEs:	3217	298720	2.33%	6837	1586283	12.35%
ALUs	3166	291616	2.27%	4842	1290439	10.04%
MIRs	51	7104	0.06%	1977	293495	2.28%
LINEs:	280	190302	1.48%	3744	2562240	19.94%
LINE1	237	176760	1.38%	2441	2194651	17.08%
LINE2	38	12680	0.10%	1131	323749	2.52%
L3/CR1	4	270	0.00%	133	30926	0.24%
LTR	105	69993	0.54%	2081	1138178	8.86%
ERVL	21	9682	0.08%	456	237823	1.85%
ERVL-MaLRs	48	17427	0.14%	1000	421583	3.28%
ERV_classI	32	41496	0.32%	502	391458	3.05%
ERV_classII	0	0	0.00%	36	63262	0.49%
DNA elements	58	16784	0.13%	1631	389531	3.03%
hAT-Charlie	31	7348	0.06%	875	177394	1.38%
TcMar-Tigger	19	8437	0.07%	383	136871	1.07%
Unclassified:	11	14513	0.11%	37	25275	0.20%
Total interspersed repeats	3671	590312	4.59%	14330	5701507	44.38%
Satellites	5900	9666881	75.24%	21	266098	2.07%
Simple repeats	11082	1669222	12.99%	1675	100875	0.79%
Low complexity	189	9466	0.07%	1603	74223	0.58%

Supplementary Table 7. List of data sets downloaded from http://encodeproject.org for functional analysis.

Cell line	Producer	Assay	Replicate#	Accession	Control Accession
**GM12878**	broad	H3K4me3	1	ENCFF000ASR	ENCFF000ARK
**GM12878**	broad	H3K4me3	2	ENCFF000AUB	ENCFF000ARO
**GM12878**	uw	H3K4me3	1	ENCFF001EYE	ENCFF001HID
**GM12878**	uw	H3K4me3	2	ENCFF001EYF	ENCFF001HID
**GM12878**	broad	H3K4me1	1	ENCFF000ASM	ENCFF000ARK
**GM12878**	broad	H3K4me1	2	ENCFF000ATK	ENCFF000ARO
**GM12878**	broad	H3K27ac	1	ENCFF000ASP	ENCFF000ARK
**GM12878**	broad	H3K27ac	2	ENCFF000ASU	ENCFF000ARO
**GM12878**	broad	CTCF	1	ENCFF000ARP	ENCFF000ARK
**GM12878**	broad	CTCF	2	ENCFF000ARV	ENCFF000ARO
**GM12878**	stanford	CTCF	1	ENCFF000VUU	ENCFF000VWV
**GM12878**	stanford	CTCF	2	ENCFF000VUW	ENCFF000VWV
**GM12878**	uta	CTCF	1	ENCFF000ROU	ENCFF000RPB
**GM12878**	uta	CTCF	2	ENCFF000ROX	ENCFF000RPB
**GM12878**	uta	CTCF	3	ENCFF000ROZ	ENCFF000RPB
**GM12878**	uw	CTCF	1	ENCFF001HHX	ENCFF001HID
**GM12878**	uw	CTCF	2	ENCFF001HIA	ENCFF001HID
**GM12878**	uw	DNase	1	ENCFF001CUR	NA
**GM12878**	uw	DNase	2	ENCFF001CWQ	NA
**GM12878**	duke	DNase	1	ENCFF000SLF	NA
**GM12878**	duke	DNase	2	ENCFF000SLG	NA
**GM12878**	duke	DNase	3	ENCFF000SLL	NA
**GM12878**	duke	DNase	4	ENCFF000SLP	NA
**GM12878**	duke	DNase	5	ENCFF000SLR	NA

Supplementary Table 8. Mapping rate for ENCODE reads from each one of the assays from GM12878 that cannot be mapped to GRCh38 but can be realigned to HX1.

	Total number of GRCh38-unmapped reads	Total number of reads mapped to HX1	Mapping rate of GRCh38-unmapped reads
**CTCF**	52,428,376	604,755	0.011
**DNase**	133,235,297	1,078,704	0.008
**H3K4me1**	18,969,475	179,883	0.009
**H3K4me3**	8,787,823	931,154	0.106
**H3K27ac**	55,380,745	257,890	0.005
**Total**	268,801,716	3,052,386	0.011

Supplementary Table 9. Statistics of the Iso-Seq data from four libraries.

Measurement	1-2kb	2-3kb	3-5kb	5kb+
Number of cells	10	12	16	12
Read Bases of Insert	1,011,005,543	1,476,534,327	2,476,826,256	2,463,263,980
Mean Read Length of Insert	1571	2222	2615	3502
Mean Read Quality of Insert	0.9392	0.9249	0.8984	0.8727
Mean Number of Passes	11	8	7	5
Number of full-length non-chimeric reads	419980	374309	327283	258017
Average full-length non-chimeric read length	1338	2087	2750	3550

Supplementary Table 10. Quality statistics for each of the four Iso-Seq libraries after error-correction. All reads were used in the error correction procedure.

Library	Original reads #	Corrected reads #	Percentage of retrieved reads	Average length (before correction)	Average length (after correction)	Coverage of short reads on long reads*
**1-2kb**	612260	590435	96.40%	1560.32	1264.86	0.93(0.17)
**2-3kb**	645396	624965	96.80%	2203.25	1863.42	0.89(0.2)
**3-5kb**	915201	870807	95.10%	2622.74	2248.31	0.88(0.21)
**5kb+**	690271	659582	95.50%	3518.17	2990.85	0.81(0.26)

*mean(standard deviation)

Supplementary Table 11. Full list of novel transcripts identified from the Iso-Seq data.

Transcript	Gene	Region	Length	read count on gene	#exon
**TCONS_00000361**	XLOC_000194	chr1:21583070-21584883	1813	26	1
**TCONS_00003639**	XLOC_001770	chr1:28124918-28126933	2015	255	1
**TCONS_00001347**	XLOC_000695	chr1:101287668-101293284	5616	508	1
**TCONS_00005233**	XLOC_002533	chr1:159910915-159913857	2942	35	1
**TCONS_00006052**	XLOC_002951	chr1:244225028-244254637	7651	47	3
**TCONS_00033286**	XLOC_016666	chr2:69953727-69956926	3199	170	1
**TCONS_00031944**	XLOC_015954	chr2:172611111-172614723	3612	40	1
**TCONS_00032050**	XLOC_016018	chr2:191700365-191707182	6817	21	1
**TCONS_00038559**	XLOC_019270	chr3:16126180-16131659	5479	20	1
**TCONS_00040458**	XLOC_020283	chr3:44491768-44494532	2764	29	1
**TCONS_00040973**	XLOC_020526	chr3:72000153-72005541	5388	25	1
**TCONS_00041113**	XLOC_020595	chr3:114332087-114335460	3373	26	1
**TCONS_00045214**	XLOC_022959	chr5:42985401-42992597	1706	63	2
**TCONS_00045216**	XLOC_022959	chr5:42985401-42992768	1778	63	3
**TCONS_00045218**	XLOC_022959	chr5:42985401-42993378	2719	63	3
**TCONS_00044293**	XLOC_022455	chr5:100903806-100905745	1939	38	1
**TCONS_00045564**	XLOC_023168	chr5:91285159-91288348	3189	42	1
**TCONS_00044685**	XLOC_022653	chr5:148853985-148865352	1787	65	2
**TCONS_00044687**	XLOC_022653	chr5:148869686-148873470	3784	65	1
**TCONS_00046028**	XLOC_023403	chr5:151728940-151731369	2429	23	1
**TCONS_00048548**	XLOC_024711	chr6:42200025-42201855	1830	27	1
**TCONS_00051062**	XLOC_026089	chr7:3094128-3118032	1673	20	3
**TCONS_00051063**	XLOC_026089	chr7:3094128-3118050	1533	20	2
**TCONS_00049669**	XLOC_025346	chr7:30208555-30213859	5304	52	1
**TCONS_00052448**	XLOC_026811	chr8:2196838-2202346	5398	20	2
**TCONS_00052857**	XLOC_027014	chr8:43128072-43131176	3104	25	1
**TCONS_00052895**	XLOC_027035	chr8:55867316-55879714	5521	27	2
**TCONS_00056385**	XLOC_028969	chr9:128663672-128664715	1043	103	1
**TCONS_00055273**	XLOC_028401	chr9:129153047-129154140	1093	106	1

**TCONS_00008021**	XLOC_004091	chr10:88072687-88078283	5596	25	1
**TCONS_00008023**	XLOC_004093	chr10:88099948-88104408	4460	28	1
**TCONS_00006926**	XLOC_003454	chr10:95697671-95700348	2677	176	1
**TCONS_00011099**	XLOC_005542	chr11:72802199-72814065	1271	23	5
**TCONS_00015652**	XLOC_007901	chr13:49956692-49957702	1010	61	1
**TCONS_00015653**	XLOC_007901	chr13:49956692-49981668	5539	61	2
**TCONS_00015655**	XLOC_007901	chr13:49975131-49980139	5008	61	1
**TCONS_00017319**	XLOC_008694	chr14:50100480-50105217	4737	70	1
**TCONS_00016243**	XLOC_008166	chr14:52280382-52285958	5576	34	1
**TCONS_00017876**	XLOC_009018	chr14:95503639-95510874	7235	30	1
**TCONS_00016779**	XLOC_008462	chr14:97706134-97723869	2863	26	4
**TCONS_00016781**	XLOC_008462	chr14:97706178-97719715	3284	26	4
**TCONS_00017905**	XLOC_009034	chr14:99279777-99287362	2751	46	3
**TCONS_00019269**	XLOC_009707	chr15:44723731-44726775	3044	30	1
**TCONS_00019267**	XLOC_009707	chr15:44723731-44726775	2087	30	2
**TCONS_00018591**	XLOC_009393	chr15:75140334-75141310	976	30	1
**TCONS_00021687**	XLOC_010935	chr16:11538423-11545594	5440	1989	2
**TCONS_00022111**	XLOC_011158	chr16:50651442-50658848	7406	373	1
**TCONS_00022537**	XLOC_011363	chr16:88632892-88634426	1534	37	1
**TCONS_00027736**	XLOC_013892	chr19:21596221-21599046	2013	47	2
**TCONS_00035151**	XLOC_017672	chr20:45179799-45183542	3551	195	2
**TCONS_00035156**	XLOC_017672	chr20:45179817-45192935	5165	195	5
**TCONS_00035154**	XLOC_017672	chr20:45179817-45192935	3565	195	6
**TCONS_00035157**	XLOC_017672	chr20:45179888-45192935	2983	195	3

**TCONS_00035158**	XLOC_017672	chr20:45179889-45192935	4944	195	2
**TCONS_00035159**	XLOC_017672	chr20:45180041-45192935	5040	195	3
**TCONS_00035160**	XLOC_017672	chr20:45180058-45192935	5139	195	4
**TCONS_00035162**	XLOC_017672	chr20:45184587-45192935	5306	195	2

Supplementary Table 12. List of primers used in qPCR experiments on cDNA to validate the expression of transcripts.

Primer ID	Sequence	Locus	Orientation
**4-1-F-cDNA**	AGCGGAGGTCTGAAGAACAA	XLOC_026089	F
**4-2-F-cDNA**	CAGTTGGGGTAGGGAGATGA	XLOC_022959	F
**4-3-F-cDNA**	TCACCACCAAAAGAGGGAAA	XLOC_017672	F
**4-4-F-cDNA**	CCACCAGGTGAAAGAAGGTT	XLOC_029360	F
**4-7-F-cDNA**	GTCTGGGAGCCACCTTCTCT	XLOC_009034	F
**4-1-R-cDNA**	GCCCCATACGTTTCAAGAGA	XLOC_026089	R
**4-2-R-cDNA**	TCACCACTTCTGCTCCTGTG	XLOC_022959	R
**4-3-R-cDNA**	TCAGGGGCTGAGACAGAGTT	XLOC_017672	R
**4-4-R-cDNA**	CCCAGCCTTTCTCAATGAAG	XLOC_029360	R
**4-7-R-cDNA**	CCCAGGCCTGAGCTTTCT	XLOC_009034	R
**N1-F**	TTCTGCAAAGTGCTGGGTTC	neg_control	F
**N1-R**	CCTGGGTCTGAGTCAGCTCT	neg_control	R
**ACTB-F**	ACATCCGCAAAGACCTGTACG	pos_control	F
**ACTB-R**	ACGGAGTACTTGCGCTCAGG	pos_control	R
**2-3-1**	TGCACTTGAAGAAGATCAAGAAA	XLOC_017672	F
**2-3-1**	GGCATTCTTTCCCAGAACAA	XLOC_017672	R
**2-3-1-NEST**	AGAAAGCGTGAGTAATGTTTTGG	XLOC_017672	F
**2-3-1-NEST**	TGATGAGTGTCCAGGACTGC	XLOC_017672	R
KW14	CACACTGGAGTGCAACTGCT	XLOC_005542	F
KW14	GAACTTCAGGAAAGCCAGGA	XLOC_005542	R
KW14A	GCCAAGCCTGGTTCTAGATG	XLOC_005542	F
KW14A	AGTTGGGTCTGTGAGGGATG	XLOC_005542	R
KW15	GAGCAACCATTAACCCTGGA	XLOC_008462	F
KW15	TCCACAGCACTTGATCTTGC	XLOC_008462	R
KW15A	GGCTGGGTCCCATTCTTTAT	XLOC_008462	F
KW15A	CAACATCAAAGGCATAATCCA	XLOC_008462	R

Supplementary Methods

Genome/transcriptome sequencing

Study participants

For genome sequencing, an anonymous male individual (identifier: HX1) without documented history of chronic disease was collected for the study at Jinan University, Guangdong, China. The individual provided written consent for public release of genomic data. The individual has a family pedigree traceable to 5 generations in central China. He did not report a family history of known genetic disorders. The Institutional Review Board (IRB) at the Jinan University reviewed and approved the initial study, and informed consent was obtained from all participants.

For validation of novel transcripts, existing DNA/RNA samples from HX1 and multiple additional anonymous subjects from three ethnicity groups (East Asian, African, European) were obtained from the Nationwide Children's Hospital. Furthermore, EBV-transformed lymphoblastoid cell lines on HX1 using whole blood were also made at the Nationwide Children's Hospital. The Institutional Review Board (IRB) at the Children's National Hospital reviewed and approved the validation study, and informed consent was obtained from all participants.

PacBio DNA sequencing

Lymphocytes from freshly drawn whole blood were isolated using Ficoll-Paque PLUS (17-1440-02). To ensure the highest quality for library construction, high-molecular-weight DNA (20-50 kb) DNA was extracted from isolated lymphocytes using Phenol-Chloroform method, as detailed in https://pacbio.secure.force.com/servlet/servlet.FileDownload?file=00P7000000CtfqbEAB.

Briefly, high-quality genomic DNA was verified using high sensitivity Qubit analysis to quantify the mass of double-stranded DNA and pulsed-field gel electrophoresis to qualify the integrity of gDNA. After quantification, DNA was diluted to 150 µL using Qiagen elution buffer at 40 µg / µL. The 150 µL aliquot was individually pipetted into the top chamber of a Covaris G-tube spin column and sheared gently for 60 seconds at 4000 rpm using an Eppendorf 5424 bench top centrifuge. Once completed, the spin column was flipped after verifying that all DNA was now in the lower chamber. Then, the column was spun for another 60 seconds at 4000 rpm to further shear the DNA and place the aliquot back into the upper chamber, resulting in a 10,000 to 25,000 bp DNA shear, verified using a DNA 12000 Agilent Bioanalyzer gel chip. The sheared DNA was then re-purified using a 0.45X AMPure XP purification step (0.45X AMPure beads added, by volume, to each DNA sample dissolved in 200 µL elution buffer (EB), vortexed for 10 minutes at 2,000 rpm, followed by two washes with 70% alcohol and finally diluted in EB). After shearing and purification , ~4.5-5ug of purified and sheared sample was taken into DNA damage and end-repair from each batch preparation. Briefly, the DNA fragments were repaired using DNA damage repair solution (1X DNA damage repair buffer, 1X NAD+, 1 mM ATP high, 0.1 mM

dNTP, and 1X DNA damage repair mix, Pacific Biosciences's SMRTbell template prep kit 1.0 (100-259-100) with a volume of 21.1 µL and incubated at 37°C for 20 minutes. DNA ends were repaired next by adding 1X end repair mix to the solution, which was incubated at 25°C for 5 minutes, followed by the second 0.45X Ampure XP purification step. Next, 0.75 µM of blunt adapter was added to the DNA, followed by 1X template preparation buffer, 0.05 mM ATP low and 0.75 U/µL T4 ligase to ligate (final volume of 47.5 µL) the SMRTbell adapters to the DNA fragments. This solution was incubated at 25°C overnight, followed by a 65°C 10-minute ligase denaturation step. After ligation, the library was treated with an exonuclease cocktail to remove un-ligated DNA fragments using a solution of 1.81 U/µL Exo III 18 and 0.18 U/µL Exo VII, then incubated at 37°C for 1 hour. One additional 0.45X Ampure XP purification step was performed to remove < 2,000 bp molecular weight DNA and organic contaminant. Then Blue Pippin size selection was carried out in DNA combined from two libraries. This step was conducted using Sage Science Blue Pippin 0.75% agarose cassettes to select library in the range of 7,000 bp-50,000 bp. Then the size-selected library was repaired again using DNA damage repair solution. Size-selection was confirmed by Bio-Analysis and the DNA was quantified using Qubit assay.

SMRTbell templates were bound to polymerase by DNA/Polymerase Binding Kit P6 (100-356-300) and V2 primers. Primer was annealed to the size-selected SMRTbell at a ratio of 20X with the full-length libraries (80°C for 2 minute followed by decreasing the temperature by 0.1°C /s to 25C^{o}). The polymerase-template complex was then bound to the P6 enzyme using a ratio of 10:1 polymerase to SMRTbell at 0.5 nM for 4 hours at 30°C and then held at 4°C until ready for magbead loading, prior to sequencing. The magnetic bead-loading step was conducted at 4°C for 60-minutes. The magbead-loaded, polymerase-bound SMRTbell libraries were placed onto the RSII machine with150-400 pM C4 sequencing reagents. RSII was configured for 240-minute continuous sequencing.

PacBio RNA sequencing

For Iso-Seq, total RNA was extracted directly from freshly drawn blood using TRIzol extraction reagent (Life Technologies, Grand Island, NY). We used high sensitivity Qubit analysis to quantify the mass of RNA and Agilent BioAnalyzer 2100 to qualify the integrity of RNA. After quantification and quality control analysis, RNA was diluted to 2.5 µL using Qiagen elution buffer at 454 ng/µL. First strand cDNA synthesis employs the Clontech SMARTer PCR cDNA Synthesis Kit. The CDS Primer IIA is first annealed to the polyA+ tail of transcripts, followed by first-strand synthesis with SMARTScribe™ Reverse Transcriptase. The first-strand product was diluted with Elution Buffer (EB) to 40 ul. After PCR amplifications test we chose 17 cycles for large-scale PCR. The large-scale PCR product was purified using a 1.0X AMPure XP purification step (1.0X AMPure beads added, by volume, to cDNA sample dissolved in 20 µL elution buffer (EB), vortexed for 10 minutes at 2,000 rpm, followed by two washes with 70% alcohol and finally diluted in EB). Once completed, the cDNA product was loaded onto BluePippin™ System and recovered 4 fractions with different sizes: 1-2kb, 2-3kb, 3-5kb, >5kb. After size selection,

we performed large-scale PCR on each fraction to generate enough double-stranded DNA for library preparation.

After purification ~1 to 2 µg of purified and size selected fraction was subject to DNA damage and end repair. Briefly, the different fractions were repaired using DNA damage repair solution (1X DNA damage repair buffer, 1X NAD+, 1 mM ATP high, 0.1 mM dNTP, and 1X DNA damage repair mix) with a volume of 30 µL and incubated at 37°C for 20 minutes. DNA ends were repaired next by adding 1X end repair mix to the solution, which was incubated at 25°C for 5 minutes, followed by 1.0X Ampure XP purification step. Then 0.75 µM of blunt adapter was added to the DNA, followed by 1X template preparation buffer, 0.05 mM ATP low and 0.75 U/µL T4 ligase to ligate (final volume of 47.5 µL) the SMRTbell adapters to the DNA fragments. This solution was incubated at 25°C overnight, followed by a 65°C 10-minute ligase denaturation step. After ligation, the library was incubated at 37°C for 1 hour with an exonuclease cocktail to remove un-ligated DNA fragments. Exonuclease cocktail consisted of 1.81 U/µL Exo III 18 and 0.18 U/µL Exo VII. One additional size selection was performed with BluePippin™ System to remove smaller or larger DNA fragment outside the targeted region of each fraction and organic contaminant. Upon completion of library construction, samples were validated as desired sizes using another Agilent DNA 12000 gel chip and quantified with high sensitivity Qubit analysis. Then, primer was annealed to the size-selected SMRTbell at a ratio of 20X with the full-length libraries (80°C for 2 minute followed by decreasing the temperature by 0.1°C /s to 25°C). The polymerase-template complex was then bound to the P6 enzyme using a ratio of 10:1 polymerase to SMRTbell at 0.5 nM for 4 hours at 30°C and then held at 4°C until ready for magbead loading. The magnetic bead-loading step was done at 4°C for 60-minutes. The magbead-loaded, polymerase-bound, SMRTbell libraries were placed onto the RSII machine at different sequencing concentration (10pM for 1-2kb library, 15pM for 2-3kb library, 20pM for 3-5kb library, 30pM for >5kb library) for a 240-minute continuous sequencing run.

Illumina DNA sequencing

Standard "purple-top" Vacutainer tubes with EDTA (BD Biosciences, San Jose, CA, USA) were used for blood extraction and storage. The DNeasy DNA & Tissue kit from Qiagen (Valencia, CA, USA) was used to extract DNA. A total of 50µL DNA sample (34ng/µL) was sent to sequencing facility. The Illumina TruSeqDNA Nano library preparation protocol was used to generate sequencing libraries. The short-read whole-genome sequencing data was generated on HiSeq X DNA sequencer, and 151bp paired-end reads were generated.

In total, we obtained 2.8 billion reads (429 billion bases), with an estimated coverage of 143X. The raw FASTQ files were processed by the SeqMule pipeline[1] (http://seqmule.openbioinformatics.org) for whole genome data, which performs automated sequence quality control, alignment (BWA 0.7.12 [2]), alignment deduplication (SAMtools), variant calling (GATK 3.4-46 haplotype caller[3], freebayes[https://github.com/ekg/freebayes]), and variant filtering. The variant annotation and downstream analysis were performed by ANNOVAR[4] and custom scripts.

Illumina RNA sequencing

For Illumina RNA sequencing, the same total RNA as described in PacBio Iso-Seq was used. Library generation was performed according to the TruSeq mRNA Library Kit (Illumina). Quality control of the generated libraries was performed on a BioanalyzerDNA 2100 chip (Agilent), and the concentration was measured using Qubit dsDNA HS Assay (Life Technologies). The 350 bp libraries were pooled to 10 nM total concentration and sequenced on an Illumina HiSeq 2500 sequencer.

BioNano genome mapping

We used the Irys System from BioNano Genomics, a NanoChannel-based fluidic IrysChip that can unravel, sort, and confine native-state, long, genomic DNA fragments in a linearized conformation. The DNA is labeled by a nicking enzyme that recognizes characteristic 7-mer in genome sequence. Labeled sites appear as a uniquely recognizable pattern of “dots on a string”, so that once the DNA is stretched inside the NanoChannels, the Irys CCD detector optically images them. Uniquely, the straightened molecules in solution are able to move smoothly through the nanoscale fluidic environment, enabling multiple cycles of automated loading and imaging for high-throughput scanning and analysis.

The BioNano optical mapping data was generated using DNA extracted from 3mL freshly drawn whole blood, using manufacturer recommended protocols for library preparation and optical scanning. The default nicking enzyme NT.BspQI was used for digesting DNA.A total of 12 flow cells were used for this DNA analysis. We selected 150kb as the threshold for size filter (those molecules <150kb are removed from raw data), and selected dynamic SNR (signal-to-noise ratio) filter which allows IrysView to automatically calculate the optimal label SNR. The final set of cleaned data includes 302.8Gb data on 1,169,210 molecules, with N50 of 264.3kb (Supplementary Table 1). Of note, 30.0Gb (9% bin mass fraction) of the data has length over 500kb. The IrysView software (software version 2.1.1.8025 with RefAligner/Assembly version r3827), in a desktop computer was used for data reformatting, QC and visualization, yet heavy-duty data analysis (such as assembly, scaffolding and genome comparison) was performed using a computing cluster. Additional procedures for data analysis are described in several sections below.

Karyotyping for chromosomal abnormality

To assess large-scale chromomosal abnormality or aneuploidy in the HX1 genome, we performed karyotyping at the Clinical Diagnostic Laboratory at the Women and Children's Hospital of Guangdong. Standard karyotyping techniques are used on 5mL freshly drawn blood. Briefly,after a short-term culture of cells derived from the blood, dividing cells are arrested in metaphase by addition of colchicine, which poisons the mitotic spindle. The cells are next treated with a hypotonic solution that causes their nuclei to swell and the cells to burst. The

cells were then examined on a glass slide with stains to examine the structural features of the chromosomes.

Validation of gene expression and Sanger sequencing

To validate the presence of candidate transcripts, we used PCR on cDNA from HX1 and other anonymous individuals. Given the chromosomal position of predicted transcripts, we designed PCR primers to amplify fragments harboring a portion of the transcripts by Primer3[5]. The pair of PCR primers was designed to encompass introns in the genome sequence, so that PCR on cDNA can be used to validate splicing events. To confirm the identity of the PCR products, the bands were cut off gel and DNA were recovered using Qiagen QIAquick Gel Extraction Kit (Valencia, CA) and sequenced by Sanger sequencing. The ABI 3730 XL sequencer was used for sequencing, and the resulting *.AB1 files were loaded into the ABI Sequence Scanner Software v1.0 for further analysis.

Construction of reference genome

de novo draft genome assembly by Falcon

After we obtained raw data, we performed subread filtering using the SMRT Portal software (Pacific Biosciences, Menlo Park, CA) with default settings for subread protocol. FASTA files were then pooled together for assembly. The average read length and N50 are 7.0kb and 12.1kb, respectively. Due to the many random errors in SMRT long reads, a hierarchical genome assembly process assembler called Falcon[6] specifically designed for PacBio reads was used to do *de novo* assembly. In particular, we modified Falcon to improve its performance in an NFS-based computing cluster. The source code is available on github (https://github.com/WGLab/EnhancedFALCON).

Unlike hybrid assembly method that combines SMRT long reads and Illumina short reads, Falcon relies on single source of sequencing data for assembly. It first uses DALIGNER[7] to find overlaps between filtered subreads and then extracts consensus sequences from overlaps (pre-assembly). The consensus sequences are much more accurate than subreads[6]. DALIGNER is called again to find overlaps between error-corrected reads. Based on these overlaps, Falcon constructs a graph where a node denotes a read and a directed edge denotes an overlap. By removing redundant edges and resolving complicated paths, simple paths can be identified and later be converted to contigs. Because human genome is diploid, alternative paths may exist in certain regions. As a result, associate contigs will also be constructed. A visualization of assembly procedure is shown in the figure below.

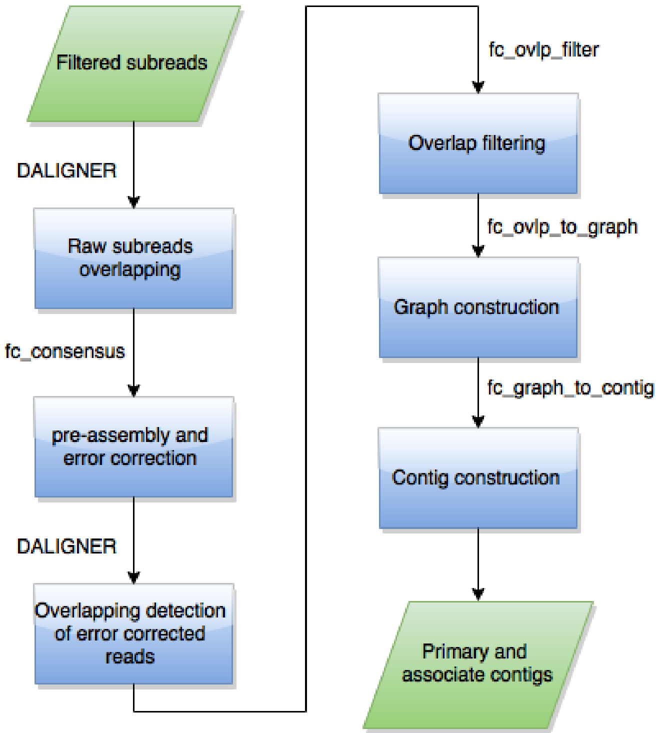

All reads were used to build the assembly ("-a" option in DBsplit). Length cutoff is 6,000 bp for error-correction and 12,000 bp for graph construction. Detailed configuration is provided as follows.

```
# The length cutoff used for seed reads used for initial mapping
length_cutoff = 6000

# The length cutoff used for seed reads usef for pre-assembly
length_cutoff_pr = 12000

#-M means limit memory usage, use integer
pa_HPCdaligner_option =  -v -dal128 -t16 -e.70 -l1000 -s1000
ovlp_HPCdaligner_option = -v -dal128 -t32 -h60 -e.96 -l500 -s1000

#-x is length threshold for including reads in DB
#consider add -a option in future to include secondary reads for error correction
pa_DBsplit_option = -x500 -s400 -a
ovlp_DBsplit_option = -x500 -s400 -a

falcon_sense_option = --output_multi --min_idt 0.70 --min_cov 4 --max_n_read 200 --n_core 6
overlap_filtering_setting = --max_diff 60 --max_cov 100 --min_cov 2 --bestn 10 --n_core 12
```

Evaluation of draft genome quality by BioNano

We next used the BioNano data as an orthogonal genome mapping approach to evaluate the quality and completeness of the draft genome assembly. For this analysis, we compared the draft genome with GRCh38, to assess whether BioNano tend to be better mapped to one genome versus the other. This analysis is not fair, since a draft assembly contains many short contigs, and some BioNano reads may not be mappable to these short contigs, resulting in

reduced mapping rate. However, the difference between draft assembly and a reference genome assembly (GRCh38) can inform us about the completeness of the draft assembly.

Using the "Molecular Quality Report" function in the BioNano IrysView software (version 2.1.1.8025) with RefAligner/Assembly (version r3827), we evaluated what fraction of BioNano molecules (>150kb) can be mapped to the draft genome assembly and the GRCh38 reference assembly, respectively. The parameters used are " -nosplit 2 -BestRef 1 -biaswt 0 -Mfast 0 -FP 1.5 -sf 0.2 -sd 0.0 -A 5 -outlier 1e-4 -endoutlier 1e-3 -S -1000 -sr 0.04 -resbias 5 64 -maxmem 36 -M 3 -minlen 150 -T 1e-9 -maxthreads 12 -hashgen 5 3 2.4 1.5 0.05 5.0 1 1 2 -hash -hashdelta 10 -hashmaxmem 36 -insertThreads 8 -stdout -stderr". These are the default parameters in the software, except that we used a highly stringent threshold (T=1e-9) to declare a match and sampled 50,000 molecules (rather than 5,000 as shown by default settings), per suggestions from BioNano to process human genomes. The mapping rates of BioNano molecules to the draft assembly and GRCh38 are 78.9% and 80.2%, respectively.

Construction of physical map by BioNano nanochannel array

The BioNano NanoChannel Array (Irys System) linearizes DNA molecules up to megabases in length, and uses nicking enzymes that recognize characteristic 7-mer in genome sequence to provide physical maps. This method therefore allows de novo assembly of the human genome, and such assembly can be further used as scaffolds for PacBio-assembled genomic sequences. This approach has already been used recently for analyzing human genomes[8].

We performed de novo genome assembly on the filtered data, with manufacturer-recommended parameters for human genome (molecular length threshold: 150kb; min label per molecule: 8; maximum backbone intensity: 0.6; false positive density/100kb: 1.5; false negative rate: 0.15%; Scaling SD: 0; siteSD: 0.2kb; relative SD: 0.03; initial assembly p-value cutoff: 1e-10; extension and refinement p-value cutoff: 1e-11; merge p-value cutoff: 1e-15), autonoise adjustment, and 5 iterations of computation. The final results contain 2,346 contigs with the N50 for the assembly as 1.80Mb.

Improve draft genome assembly by hybrid scaffolding

After de novo assembly of the physical map using BioNano data, we used the physical map as scaffolds for PacBio-assembled draft genome. This approach is referred to as "hybrid scaffolding", which identifies regions that PacBio contigs can be grouped together by BioNano scaffold, and generate new assembled sequences with improved N50 values, as demonstrated in a recent study (N50=906kb for PacBio data, and N50=13.6Mb after hybrid scaffolding by BioNano data) [8].

With extensive discussion with BioNano, we developed the following strategies and wrote a few custom scripts (available upon requests) for post-processing of hybrid scaffolds and generating the complete genome sequence. This procedure is described here:

(1) Use IrysView software to perform hybrid scaffolding, with BioNano assembly as the scaffold, and PacBio assembly (as raw FASTA sequence rather than enzyme-digested cMAP) as the query sequence.
(2) Use 'Export to FASTA' option in the IrysView software to export the hybrid assembly into a FASTA file.
(3) Identify the set of contigs (as cMAP IDs) that were filtered out during the scaffolding process, through a search of the intermediate results in hybrid scaffolding, and back-track the identifier of the contigs through the key file generated within the intermediate results
(4) retrieve these 'discarded' contigs from the original FASTA file from PacBio assembly, and supplement them into the hybrid assembly to generate the final assembly.

Analysis of genomic variants

Summary of variant calling

Variant calling was performed on Illumina DNA sequencing data. GATK and Freebayes were both used for variant discovery for HX1 genome to ensure consistence of variants generated. 4,413,999 consensus variants, including 625,690 Indels, 3,518,309 SNVs, and 421,285 variants with MAF<=0.01, were used for annotation and downstream analysis. Among exonic SNVs, 9,603 were nonsynonymous, 10,942 were synonymous, 70 stopgain and 7 stoploss variants. We compared the mutational profile of these consensus SNVs with those from some previously published genome assemblies, including AK1, HuRef and YH, and highly confident SNV calls from NA12878 generated by Illumina.

Genome comparisons

RefSeq analysis

Human RefSeq transcripts of type "known" (with NM or NR prefixes[9]) were queried from NCBI Entrez on November 27th, 2015, and aligned to the HX1 assembly, and to GRCh38.p2 (GCF_000001405.28), YH_2.0 (GCA_000004845.2)and ASM101398v1 (GCA_001013985.1) for comparison. The coding transcripts and non-coding transcripts longer than 300 bp were first aligned with Blast (e-value of 0.0001, word size 28 and best-hits options best_hit_overhang=0.1 and best_hit_score_edge=0.1) to the four genomes masked with RepeatMasker[10] or Windowmasker[11]. Sets of results obtained with both masking methods were passed to the global alignment algorithm Splign[12] (75% min exon identity, 50% min compartment identity and 20% min singleton identity) to refine the splice junctions and align exons missed by Blast. Sequences for which no alignment with coverage higher than 95% of the query, and sequences with unaligned overhangs at the 5' or 3' end were re-aligned with Blast and Splign to the unmasked genome and then submitted to the same filter. Non-coding transcripts shorter than 300 bp were aligned with Blast to the unmasked genome (evalue of 0.0001, word size 16, 98%

identity and best-hits options best_hit_overhang=0.1 and best_hit_score_edge=0.1) and then with Splign (75% min exon identity, min compartment identity and min singleton identity) and submitted to the same filter as the other transcripts. The alignments for each transcript were then ranked based on identity and coverage.

As a measure of the relative quality of the assemblies being compared, the counts of transcripts for which no alignment met the filter were generated for each assembly. In addition, the number of transcripts split across multiple genomic locations and the number of low-CDS-coverage transcripts were identified, respectively, as the transcripts for which several rank 1 alignments that don't overlap on the query were available, and as the coding transcripts for which the longest alignment covered less than 95% of the CDS.

The best-ranking alignments for all transcripts were also evaluated together at each genomic location, to identify genomic regions that may be collapsed in some assemblies but not others. Since each RefSeq transcript is associated with a single gene[13] and genes are not expected to overlap, regions of conflict where transcripts from multiple genes co-aligned were identified and the number of transcripts needed to be dropped to resolve the conflicts was counted for each assembly.

Consensus quality and scaffolding accuracy analysis

Assembly consensus quality was evaluated using MUMmer (nucmer, delta-filter and dnadiff). Scaffolding accuracy was evaluated following Pendleton's method[8] with a few modifications. Sliding 100Kb windows (from start to end) were selected in the assembly to be evaluated. 250bp sequences at head and tail of each window were extracted and aligned to GRCh38 main chromosomes using BWA-MEM. Among uniquely aligned pairs of reads with mapping quality > 30, if two reads are not within 90 Kb or 110Kb to each other, the corresponding window was considered mis-joined.

Short read polishing

Illumina short reads were aligned to HX1 contigs with BWA-MEM. SAMtools was used to call indel and SNVs. Each homozygous (heterozygosity rate<0.1) indel or SNV with genotype quality > 30 was considered as an error in the assembly. HX1 contigs were polished by replacing reference alleles with corresponding alternative alleles to the assembly at each variant site. This procedure was repeated three times to achieve higher consensus quality and lower indel error rate.

Gap filling in GRCh38

We developed a Gap Filling by Assembly (GFA) procedure for closing gaps in the reference genome. Any region consisting of continuous runs of N in the target assembly (GRCh38) is defined as a gap in our method. Therefore, gaps between scaffolds are not considered in our analysis. Gaps within 500bp to each other are merged. Flanking sequences upstream and downstream of the gaps were mapped to the source assembly (HX1). If two anchor sequences for the same gap can both be aligned, they will be examined to remove discordant pairs which

include those alignments with inconsistent orientation, on different contigs, or overlapping with each other. If only one anchor can be aligned, then the anchor will be extended into the gap region wherever possible. Code used for gap filling has been deposited to github (https://github.com/WGLab/uniline).

For each closable gap, a probability is calculated based on the mapping score and gap length. A brief description is shown below.

$$P: this\ gap\ is\ incorrectly\ filled$$
$$P_{a1}: anchor1\ mapping\ is\ wrong$$
$$P_{a2}: anchor2\ mapping\ is\ wrong$$
$$P_g: predicted\ gap\ is\ equal\ to\ original\ gap\ by\ chance$$
$$P = P_{a1}P_{a2}P_g$$

When only single anchor is mapped:

$$P = \min(P_{a1}, P_{a2})$$

P_{a1} and P_{a2} come from mapping quality score of BWA-MEM algorithm, which takes both alignment quality and sequence context into consideration. We make the assumption that mapping and gap length are independent of each other. This assumption does not necessarily hold in all circumstances, and can be improved by explicitly modeling the insertion and deletion length in the target genome (where gaps are to be filled).

P_g(predicted gap is equal to original gap by chance) is calculated as follows.

$$L_0: length\ of\ gap\ to\ be\ filled$$
$$L_g: length\ of\ predicted\ gap$$
$$k: tolerance\ factor$$

Let $d = L_g - L_0$. We assumed$d \sim N(0, k^2L_0^2)$, that is we model the difference between predicted gap length and original gap length with a normal distribution. Therefore we have

$$P_g = \begin{cases} P\left(-\left|L_g - L_0\right| \le d \le \left|L_g - L_0\right|\right) L_g \ne L_0 \\ P(-0.5 \le d \le 0.5) L_g = L_0 \end{cases}$$

Intuitively, P_g means the (random) chance observing a gap with L_g or less extreme given L_0 under a normal distribution. The following table shows how the model behaves under some common situations (k=2). As is shown, the model permits ~10% of flexibility at a threshold of 30 and does not penalize harshly when L_g deviates two to three times from L_0.

L_0	1000					
L_g	500	900	1000	2000	2500	3000
Phred-scaled P_g(rounded to nearest integer)	16	32	85	10	6	4

In our analysis, k = 2 was used to calculate gap closing score. Only uniquely closable gaps were shown in results, i.e., flanking sequences of these gaps were uniquely mapped. Gap closing score of 30 were used to filter out low quality predictions.

Finding novel sequences and calculating genome coverage

MUMmer[14-16] is among the first programs that utilizes suffix-tree algorithm to find maximal matches. The time requirement for building suffix-tree for a sequence of length n is of O(n). Given a suffix tree of S and a query sequence Q of length m, all unique maximal matches can be found in time proportional to m. The architecture of MUMmer makes it extremely fast for genome-scale sequence comparison. For each contig in GRCh38 including alt loci (http://hgdownload.cse.ucsc.edu/goldenpath/hg38/bigZips/analysisSet/) and decoy sequences (hs38d1), we mapped our contigs with MUMmer. The results (*.coords) were post-processed by custom scripts to filter and extract regions unmappable with >80% identity and gap allowance of 1kb. Here gap allowance means maximum gap length allowed between two closely mapped regions that will be merged together. However, MUMmer is not as sensitive as BLAST[17] or its descendent, LastZ[18], for genome-scale comparison, so a second round of mapping by LastZ was used to further refine unmapped sequences. LastZ is a successor for BLASTZ[19,20], which is an independent implementation of BLAST[17]. BLASTZ[19,20] differs from BLAST primarily in two aspects: BLASTZ has an option that requires the alignments must occur in the same order and orientation in both reference and query sequences; BLASTZ uses a different scoring scheme that takes sequence conservation into consideration. LastZ[18] was developed based on BLASTZ and is more robust and flexible. LastZ was run with parameters lenient for gap extension, repetitive sequences. Hence large gaps that are possibly structural variations would not hinder alignment, and repetitive sequences do not cause significant slowdown of alignment speed. Regions unmapped by LastZ with minimum alignment length of 250bp gap allowance of 100bp are considered as novel sequences.

Percentage of coverage on GRCh38 (no_alt_analysis) by different assemblies is calculated based off of the results from MUMmer. Same alignment procedure was used as described for finding novel sequence. Regions mapped with >80% identity and gap allowance of 1kb are considered as valid alignments. Percentage of non-N base pairs covered by these valid mappings is denoted as percentage of coverage.

The commands used for MUMmer and LastZ are shown as follows. Custom scripts are deposited in github (https://github.com/WGLab/uniline).

```
#commands for mummer
nucmer -c 400 -l 150 --prefix=$prefix $ref $query
show-coords -r -c -l -k $prefix.delta> $prefix.coords
#commands for LastZ
lastz $ref $query --notransition --gap=1000,1 --step=20 --ambiguous=iupac --format=maf --
gappedthresh=10000 --identity=90 --coverage=90 --progress=10 --maxwordcount=1 --masking=0
```

Comparative analysis using NIST Chinese genome

To assess quality of HX1 and GRCh38 as reference genome for variant calling, we downloaded a Chinese sample NA24694 HiSeq reference data at http://ftp-trace.ncbi.nlm.nih.gov/giab/ftp/data/ChineseTrio/HG006_NA24694-

huCA017E_father/NA24694_Father_HiSeq100x/NA24694_Father_HiSeq100x_fastqs/ from National Institute of Standards and Technology (NIST). In total, we obtained 907 million reads (133 billion bases), with an estimated coverage of 41X. The raw FASTQ files were aligned to both HX1 and GRCh38 by BWA. Reads that cannot be mapped to GRCh38 were further mapped against HX1.

Functional analysis on novel sequence elements

To explore the potential biological function in the novel sequences, we used GM12878 cell line and downloaded 17 of its associated ChIP-Seq data sets with four assays, including H3K4me3, H3K4me1, H3K27ac and CTCF, and 7 DNase-Seq data sets for the same cell line.

The raw FASTQ files were aligned to GRCh38 using bowtie2 (2.0.5)[21]. To investigate whether HX1 can be a complementary reference to GRCh38 for mapping sequencing reads for nucleosome positioning, transcriptional factor binding sites and histone modification, we then extracted unmapped ENCODE reads from GRCh38 and realigned them to HX1 with bowtie2.

MACS[22,23] was used to call ChIP-seq peaks and DNase hot spots. We used settings recommended by authors of MACS. Specifically, for histone mark ChIP-seq, we used “--nomodel” option plus a control data set; for transcription factor, we used default parameters plus a control data set; for DNase-seq, we used “--nolambda” option plus no control data.

To examine how these regulatory elements can potentially modify gene expression for HX1, we performed transcriptome analysis using Illumina RNA-Seq data as follows. First, in order to assess the expression level for each transcript in HX1, we used Cufflinks to generate transcriptome assemblies and used FPKM from this assembly to assess the expression level of each gene predicted by Cufflinks. We examined the distribution of FPKM values of all 33,066 genes predicted by Cufflinks. Note that here we used cutoff of 500bp to ensure that all predicted genes are likely to be real genes, rather than short artifacts generated from Cufflinks prediction. From the pattern of the distribution FPKM values of all predicted genes, we observed that after natural log transformation, the distribution of FPKM values follow roughly Gaussian distribution with several outliers in the tail. Therefore, we excluded additional 94 potential outliers using 99.99% percentile (FPKM=22,026, natural log FPKM=10) as cutoff and used the remaining 27,674 transcripts for downstream analysis. Then, for each regulatory element, we extracted the FPKM value for nearest gene that is located within its 500kb flanking regions (250kb upstream and 250kb downstream) and compared it with the background FPKM value of whole transcriptome in HX1. Here we define background gene set as set of genes that are not flanking CTCF binding sites, DNase I hypersensitivity sites, H3K4me1, H3K4me3 or H3K27ac histone marks. We found that there are 14,133 unique genes located within 500kb flanking regions of CTCF binding sites, 22,506 unique genes near DNase I hypersensitivity sites, 4,557 genes near H3K4me1 histone modification sites, 17,772 genes near H4K4me3 sites and 14,287 genes near H3K27ac sites. Overall, genes flanking regulatory regions in HX1 have increased expression level compared to the background. For histone modifications, H3K4me1 has the highest median FPKM value for its regulated genes (median FPKM=3.72) compared to

the background (median FPKM=2.74) and such difference is significant ($P<10^{-10}$ by Wilcoxon Rank-sum test); H3K4me3 has the lowest median FPKM value for its regulated genes (median FPKM=3.36) and yet such expression level is still significantly higher than the background ($P<5.6X10^{-8}$ by Wilcoxon Rank-sum test). This may imply that in HX1 sequences, there could exist activated enhancers that increase the expression of nearby genes. Similarly, for genes near DNase I hypersensitivity sites and genes near CTCF-binding sites, their expression is also significantly higher than the background ($P<10^{-10}$ by Wilcoxon Rank-sum test for both DNase and CTCF assay).

Detection and comparison of structural variation (SV)

SV calling (Illumina whole-genome sequencing)

To detect CNVs from the Illumina WGS data, the read depth based tool CNVnator was used, which uses a Mean-shift Algorithm, to iteratively partition and merge genomic segments for multiple steps [24].

For whole-genome sequencing data, all the Illumina short reads were first aligned ontoGRCh38 genome, with BWA-MEM[25], to generate the BAM file. Afterwards, the BAM file was used as input into CNVnator[24], to generate CNV calls, with the bin size set as 100 bp. The output is a list of CNVs, with 37,665 deletions and 1,391 duplications. After filtration with P<0.01 standard and removal of CNVs overlapping with Immunoglobulin regions and centromeres, there are in total 2403 deletions and 783 duplications left.

SV calling (BioNano genome map)

Through comparison to an expected digestion map for the GRCh38 genome, the BioNano Genome Map data can be used to identify structural variations. Using the IrysView software, we performed a comparison of the cMAP (expected 7-mer maps digested by enzyme) of our BioNano assembly with GRCh38, and identified 783 insertions, 377 deletions in the HX1 genome in comparison to GRCh38.

SV calling (SMRT long reads)

We identified structural variants >=50 bp from PacBiolong-read sequencing data using FES-SV as previously described [26]. Briefly, we aligned the PacBio reads to GRCh38 using a modified version of BLASR (https://github.com/mchaisso/blasr) with affine alignment parameters (-bestn 2 -maxAnchorsPerPosition 100 -advanceExactMatches 10 -affineAlign -affineOpen 100 -affineExtend 0 -insertion 5 -deletion 5 -extend -maxExtendDropoff 50) to identify 49,054 candidate sites of structural variation. We then locally assembled all reads mapping to each candidate site using MHAP and Celera (8.3rc1) and aligned each local assembly to its original

locus using BLASR with refined affine alignment parameters (-affineAlign -affineOpen 8 -affineExtend 0 -bestn 1 -maxMatch 30 -sdpTupleSize 13) to identify the precise breakpoints of all structural variants. Additionally, we performed the same local assemblies in 152,251 sliding windows across the genome (60 Kbpwithi 20 Kbp slide) to discover any additional variants that might have been missed by the initial candidate discovery. Finally, we annotated the repetitive content of the sequence associated with each insertion and deletion using RepeatMasker (3.3.0) with sensitive alignment parameters (-xsmall -no_is -e wublast -s).

Transcriptome analysis and characterization

Iso-Seq CCS data processing

We obtained the raw Iso-Seq data as bax.h5 files, and processed these raw data by the SMRT Portal (the RS_IsoSeq protocol) to circular consensus sequencing (CCS) reads. Reads of insert were classified into full-length or non-full-length, chimeric or non-chimeric reads. The mean lengths of insert for 3-5kb and >5kb are slightly lower than the expected value, so we diagnosed the read length distribution for all reads and for full-length non-chimeric reads. It appears that a portion of the reads from the 3-5kb and >5kb library have lower than expected length and forms a separate peak in the histogram, suggesting that some shorter RNA molecules were included during the library construction process. Possible reason is that Iso-Seq libraries with large sizes are generally more difficult to make due to 5' degradation and incomplete separation by BluePippin.

Error correction for Iso-Seq reads using Illumina short reads

Error correction of all Iso-Seq reads was performed using LSC, following similar steps in its original publication [27]. LSC is an algorithm designed for improving PacBiolong read (LR) accuracy by short read alignment from Illumina RNA-Seq. In brief, all long reads and short reads were first compressed using Homopolymer Compression (HC) algorithm. Next, all compressed long reads were concatenated into human chromosome-sized reference sequences with n bp poly-N inserts between successive LRs, where n is the length of original short reads. Then all short reads were aligned to the human chromosome-sized reference sequences using Bowtie2[21] and correction of long reads was performed at four types of correction points, including HC points, mismatch points, insertion points and deletion points. Finally, all corrected long reads were decompressed from the left-most short-reads-covered points to the right-most short-reads-covered points, generating the final corrected long reads.

There are three measurements commonly used for measuring quality of error correction. First is the percentage of reads retrieved from correction, second is the fraction of length of the

trimmed (corrected) reads compared to the original ones and third is the coverage of short reads on corrected long reads (percentage of corrected long reads covered at least once by short reads that are used to correct them). After error correction, we retrieved high quality corrected long reads from original reads and used these corrected long reads for downstream analysis.

Novel transcript discovery

We first performed isoform-level clustering using the RS_IsoSeqprotocol. This protocol essentially performs isoform-level clustering (ICE) and polishing the results with Quiver (see below for illustration, available at https://github.com/PacificBiosciences/cDNA_primer/wiki/RS_IsoSeq-(v2.3)-Tutorial-%232.-Isoform-level-clustering-(ICE-and-Quiver).

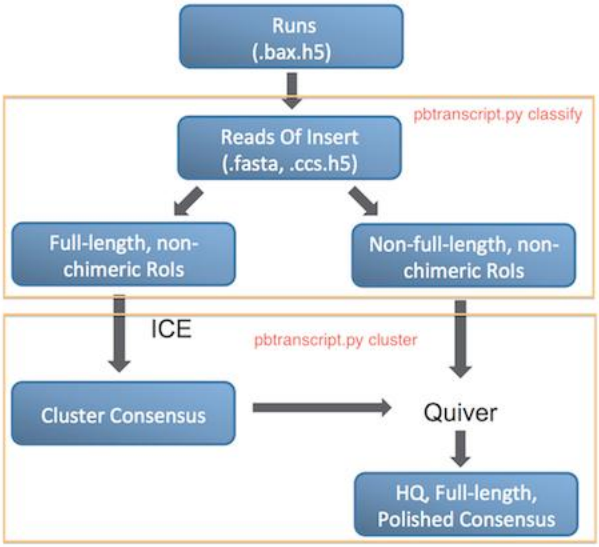

The output from ICE algorithm contains consensus sequences from full-length reads. Each full-length read belongs to exactly one cluster, and each cluster contains one or more full-length reads. At the Quiver step, no new clusters (isoforms) are created. Instead, we are trying to increase the coverage of each isoform by using non-full-length reads. Quiver generates the output quality QV value, which indicates how confident the consensus calls are. The Quiver polished output is classified into either "low QV" or "high QV". See below for illustration, available at https://github.com/PacificBiosciences/cDNA_primer/wiki/RS_IsoSeq-(v2.3)-Tutorial-%232.-Isoform-level-clustering-(ICE-and-Quiver). Our analysis focused on the high-QC consensus isoform clusters, where "Quiver high QV" is currently set with an expected consensus accuracy of 99%.

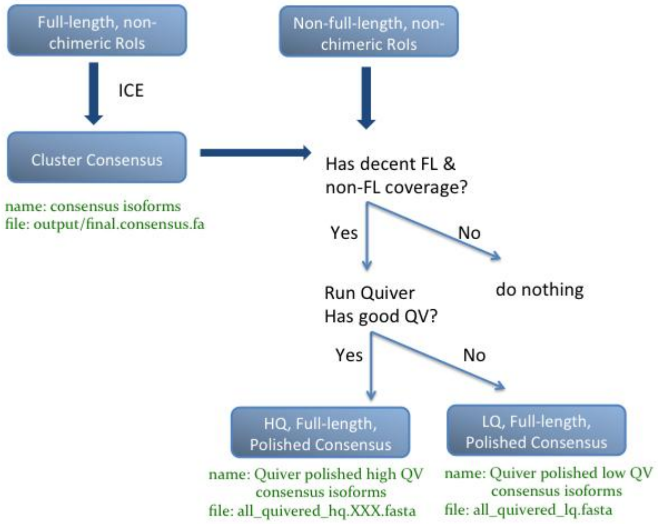

Once we obtained the high-quality consensus clusters, we further aligned them to the GRCh38 reference genome using the GMAP[28] algorithm. For libraries of different sizes, the alignment were performed on clusters from each library, and then combined together. We used Cufflinks to merge all the isoform alignment together into one final GTF file, which is subject to downstream analysis to find novel transcripts. This analysis predicted 58,383 high-quality consensus isoforms at 30,006 loci.

To quantify the read count for each of the isoforms that we found, and to visualize how raw sequencing reads map to the reference genome GRCh38, we also aligned all the error-corrected Iso-Seq reads using GMAP[28] against GRCh38 reference genome. We used HTSeq[29] to count the number of reads that fall within each gene (HTSeq does not support probabilistic assignment of transcripts or isoforms). Visualization wasconducted in the Integrative Genomics Viewer by loading the BAM files directly into the viewer.

We next focused on isoforms in highly expressed genes (that is, those covered by at least 20 reads), and performed comparison of the GTF files with the GENCODE GTF file version 23 (downloaded from http://www.gencodegenes.org/releases/current.html). Cuffcompare[30] was used to compare the two GTF files, and we identified 57 isoforms at 42 loci in IsoSeq GTF that do not overlap with any GENCODE transcripts. The full set of transcripts are given in Supplementary Tables. We experimentally validated several transcripts with more than two predicted exons, by designing pairs of PCR primers that are located in two adjacent exons, and perform PCR reactions on the cDNA samples. The gel bands were cut and DNA was recovered by QiagenQIAquick kit (Valencia, CA, USA), and sent to Sanger sequencing.

Supplementary References

1. Guo, Y., Ding, X., Shen, Y., Lyon, G.J. & Wang, K. SeqMule: automated pipeline for analysis of human exome/genome sequencing data. *Sci Rep* **5**, 14283 (2015).
2. Li, H. & Durbin, R. Fast and accurate short read alignment with Burrows-Wheeler transform. *Bioinformatics* **25**, 1754-60 (2009).
3. McKenna, A. et al. The Genome Analysis Toolkit: a MapReduce framework for analyzing next-generation DNA sequencing data. *Genome Res* **20**, 1297-303 (2010).
4. Wang, K., Li, M. & Hakonarson, H. ANNOVAR: functional annotation of genetic variants from high-throughput sequencing data. *Nucleic Acids Res* **38**, e164 (2010).
5. Untergasser, A. et al. Primer3--new capabilities and interfaces. *Nucleic Acids Res* **40**, e115 (2012).
6. Chin, C.S. et al. Nonhybrid, finished microbial genome assemblies from long-read SMRT sequencing data. *Nat Methods* **10**, 563-9 (2013).
7. Myers, E.W. The fragment assembly string graph. *Bioinformatics* **21 Suppl 2**, ii79-85 (2005).
8. Pendleton, M. et al. Assembly and diploid architecture of an individual human genome via single-molecule technologies. *Nat Methods* **12**, 780-6 (2015).
9. Pruitt, K.D. et al. RefSeq: an update on mammalian reference sequences. *Nucleic Acids Res* **42**, D756-63 (2014).
10. Smit AFA, H.R., Green P. RepeatMasker Open-3.0. (1996-2004).
11. Morgulis, A., Gertz, E.M., Schaffer, A.A. & Agarwala, R. WindowMasker: window-based masker for sequenced genomes. *Bioinformatics* **22**, 134-41 (2006).
12. Kapustin, Y., Souvorov, A., Tatusova, T. & Lipman, D. Splign: algorithms for computing spliced alignments with identification of paralogs. *Biol Direct* **3**, 20 (2008).
13. Brown, G.R. et al. Gene: a gene-centered information resource at NCBI. *Nucleic Acids Res* **43**, D36-42 (2015).
14. Kurtz, S. et al. Versatile and open software for comparing large genomes. *Genome Biol* **5**, R12 (2004).
15. Delcher, A.L. et al. Alignment of whole genomes. *Nucleic Acids Res* **27**, 2369-76 (1999).
16. Delcher, A.L., Phillippy, A., Carlton, J. & Salzberg, S.L. Fast algorithms for large-scale genome alignment and comparison. *Nucleic Acids Res* **30**, 2478-83 (2002).
17. Altschul, S.F. et al. Gapped BLAST and PSI-BLAST: a new generation of protein database search programs. *Nucleic Acids Res* **25**, 3389-402 (1997).
18. Harris, R.S. IMPROVED PAIRWISE ALIGNMENT OF GENOMIC DNA. *PhD Thesis* (2007).
19. Schwartz, S. et al. PipMaker--a web server for aligning two genomic DNA sequences. *Genome Res* **10**, 577-86 (2000).
20. Schwartz, S. et al. Human-mouse alignments with BLASTZ. *Genome Res* **13**, 103-7 (2003).
21. Langmead, B. & Salzberg, S.L. Fast gapped-read alignment with Bowtie 2. *Nat Methods* **9**, 357-9 (2012).
22. Zhang, Y. et al. Model-based analysis of ChIP-Seq (MACS). *Genome Biol* **9**, R137 (2008).
23. Feng, J., Liu, T. & Zhang, Y. Using MACS to identify peaks from ChIP-Seq data. *Curr Protoc Bioinformatics* **Chapter 2**, Unit 2 14 (2011).

24. Abyzov, A., Urban, A.E., Snyder, M. & Gerstein, M. CNVnator: an approach to discover, genotype, and characterize typical and atypical CNVs from family and population genome sequencing. *Genome research* **21**, 974-984 (2011).
25. Li, H. Aligning sequence reads, clone sequences and assembly contigs with BWA-MEM. *arXiv preprint arXiv:1303.3997* (2013).
26. Chaisson, M.J. et al. Resolving the complexity of the human genome using single-molecule sequencing. *Nature* **517**, 608-11 (2015).
27. Au, K.F., Underwood, J.G., Lee, L. & Wong, W.H. Improving PacBio long read accuracy by short read alignment. *PLoS One* **7**, e46679 (2012).
28. Wu, T.D. & Watanabe, C.K. GMAP: a genomic mapping and alignment program for mRNA and EST sequences. *Bioinformatics* **21**, 1859-1875 (2005).
29. Anders, S., Pyl, P.T. & Huber, W. HTSeq--a Python framework to work with high-throughput sequencing data. *Bioinformatics* **31**, 166-9 (2015).
30. Roberts, A., Pimentel, H., Trapnell, C. & Pachter, L. Identification of novel transcripts in annotated genomes using RNA-Seq. *Bioinformatics* **27**, 2325-2329 (2011).
